# Supplementary material for: COVID-19 Pandemic and Remote Consultations in Children: A Bibliometric Analysis
Source: Int J Environ Res Public Health. 2022 Aug 9;19(16):9787. doi: 10.3390/ijerph19169787 (PMC9407809; doi:10.3390/ijerph19169787)
Supplement: Supplementary file 1 [file ijerph-19-09787-s001.zip › ijerph-1787768-supplementary.pdf]

Supplementary files for Covid-19 pandemic and remote consultations in children: a bibliometric analysis.

Table S1. Preferred Reporting Items for Systematic reviews and Meta-Analyses extension for Scoping Reviews (PRISMA-ScR) Checklist

SECTION	ITEM	PRISMA-ScR CHECKLIST ITEM	REPORTED ON PAGE #
**TITLE**			
Title	1	Identify the report as a scoping review.	1
**ABSTRACT**			
Structured summary	2	Provide a structured summary that includes (as applicable): background, objectives, eligibility criteria, sources of evidence, charting methods, results, and conclusions that relate to the review questions and objectives.	1
**INTRODUCTION**			
Rationale	3	Describe the rationale for the review in the context of what is already known. Explain why the review questions/objectives lend themselves to a scoping review approach.	1-2
Objectives	4	Provide an explicit statement of the questions and objectives being addressed with reference to their key elements (e.g., population or participants, concepts, and context) or other relevant key elements used to conceptualize the review questions and/or objectives.	2-3
**METHODS**			
Protocol and registration	5	Indicate whether a review protocol exists; state if and where it can be accessed (e.g., a Web address); and if available, provide registration information, including the registration number.	3
Eligibility criteria	6	Specify characteristics of the sources of evidence used as eligibility criteria (e.g., years considered, language, and publication status), and provide a rationale.	3
Information sources *	7	Describe all information sources in the search (e.g., databases with dates of coverage and contact with authors to identify additional sources), as well as the date the most recent search was executed.	3-4
Search	8	Present the full electronic search strategy for at least 1 database, including any limits used, such that it could be repeated.	4
Selection of sources of evidence †	9	State the process for selecting sources of evidence (i.e., screening and eligibility) included in the scoping review.	4-5
Data charting process ‡	10	Describe the methods of charting data from the included sources of evidence (e.g., calibrated forms or forms that have been tested by the team before their use, and whether data charting was done independently or in duplicate) and any processes for obtaining and confirming data from investigators.	5

SECTION	ITEM	PRISMA-ScR CHECKLIST ITEM	REPORTED ON PAGE #
Data items	11	List and define all variables for which data were sought and any assumptions and simplifications made.	5
Critical appraisal of individual sources of evidence §	12	If done, provide a rationale for conducting a critical appraisal of included sources of evidence; describe the methods used and how this information was used in any data synthesis (if appropriate).	5
Synthesis of results	13	Describe the methods of handling and summarizing the data that were charted.	4-5
**RESULTS**			
Selection of sources of evidence	14	Give numbers of sources of evidence screened, assessed for eligibility, and included in the review, with reasons for exclusions at each stage, ideally using a flow diagram.	5-6
Characteristics of sources of evidence	15	For each source of evidence, present characteristics for which data were charted and provide the citations.	5-6-
Critical appraisal within sources of evidence	16	If done, present data on critical appraisal of included sources of evidence (see item 12).	5-6
Results of individual sources of evidence	17	For each included source of evidence, present the relevant data that were charted that relate to the review questions and objectives.	6-7-8-9-10
Synthesis of results	18	Summarize and/or present the charting results as they relate to the review questions and objectives.	6-7-8-9-10
**DISCUSSION**			
Summary of evidence	19	Summarize the main results (including an overview of concepts, themes, and types of evidence available), link to the review questions and objectives, and consider the relevance to key groups.	10-11
Limitations	20	Discuss the limitations of the scoping review process.	11
Conclusions	21	Provide a general interpretation of the results with respect to the review questions and objectives, as well as potential implications and/or next steps.	11
**FUNDING**			
Funding	22	Describe sources of funding for the included sources of evidence, as well as sources of funding for the scoping review. Describe the role of the funders of the scoping review.	NA

JBI = Joanna Briggs Institute; PRISMA-ScR = Preferred Reporting Items for Systematic reviews and Meta-Analyses extension for Scoping Reviews.

* Where *sources of evidence* (see second footnote) are compiled from, such as bibliographic databases, social media platforms, and Web sites.

† A more inclusive/heterogeneous term used to account for the different types of evidence or data sources (e.g., quantitative and/or qualitative research, expert opinion, and policy documents) that may be eligible in a scoping review as opposed to only studies. This is not to be confused with *information sources* (see first footnote).

‡ The frameworks by Arksey and O'Malley (6) and Levac and colleagues (7) and the JBI guidance (4, 5) refer to the process of data extraction in a scoping review as data charting.

§ The process of systematically examining research evidence to assess its validity, results, and relevance before using it to inform a decision. This term is used for items 12 and 19 instead of "risk of bias" (which is more applicable to systematic reviews of interventions) to include and acknowledge the various sources of evidence that may be used in a scoping review (e.g., quantitative and/or qualitative research, expert opinion, and policy document).

Table S2. Included studies

Title	Author Names	Country	Continent	Publication type	Publication Year	Topic	Topic according MESH	Type of study	Citation (bibliographic reference)	Journal/Book	2020 Impact Factor	Category of the Journal in JCR	Times cited in embase /WOS	Type of evaluation	Doi
Feasibility of a Secure Wireless Sensing Smartwatch Application for the Self-Management of Pediatric Asthma	Hosseini, A.; Buonocore, C. M.; Hashemzadeh, S.; Hojaiji, H.; Kalantarian, H.; Sideris, C.; Bui, A. A. T.; King, C. E.; Sarrafzadeh, M.	United States of America	North America	Article	2017	Asthma	Asthma	Study protocol/Pilot study	Sensors (Basel). 2017 Aug 3;17(8):1780.	SENSORS (BASEL)	3,576	CHEMISTRY, ANALYTICAL	19	Trearment	10.3390/s17081780
Comparison of dental hygienists and dentists: clinical and teledentistry identification of dental caries in children	Daniel, S. J.; Kumar, S.	United States of America	North America	Article	2017	Caries of deciduous teeth	Dental caries	Observational studies	Int J Dent Hyg. 2017 Nov;15(4):e143-e148	INTERNATIONAL JOURNAL OF DENTAL HYGIENE	2,477	DENTISTRY, ORAL SURGERY & MEDICINE	10	Diagnosis	10.1111/idh.12232
International inter-rater agreement in scoring acne severity utilizing cloud-based image sharing of mobile phone photographs	Foolad, N.; Ornelas, J. N.; Clark, A. K.; Ali, I.; Sharon, V. R.; Al Mubarak, L.; Lopez, A.; Alikhan, A.; Al Dabagh, B.; Firooz, A.; Awasthi, S.; Liu, Y.; Li, C. S.; Sivamani, R. K.	United States of America	North America	Article	2017	Acne vulgaris	Skin diseases	Observational studies	*I*nt J Dermatol. 2017 Sep;56(9):920-925	INTERNATIONAL JOURNAL OF DERMATOLOGY	2,736	DERMATOLOGY	5	Diagnosis	10.1111/ijd.13621
Direct-Access Online Care for the Management of Atopic Dermatitis: A Randomized Clinical Trial Examining Patient Quality of Life	Kornmehl, Heather; Singh, Sanminder; Johnson, Mary Ann; Armstrong, April W.	United States of America	North America	Article	2017	Atopic dermatitis	Skin diseases	Interventional studies	Telemed J E Health. 2017 Sep;23(9):726-732.	TELEMEDICINE AND E-HEALTH	NA	HEALTH CARE SCIENCES & SERVICES	13	Follow up	10.1089/tmj.2016.0249
Improved Medication Adherence and Frequency of Blood Glucose Self-Testing Using an m-Health Platform Versus Usual Care in a Multisite Randomized Clinical Trial Among People with Type 2 Diabetes in India	Kleinman, N. J.; Shah, A.; Shah, S.; Phatak, S.; Viswanathan, V.	India	Asia	Article	2017	Diabetes mellitus type I	Metabolic diseases	Interventional studies	Telemed J E Health. 2017 Sep;23(9):733-740.	TELEMEDICINE JOURNAL AND E-HEALTH	NA	HEALTH CARE SCIENCES & SERVICES	44	Trearment	10.1089/tmj.2016.0265
Sugarsquare, a Web-Based Patient Portal for Parents of a Child With Type 1 Diabetes: Multicenter Randomized Controlled Feasibility Trial	Boogerd, Emiel; Maas-Van Schaaijk, Nienke M.; Sas, Theo C.; Clement-de Boers, Agnes; Smallenbroek, Mischa; Nuboer, Roos; Noordam, Cees; Verhaak, Chris M.	Netherlands	Europe	Article	2017	Diabetes mellitus type I	Metabolic diseases	Interventional studies	J Med Internet Res. 2017 Aug 22;19(8):e28	JOURNAL OF MEDICAL INTERNET RESEARCH	5,428	MEDICAL INFORMATICS	13	Follow up	10.2196/jmir.6639

How do eHealth Programs for Adolescents With Depression Work? A Realist Review of Persuasive System Design Components in Internet-Based Psychological Therapies	Wozney, L.; Huguet, A.; Bennett, K.; Radomski, A. D.; Hartling, L.; Dyson, M.; McGrath, P. J.; Newton, A. S.	Canada	North America	Article	2017	Major depressive disorder	Mental disorders	Review/meta-analysis	J Med Internet Res. 2017 Aug 9;19(8):e266.	JOURNAL OF MEDICAL INTERNET RESEARCH	5,428	MEDICAL INFORMATICS	5	Trearment	10.2196/jmir.7573
A Fully Automated Web-Based Program Improves Lifestyle Habits and HbA1c in Patients With Type 2 Diabetes and Abdominal Obesity: Randomized Trial of Patient E-Coaching Nutritional Support (The ANODE Study)	Hansel, B.; Giral, P.; Gambotti, L.; Lafourcade, A.; Peres, G.; Filipecki, C.; Kadouch, D.; Hartemann, A.; Oppert, J. M.; Bruckert, E.; Marre, M.; Bruneel, A.; Duchene, E.; Roussel, R.	France	Europe	Article	2017	Diabetes mellitus type I	Metabolic diseases	Interventional studies	J Med Internet Res. 2017 Nov 8;19(11):e360.	JOURNAL OF MEDICAL INTERNET RESEARCH	5,428	MEDICAL INFORMATICS	33	Trearment	10.2196/jmir.7947
Parent-Mediated Intervention Training Delivered Remotely for Children With Autism Spectrum Disorder Living Outside of Urban Areas: Systematic Review	Parsons, Dave; Cordier, Reinie; Vaz, Sharmila; Lee, Hoe C.	Australia	Oceania	Article	2017	Autism spectrum disorders	Neurodevelopmental diseases or disorders	Review/meta-analysis	J Med Internet Res. 2017 Aug 14;19(8):e198.	JOURNAL OF MEDICAL INTERNET RESEARCH	5,428	MEDICAL INFORMATICS	5	Trearment	10.2196/jmir.6651
Self-Directed Telehealth Parent-Mediated Intervention for Children With Autism Spectrum Disorder: Examination of the Potential Reach and Utilization in Community Settings	Ingersoll, Brooke; Shannon, Katherine; Berger, Natalie; Pickard, Katherine; Holtz, Bree	United States of America	North America	Article	2017	Autism spectrum disorders	Neurodevelopmental diseases or disorders	Observational studies	J Med Internet Res. 2017 Jul 12;19(7):e248.	JOURNAL OF MEDICAL INTERNET RESEARCH	5,428	MEDICAL INFORMATICS	31	Trearment	10.2196/jmir.7484
Targeted Adherence Intervention to Reach Glycemic Control with Insulin Therapy for patients with Diabetes (TARGIT-Diabetes): rationale and design of a pragmatic randomised clinical trial	Lewey, J.; Wei, W.; Lauffenburger, J. C.; Makanji, S.; Chant, A.; DiGeronimo, J.; Nanchanatt, G.; Jan, S.; Choudhry, N. K.	United States of America	North America	Article	2017	Diabetes mellitus type I	Metabolic diseases	Study protocol/Pilot study	BMJ Open. 2017 Oct 30;7(10):e016551.	BMJ OPEN	2,692	MEDICINE, GENERAL & INTERNAL	9	Trearment	10.1136/bmjopen-2017-016551
Design and rationale of the Cardiovascular Health and Text Messaging (CHAT) Study and the CHAT-Diabetes Mellitus (CHAT-DM) Study: two randomised controlled trials of text messaging to improve secondary prevention for coronary heart disease and diabetes	Huo, X.; Spatz, E. S.; Ding, Q.; Horak, P.; Zheng, X.; Masters, C.; Zhang, H.; Irwin, M. L.; Yan, X.; Guan, W.; Li, J.; Li, X.; Spertus, J. A.; Masoudi, F. A.; Krumholz, H. M.; Jiang, L.	multicenter	More than one continent	Article	2017	Diabetes mellitus type I	Metabolic diseases	Interventional studies	BMJ Open. 2017 Dec 21;7(12):e018302.	BMJ OPEN	2,692	MEDICINE, GENERAL & INTERNAL	8	Follow up	10.1136/bmjopen-2017-018302

Diabetes mellitus in children and adolescents: New challenges of telemedicine	Mirza, J.; Mönkemöller, K.; Weiß, M.	Germany	Europe	Article	2017	Diabetes mellitus type I	Metabolic diseases	Review/meta-analysis	Monatsschrift fur Kinderheilkunde 2017 165:8 (688-696)	MONATSSCHRIFT FUR KINDERHEILKUNDE	NA	NA	2	Follow up	10.1007/s00112-017-0334-9
Partnerships with Primary Care for the Treatment of Preschoolers	Marcus, S. M.; Malas, N. M.; Quigley, J. M.; Rosenblum, K. L.; Muzik, M.; LePlatte-Ogini, D. J.; Patel, P. D.	United States of America	North America	Article	2017	Attention-deficit/hyperactivity disorder	Neurodevelopmental diseases or disorders	Review/meta-analysis	Child Adolesc Psychiatr Clin N Am. 2017 Jul;26(3):597-609.	CHILD ADOLESC PSYCHIATR CLIN N AM	NA	NA	6	Diagnosis & Treatment/Follow-up	10.1016/j.chc.2017.03.002
Development of School-Based Asthma Management Programs in Rochester, New York: Presented in Honor of Dr Robert Haggerty	Halterman, Jill S.; Tajon, Reynaldo; Tremblay, Paul; Fagnano, Maria; Butz, Arlene; Perry, Tamara T.; McConnochie, Kenneth M.	United States of America	North America	Article	2017	Asthma	Asthma	Study protocol/Pilot study	Acad Pediatr. 2017 Aug;17(6):595-599.	ACADEMIC PEDIATRICS	3,107	PEDIATRICS	11	Trearment	10.1016/j.acap.2017.04.008
Evaluation of a telemedicine program for pediatric diabetic retinopathy	Strul, S.; Donahue, S.; Datye, K.; Russell, W.	United States of America	North America	Conference abstract	2017	Diabetes mellitus type I	Metabolic diseases	Observational studies	Strul, S., Donahue, S., Datye, K., & Russell, W. (2017). Evaluation of a telemedicine program for pediatric diabetic retinopathy. Journal of American Association for Pediatric Ophthalmology and Strabismus {JAAPOS}, 21(4), e13.	JOURNAL OF AAPOS	1,22	PEDIATRICS	0	Follow up	10.1016/j.jaapos.2017.07.039
Telemedicine is helping the parents of children with neurodevelopmental disorders living in remote and deprived areas	Stuckey, R.; Domingues-Montanari, S.	Spain	Europe	Comment/editorial/letter	2017	Autism spectrum disorders	Mental disorders	Review/meta-analysis	Stuckey, R., & Domingues-Montanari, S. (2017). Telemedicine is helping the parents of children with neurodevelopmental disorders living in remote and deprived areas. Paediatrics and International Child Health, 37(3), 155-157.	PAEDIATRICS AND INTERNATIONAL CHILD HEALTH	1,990	PEDIATRICS	7	Trearment	10.1080/20469047.2017.1315914

Direct-to-patient teledermatology mobile health app aimed at shortening wait times for new patients	Jew, O.; McMahon, P.; Fiks, A.; Berrigan, L.; Sykes, E.; Gruver, R.; Halkyard, K.; Winston, F.; Fleisher, L.	United States of America	North America	Conference abstract	2017	Acne vulgaris	Skin diseases	Interventional studies	Pediatric Dermatology 20 17 34 Suppleme nt 1 (S77-)	PEDIATRIC DERMATOLO GY	1,588	PEDIATRICS	0	Follow up	10.1111/pde. 13195
Reducing hypoglycemia fear in parents of young kids with type 1 diabetes (T1D) using video-based telemedicine: Preliminary findings from REDCHiP	Patton, S.; Noser, A.; Marker, A.; Calkins, A.; Nelson, E. L.; Clements, M.	United States of America	North America	Conference abstract	2017	Diabetes mellitus type I	Metabolic diseases	interventional studies	Pediatric diabetes 2017; 18(suppl 25):19	PEDIATRIC DIABETES	4,866	PEDIATRICS	0	trearment	10.1111/pedi. 12587
Home telemedicine clinic model significantly improves confidence, self-efficacy and communication in young adults with type 1 diabetes	Raymond, J.; Cain, C.; Berget, C.; Ketchum, K.; Reid, M.; Thomas, J.; Klingensmith, G.	United States of America	North America	Article	2017	Diabetes mellitus type I	Metabolic diseases	Observational studies	Diabetes Educ. 2019 Aug;45(4):420-430	PEDIATRIC DIABETES	4,866	PEDIATRICS	26	Follow up	10.1111/pedi. 12589
The Use of Telemedicine and Mobile Technology to Promote Population Health and Population Management for Psychiatric Disorders	Turvey, Carolyn; Fortney, John	United States of America	North America	Article	2017	Attention-deficit/hy peractivit y disorder	Mental disorders	Review/meta-analysis	Turvey, C., Fortney, J. The Use of Telemedicine and Mobile Technology to Promote Population Health and Population Management for Psychiatric Disorders. Curr Psychiatry Rep 19, 88 (2017). https://doi.org/ 10.1007/s11920-017-0844-0	CURRENT PSYCHIATRY REPORTS	5,285	PSYCHIATRY	15	Trearment	10.1007/s119 20-017-0844-0
Efficacy of an internet-based CBT program for children with comorbid High Functioning Autism Spectrum Disorder and anxiety: A randomised controlled trial	Conaughton, R. J.; Donovan, C. L.; March, S.	Australia	Oceania	Article	2017	Autism spectrum disorders	Neurodev elopmenta l diseases or disorders	Interventional studies	J Affect Disord. 2017 Aug 15;218:260-268	JOURNAL OF AFFECTIVE DISORDERS	4,839	PSYCHIATRY	36	Trearment	10.1016/j.jad. 2017.04.032
The Children's Attention-Deficit Hyperactivity Disorder Telemental Health Treatment Study: Caregiver Outcomes	Vander Stoep, A.; McCarty, C. A.; Zhou, C.; Rockhill, C. M.; Schoenfelder, E. N.; Myers, K.	United States of America	North America	article	2017	Attention-deficit/hy peractivit y disorder	Mental disorders	Interventional studies	J Abnorm Child Psychol. 2017 Jan;45(1):27-43	JOURNAL OF ABNORMAL CHILD PSYCHOLOGY	3,837	PSYCHOLOGY, CLINICAL	15	Trearment	10.1007/s108 02-016-0155-7
Delivery of a Therapist-Facilitated Telecare Anxiety Program to Children in Rural Communities: A Pilot Study	McLellan, Lauren F.; Andrijic, Vanessa; Davies, Suzanne; Lyneham, Heidi J.; Rapee, Ronald M.	Australia	Oceania	Article	2017	Anxiety disorders	Mental disorders	Study protocol/Pilot study	Behaviour Change 2017 34:3 (156-167)	BEHAVIOUR CHANGE	1,469	PSYCHOLOGY, CLINICAL	6	Trearment	10.1017/bec.2 017.11

A virtual asthma clinic for children: fewer routine outpatient visits, same asthma control	van den Wijngaart, L. S.; Roukema, J.; Boehmer, A. L. M.; Brouwer, M. L.; Hugen, C. A. C.; Niers, L. E. M.; Sprij, A. J.; Rikkers-Mutsaerts, Ervm; Rottier, B. L.; Donders, A. R. T.; Verhaak, C. M.; Pijnenburg, M. W.; Merkus, Pjfm	Netherlands	Europe	Article	2017	Asthma	Asthma	Interventional studies	Van Den Wijngaart, L. S., Roukema, J., Boehmer, A. L., Brouwer, M. L., Hugen, C. A., Niers, L. E., ... & Merkus, P. J. (2017). A virtual asthma clinic for children: fewer routine outpatient visits, same asthma control. European Respiratory Journal, 50(4).	EUROPEAN RESPIRATORY JOURNAL	16,671	RESPIRATORY SYSTEM	29	Follow up	10.1183/13993003.00471-2017
Online asthma management for children is cost-effective	van den Wijngaart, Lara S.; Kievit, Wietske; Roukema, Jolt; Boehmer, Annemie L. M.; Brouwer, Marianne L.; Hugen, Cindy A. C.; Niers, Laetitia E. M.; Sprij, Arwen J.; Rikkers-Mutsaerts, Eleonora R. V. M.; Rottier, Bart L.; Verhaak, Chris M.; Pijnenburg, Marielle W.; Merkus, Peter J. F. M.	Netherlands	Europe	Comment/editorial/letter	2017	Asthma	Asthma	Interventional studies	Van Den Wijngaart, L. S., Kievit, W., Roukema, J., Boehmer, A. L., Brouwer, M. L., Hugen, C. A., ... & Merkus, P. J. (2017). Online asthma management for children is cost-effective. European Respiratory Journal, 50(4).	EUROPEAN RESPIRATORY JOURNAL	16,671	RESPIRATORY SYSTEM	13	Follow up	10.1183/13993003.01413-2017
Perspectives from the Kidney Health Initiative on Advancing Technologies to Facilitate Remote Monitoring of Patient Self-Care in RRT	Rosner, Mitchell H.; Lew, Susie Q.; Conway, Paul; Ehrlich, Jennifer; Jarrin, Robert; Patel, Uptal D.; Rheuban, Karen; Robey, R. Brooks; Sikka, Neal; Wallace, Eric; Brophy, Patrick; Sloand, James	United States of America	North America	Article	2017	Diabetes mellitus type I	Metabolic diseases	Review/meta-analysis	Clin J Am Soc Nephrol. 2017 Nov 7;12(11):1900-1909	CLINICAL JOURNAL OF THE AMERICAN SOCIETY OF NEPHROLOGY	8,237	UROLOGY & NEPHROLOGY	33	Follow up	10.2215/CJN.12781216
Asthma mobile applications: Are they ready for prime time?	Blaiss MS.	United States of America	North America	Comment/editorial/letter	2018	Asthma	Asthma	Review/meta-analysis	Ann Allergy Asthma Immunol. 2018 Apr120(4):347-348	ANNALS OF ALLERGY, ASTHMA & IMMUNOLOGY	6,347	ALLERGY	4	Diagnosis & Treatment/Follow-up	10.1016/j.anai.2018.02.002
Learnings from a pragmatic pilot trial of text messaging for high-risk adolescents with asthma	Dodds CM, Britto MT.	United States of America	North America	Article	2018	Asthma	Asthma	Interventional studies	Ann Allergy Asthma Immunol. 2018 May120(5):546-547	ANNALS OF ALLERGY, ASTHMA & IMMUNOLOGY	6,347	ALLERGY	2	Follow up	10.1016/j.anai.2018.02.008

Kiss myAsthma: Using a participatory design approach to develop a self-management app with young people with asthma	Davis SR, Peters D, Calvo RA, Sawyer SM, Foster JM, Smith L.	Australia	Oceania	Article	2018	Asthma	Asthma	Interventional studies	J Asthma. 2018 Sep55(9):1018-1027	JOURNAL OF ASTHMA	2,515	ALLERGY	22	Treatment	10.1080/02770903.2017.1388391
High-quality pediatric spirometry via telemedicine	Perry T.T., Leisenring P.K., Harwell S.A., Chervinskiy S.K., Simmons L.A., Jones S.M., Carroll J.L., Berlinski A.	United States of America	North America	Conference abstract	2018	Asthma	Asthma	Interventional studies	Journal of Allergy and Clinical Immunology (2018) 141:2 Supplement 1 (AB103)	JOURNAL OF ALLERGY AND CLINICAL IMMUNOLOGY	10,793	ALLERGY	0	Follow up	https://doi.org/10.1016/j.jaci.2017.12.329
Tele-EEG: Current Applications, Challenges, and Technical Solutions	Rosenow F., Audebert H.J., Hamer H.M., Hinrichs H., Keler-Uberti S., Kluge T., Noachtar S., Remi J., Sotoodeh A., Strzelczyk A., Weber J.E., Zöllner J.P.	Germany	Europe	Article	2018	Epilepsy	Brain disorders	Review/meta-analysis	Klinische Neurophysiologie (2018) 49:4 (208-215). Date of Publication: 2018	KLINISCHE NEUROPHYSIOLOGIE	0,27	CLINICAL NEUROLOGY	7	Diagnosis & Treatment/Follow-up	10.1055/a-0627-8047
Computer-assisted rehabilitation of attention in pediatric multiple sclerosis and ADHD patients: a pilot trial	Simone M, Viterbo RG, Margari L, Iaffaldano P.	Italy	Europe	Article	2018	Conduct disorder	Neurodevelopmental diseases or disorders	Interventional studies	BMC Neurol. 2018 Jun 818(1):82	BMC NEUROLOGY	2,474	CLINICAL NEUROLOGY	14	Treatment	10.1186/s12883-018-1087-3
A resource reallocation model for school dental screening: taking advantage of teledentistry in low-risk areas	Estai, M; Bunt, SM; Kanagasingam, Y; Kruger, E; Tennant, M	Australia	Oceania	Article	2018	Caries of permanent teeth	Dental caries	Observational studies	Int Dent J. 2018 Aug;68(4):262-268	INTERNATIONAL DENTAL JOURNAL	2,512	DENTISTRY, ORAL SURGERY & MEDICINE	9	Diagnosis	10.1111/idj.12379
Using Network Oriented Research Assistant (NORA) technology to compare digital photographic with in-person assessment of acne vulgaris	Singer H.M., Almazan T., Craft N., David C.V., Eells S., Erfe C., Lazzaro C., Nguyen K., Preciado K., Tan B., Patel V.A.	United States of America	North America	Article	2018	Acne vulgaris	Skin diseases	Study protocol/Pilot study	JAMA Dermatology (2018) 154:2 (188-190)	JAMA DERMATOLOGY	10,282	DERMATOLOGY	12	Diagnosis	10.1001/jamadermatol.2017.5141
Use of the Network-Oriented Research Assistant technology to validate the use of digital photographs in the assessment and quantification of acne vulgaris	Singer H., Almazan T., Craft N., David C.V., Eells S., Erfe C., Nguyen K., Preciado K., Tan B., Patel V.	United States of America	North America	Conference abstract	2018	Acne vulgaris	Skin diseases	Interventional studies	Journal of the American Academy of Dermatology 2018 79:3 Supplement 1 (AB308-)	JOURNAL OF THE AMERICAN ACADEMY OF DERMATOLOGY	11,527	DERMATOLOGY	0	Diagnosis	10.1016/j.jaad.2018.05.1215
Telehealth Parent Training in the Early Start Denver Model: Results From a Randomized Controlled Study	Vismara L.A., McCormick C.E.B., Wagner A.L., Monlux K., Nadhan A., Young G.S.	Canada	North America	Article	2018	Autism spectrum disorders	Neurodevelopmental diseases or disorders	Interventional studies	Focus on Autism and Other Developmental Disabilities (2018) 33:2 (67-79). Date of Publication: 1 Jun 2018	FOCUS ON AUTISM AND OTHER DEVELOPMENTAL DISABILITIES	3,042	EDUCATION, SPECIAL	65	Treatment	10.1177/1088357616651064

An Intervention by a Patient-Designed Do-It-Yourself Mobile Device App Reduces HbA1c in Children and Adolescents with Type 1 Diabetes: A Randomized Double-Crossover Study	Klee P, Bussien C, Castellsague M, Combescure C, Dirlewanger M, Girardin C, Mando JL, Perrenoud L, Salomon C, Schneider F, Schwitzgebel VM.	Switzerland	Europe	Article	2018	Diabetes mellitus type I	Metabolic diseases	Interventional studies	Diabetes Technol Ther. 2018 Dec20(12):797-805	DIABETES TECHNOLOGY & THERAPEUTICS	6,111	ENDOCRINOLOGY & METABOLISM	19	Trearment	10.1089/dia.2018.0255
Diabetes technology in developing countries	Calliari L.E.	Brazil	South America	Conference abstract	2018	Diabetes mellitus type I	Metabolic diseases	Review/meta-analysis	Diabetes Technology and Therapeutics 2018 20 Supplement 1 (A4-A5)	DIABETES TECHNOLOGY & THERAPEUTICS	6,118	ENDOCRINOLOGY & METABOLISM	0	Follow up	10.1089/dia.2018.2525.abstracts
Teleconsultation in type 1 diabetes mellitus (TELEDIABE)	Bertuzzi F, Stefani I, Rivolta B, Pintaudi B, Meneghini E, Luzi L, Mazzone A.	Italy	Europe	Article	2018	Diabetes mellitus type I	Metabolic diseases	Interventional studies	Acta Diabetol. 2018 Feb55(2):185-192	ACTA DIABETOLOGICA	4,28	ENDOCRINOLOGY & METABOLISM	26	Follow up	10.1007/s00592-017-1084-9
Distal technologies and type 1 diabetes management	Duke, DC; Barry, S; Wagner, DV; Speight, J; Choudhary, P; Harris, MA	United States of America	North America	Article	2018	Diabetes mellitus type I	Metabolic diseases	Review/meta-analysis	Lancet Diabetes Endocrinol. 2018 Feb;6(2):143-156	LANCET DIABETES & ENDOCRINOLOGY	32,069	ENDOCRINOLOGY & METABOLISM	32	Trearment	10.1016/S2213-8587(17)30260-7
CoYoT1 Clinic: Home Telemedicine Increases Young Adult Engagement in Diabetes Care	Reid MW, Krishnan S, Berget C, Cain C, Thomas JF, Klingensmith GJ, Raymond JK.	United States of America	North America	Article	2018	Diabetes mellitus type I	Metabolic diseases	Interventional studies	Diabetes Technol Ther. 2018 May20(5):370-379	DIABETES TECHNOLOGY & THERAPEUTICS	6,111	ENDOCRINOLOGY & METABOLISM	32	Follow up	10.1089/dia.2017.0450
Feasibility of shared telemedicine appointments for low SES adolescents and young adults with T1D	Flores Garcia J.J., Reid M.W., Raymond J.	United States of America	North America	Conference abstract	2018	Diabetes mellitus type I	Metabolic diseases	Observational studies	Diabetes 2018 67 Supplement 1 (A355-)	DIABETES	9,461	ENDOCRINOLOGY & METABOLISM	0	Follow up	https://doi.org/10.2337/db18-1325-P
Five-year experience with telemedicine clinics for youth with type 1 diabetes (T1D)	Wadwa R.P., Stacy J., Reznick-Lipina T., Slover R.H., Thomas J.F.	United States of America	North America	Conference abstract	2018	Diabetes mellitus type I	Metabolic diseases	Observational studies	Diabetes (2018) 67 Supplement 1 (A373)	DIABETES	9,461	ENDOCRINOLOGY & METABOLISM	0	Trearment	https://doi.org/10.2337/db18-1389-P
Use of the tidepool platform to collect, integrate, and visualize diabetes device data in a pediatric clinic setting	Wong J.C., Izadi Z., Schroeder S.M., Nader M., Min H.J., Neinstein A.B., Adi S.	United States of America	North America	Conference abstract	2018	Diabetes mellitus type I	Metabolic diseases	Interventional studies	Diabetes 2018 67 Supplement 1 (A244-)	DIABETES	9,461	ENDOCRINOLOGY & METABOLISM	0	Follow up	https://doi.org/10.2337/db18-940-P
A systematic review of the research evidence for the benefits of teledentistry	Estai, M; Kanagasingam, Y; Tennant, M; Bunt, S	Australia	Oceania	Article	2018	Caries of deciduous teeth	Dental caries	Review/meta-analysis	J Telemed Telecare. 2018 Apr;24(3):147-156	JOURNAL OF TELEMEDICINE AND TELECARE	6,184	HEALTH CARE SCIENCES & SERVICES	49	Follow up	10.1177/1357633X16689433

Parental evaluation of a telemonitoring service for children with Type 1 Diabetes	Losiouk E, Lanzola G, Del Favero S, Boscari F, Messori M, Rabbone I, Bonfanti R, Sabbion A, Iafusco D, Schiaffini R, Visentin R, Galasso S, Di Palma F, Chernavvsky D, Magni L, Cobelli C, Bruttomesso D, Quaglini S.	Italy	Europe	Article	2018	Diabetes mellitus type I	Metabolic diseases	Interventional studies	J Telemed Telecare. 2018 Apr24(3):230-237	JOURNAL OF TELEMEDICINE AND TELECARE	6,184	HEALTH CARE SCIENCES & SERVICES	10	Follow up	10.1177/1357633X17695172
Use of Telehealth Videoconferencing in Pediatric Type 1 Diabetes in Oregon	Guttmann-Bauman I, Kono J, Lin AL, Ramsey KL, Boston BA.	United States of America	North America	Article	2018	Diabetes mellitus type I	Metabolic diseases	Observational studies	Telemed J E Health. 2018 Jan24(1):86-88	TELEMEDICINE AND E-HEALTH	3,536	HEALTH CARE SCIENCES & SERVICES	9	Follow up	10.1089/tmj.2017.0072
A Systematic Review on the Validity of Teledentistry	Alabdullah, JH; Daniel, SJ	United States of America	North America	Article	2018	Caries of permanent teeth	Dental caries	Review/meta-analysis	Telemed J E Health. 2018 Aug;24(8):639-648	TELEMEDICINE AND E-HEALTH	3,536	HEALTH CARE SCIENCES & SERVICES	48	Diagnosis	10.1089/tmj.2017.0132
Exploring the Influence of a Smartphone App (Young with Diabetes) on Young People's Self-Management: Qualitative Study	Husted, GR; Weis, J; Teilmann, G; Castensoe-Seidenfaden, P	Danmark	Europe	Article	2018	Diabetes mellitus type I	Metabolic diseases	Observational studies	JMIR Mhealth Uhealth. 2018;6(2):e43	JMIR mHEALTH AND uHEALTH	4,773	HEALTH CARE SCIENCES & SERVICES	19	Follow up	10.2196/mhealth.8876
Testing a Smartphone App (Young with Diabetes) to Improve Self-Management of Diabetes Over 12 Months: Randomized Controlled Trial	Castensoe-Seidenfaden, P; Husted, GR; Jensen, AK; Hommel, E; Olsen, B; Pedersen-Bjergaard, U; Kensing, F; Teilmann, G	Danmark	Europe	Article	2018	Diabetes mellitus type I	Metabolic diseases	Interventional studies	JMIR Mhealth Uhealth. 2018 Jun 26;6(6):e141	JMIR mHEALTH AND uHEALTH	4,773	HEALTH CARE SCIENCES & SERVICES	13	Trearment	10.2196/mhealth.9487
Cost savings from a teledentistry model for school dental screening: an Australian health system perspective	Estai M, Bunt S, Kanagasingam Y, Tennant M.	Australia	Oceania	Article	2018	Caries of permanent teeth	Dental caries	Interventional studies	Aust Health Rev. 2018 Sep42(5):482-490	AUSTRALIAN HEALTH REVIEW	1,99	HEALTH POLICY & SERVICES	23	Diagnosis	10.1071/AH16119
Advancement of teledentistry at the university of rochester's eastman institute for oral health	Kopycka-Kedzierawski D.T., McLaren S.W., Billings R.J.	United States of America	North America	Article	2018	Caries of deciduous teeth	Dental caries	Review/meta-analysis	Health Affairs (2018) 37:12 (1960-1966)	HEALTH AFFAIRS	6,301	HEALTH POLICY & SERVICES	10	Diagnosis & Treatment/Follow-up	10.1377/hlthaff.2018.05102
Relations Between the Use of Electronic Health and the Use of General Practitioner and Somatic Specialist Visits in Patients With Type 1 Diabetes: Cross-Sectional Study	Hansen AH, Broz J, Claudi T, Årsand E.	Norway	Europe	Article	2018	Diabetes mellitus type I	Metabolic diseases	Observational studies	J Med Internet Res. 2018 Nov 720(11):e11322	JOURNAL OF MEDICAL INTERNET RESEARCH	5,428	MEDICAL INFORMATICS	12	Diagnosis & Treatment/Follow-up	10.2196/11322

Remote Collaborative Depression Care Program for Adolescents in Araucanía Region, Chile: Randomized Controlled Trial	Martínez V, Rojas G, Martínez P, Zitko P, Irarrázaval M, Luttges C, Araya R.	Chile	South America	Article	2018	Major depressive disorder	Mental disorders	Interventional studies	J Med Internet Res. 2018 Jan 3120(1):e38	JOURNAL OF MEDICAL INTERNET RESEARCH	5,428	MEDICAL INFORMATICS	11	Trearment	10.2196/jmir.8021
Barriers and Facilitators When Implementing Web-Based Disease Monitoring and Management as a Substitution for Regular Outpatient Care in Pediatric Asthma: Qualitative Survey Study	van den Wijngaart LS, Geense WW, Boehmer AL, Brouwer ML, Hugen CA, van Ewijk BE, Koenen-Jacobs MJ, Landstra AM, Niers LE, van Onzenoort-Bokken L, Ottink MD, Rikkers-Mutsaerts ER, Groothuis I, Vaessen-Verberne AA, Roukema J, Merkus PJ.	Netherlands	Europe	Article	2018	Asthma	Asthma	Observational studies	J Med Internet Res. 2018 Oct 3020(10):e284	JOURNAL OF MEDICAL INTERNET RESEARCH	5,428	MEDICAL INFORMATICS	5	Follow up	10.2196/jmir.9245
E-Health interventions for anxiety and depression in children and adolescents with long-term physical conditions	Thabrew H, Stasiak K, Hetrick SE, Wong S, Huss JH, Merry SN.	United States of America	North America	Article	2018	Anxiety disorders	Mental disorders	Review/meta-analysis	Cochrane Database Syst Rev. 2018 Aug 158(8):CD012489	COCHRANE DATABASE OF SYSTEMATIC REVIEWS	9,289	MEDICINE, GENERAL & INTERNAL	14	Diagnosis & Treatment/Follow-up	10.1002/14651858.CD012489.pub2
Technology-enabled examinations of cardiac rhythm, optic nerve, oral health, tympanic membrane, gait and coordination evaluated jointly with routine health screenings: an observational study at the 2015 Kumbh Mela in India	Shah P, Yauney G, Gupta O, Patalano Ii V, Mohit M, Merchant R, Subramanian SV.	United States of America	North America	Article	2018	Caries of permanent teeth	Dental caries	Interventional studies	BMJ Open. 2018 Apr 208(4):e018774	BMJ OPEN	2,692	MEDICINE, GENERAL & INTERNAL	9	Diagnosis	10.1136/bmjopen-2017-018774
Mobile detection of autism through machine learning on home video: A development and prospective validation study	Tariq Q, Daniels J, Schwartz JN, Washington P, Kalantarian H, Wall DP.	United States of America	North America	Article	2018	Autism spectrum disorders	Neurodevelopmental diseases or disorders	Interventional studies	PLoS Med. 2018 Nov 2715(11):e1002705	PLOS MEDICINE	11,069	MEDICINE, GENERAL & INTERNAL	64	Diagnosis	10.1371/journal.pmed.1002705
Therapeutic educational pathway effect on asthma control: A pilot study	Montalbano L., Cilluffo G., Malizia V., Fasola S., Gentile M., Arrigo M., Guardia D.L., Allegra M., Murgia N., Pichini S., Mancini R., Rotolo M.C., Grutta S.L.	United States of America	North America	Conference abstract	2018	Asthma	Asthma	Study protocol/Pilot study	Contemp Clin Trials. 2021 Apr;103:106284	CONTEMPORARY CLINICAL TRIALS	2,226	MEDICINE, RESEARCH & EXPERIMENTAL	0	Trearment	10.1183/13993003.congress-2018.PA4683
BetaMe: impact of a comprehensive digital health programme on HbA1c and weight at 12 months for people with diabetes and pre-diabetes: study protocol for a randomised controlled trial	Sarfati D, McLeod M, Stanley J, Signal V, Stairmand J, Krebs J, Dowell A, Leung W, Davies C, Grainger R.	New Zeland	Oceania	Article	2018	Diabetes mellitus type I	Metabolic diseases	Interventional studies	Trials. 2018 Mar 519(1):161	TRIALS	2,279	MEDICINE, RESEARCH & EXPERIMENTAL	5	Trearment	10.1186/s13063-018-2528-4

Is it feasible to use smartphone images to perform telediagnosis of different stages of occlusal caries lesions?	Kohara EK, Abdala CG, Novaes TF, Braga MM, Haddad AE, Mendes FM.	Brazil	South America	Article	2018	Caries of deciduous teeth	Dental caries	Interventional studies	PLoS One. 2018 Sep 613(9):e0202116	PLoS ONE	3,24	MULTIDISCIPLINARY SCIENCES	16	Diagnosis	10.1371/journal.pone.0202116
Users' preferences and design recommendations to promote engagements with mobile apps for diabetes self-management: Multi-national perspectives	Adu MD, Malabu UH, Malau-Aduli AEO, Malau-Aduli BS.	Australia	Oceania	Article	2018	Diabetes mellitus type I	Metabolic diseases	Observational studies	PLoS One. 2018 Dec 1013(12):e0208942	PLoS ONE	3,24	MULTIDISCIPLINARY SCIENCES	26	Follow up	10.1371/journal.pone.0208942
Telehealth and autism: A systematic search and review of the literature	Sutherland R, Trembath D, Roberts J.	Australia	Oceania	Article	2018	Autism spectrum disorders	Neurodevelopmental diseases or disorders	Review/meta-analysis	Int J Speech Lang Pathol. 2018 Jun20(3):324-336	INTERNATIONAL JOURNAL OF SPEECH-LANGUAGE PATHOLOGY	NA	NA	69	Diagnosis & Treatment/Follow-up	10.1080/17549507.2018.1465123
Real-world evaluation of a mobile health application in children with asthma	Stukus DR, Farooqui N, Strothman K, Ryan K, Zhao S, Stevens JH, Cohen DM.	United States of America	North America	Article	2018	Asthma	Asthma	Interventional studies	Ann Allergy Asthma Immunol. 2018 Apr120(4):395-400.e1	ANNALS OF ALLERGY, ASTHMA & IMMUNOLOGY	NA	NA	24	Follow up	10.1016/j.anai.2018.02.006
Results of an asthma education program delivered via telemedicine in rural schools	Perry TT, Halterman JS, Brown RH, Luo C, Randle SM, Hunter CR, Rettiganti M.	United States of America	North America	Article	2018	Asthma	Asthma	Interventional studies	Ann Allergy Asthma Immunol. 2018 Apr120(4):401-408	ANNALS OF ALLERGY, ASTHMA & IMMUNOLOGY	NA	NA	29	Trearment	10.1016/j.anai.2018.02.013
Rostral Anterior Cingulate Cortex Morphology Predicts Treatment Response to Internet-Based Cognitive Behavioral Therapy for Depression	Webb CA, Olson EA, Killgore WDS, Pizzagalli DA, Rauch SL, Rosso IM.	United States of America	North America	Article	2018	Major depressive disorder	Mental disorders	Interventional studies	Biol Psychiatry Cogn Neurosci Neuroimaging. 2018 Mar3(3):255-262	BIOLOGICAL PSYCHIATRY: COGNITIVE NEUROSCIENCE AND NEUROIMAGING	NA	NA	0	Trearment	10.1016/j.bpsc.2017.08.005
Monthly virtual asthma care at least as efficacious as routine visits	Morton RW.	United Kingdom	Europe	Comment/editorial/letter	2018	Asthma	Asthma	Review/meta-analysis	J Pediatr. 2018 May196:324-327	JOURNAL OF PEDIATRICS	NA	NA	1	Follow up	10.1016/j.jpeds.2018.02.056
Telepsychiatry in Asperger's syndrome	Clarke CS.	Ireland	Europe	Article	2018	Autism spectrum disorders	Neurodevelopmental diseases or disorders	Case report/ case series	Ir J Psychol Med. 2018 Dec35(4):325-328	IRISH JOURNAL OF PSYCHOLOGICAL MEDICINE	NA	NA	2	Trearment	10.1017/ipm.2017.19
Using video-based telemedicine to reduce hypoglycemia fear in parents of young children with type 1 diabetes: It's feasible and acceptable	Marker A.M., Noser A.E., Calkins A.K., Maliszewski G., Clements M.A., Patton S.R.	United States of America	North America	Conference abstract	2018	Diabetes mellitus type I	Metabolic diseases	Interventional studies	Journal of Diabetes Science and Technology (2018) 12:2 (A48)	JOURNAL OF DIABETES SCIENCE AND TECHNOLOGY	NA	NA	13	Trearment	10.1089/dia.2019.0244

Moderated online social therapy for depression relapse prevention in young people: pilot study of a 'next generation' online intervention	Rice S, Gleeson J, Davey C, Hetrick S, Parker A, Lederman R, Wadley G, Murray G, Herrman H, Chambers R, Russon P, Miles C, D'Alfonso S, Thurley M, Chinnery G, Gilbertson T, Eleftheriadis D, Barlow E, Cagliarini D, Toh JW, McAlpine S, Koval P, Bendall S, Jansen JE, Hamilton M, McGorry P, Alvarez-Jimenez M.	Australia	Oceania	Article	2018	Major depressive disorder	Mental disorders	Interventional studies	Early Interv Psychiatry. 2018 Aug12(4):613-625	EARLY INTERVENTION PSYCHIATRY	NA	NA	50	Trearment	10.1111/eip.12354
Televisits to Partially Substitute for Clinic Visits Are Feasible and Well Accepted by Tech-Savvy Patients With T1DM and Their Families: A Prospective Pilot Study	Tonyushkina KN, Cobb V, Moskovitz A, Allen HF.	United States of America	North America	Comment/editorial/letter	2018	Diabetes mellitus type I	Metabolic diseases	Interventional studies	J Diabetes Sci Technol. 2018 Sep12(5):1084-1085	JOURNAL OF DIABETES SCIENCE AND TECHNOLOGY	NA	NA	4	Follow up	10.1177/1932296818775438
Novel Bluetooth-Enabled Tubeless Insulin Pump: A User Experience Design Approach for a Connected Digital Diabetes Management Platform	Pillalamarri SS, Huyett LM, Abdel-Malek A.	United States of America	North America	Article	2018	Diabetes mellitus type I	Metabolic diseases	Study protocol/Pilot study	J Diabetes Sci Technol. 2018 Nov12(6):1132-1142	JOURNAL OF DIABETES SCIENCE AND TECHNOLOGY	NA	NA	6	Trearment	10.1177/1932296818804802
Impact of a digital health intervention on asthma resource utilization	Merchant, R; Szefler, SJ; Bender, BG; Tuffli, M; Barrett, MA; Gondalia, R; Kaye, L; Van Sickle, D; Stempel, DA	United States of America	North America	Article	2018	Asthma	Asthma	Interventional studies	World Allergy Organ J. 2018 Dec 3;11(1):28	WORLD ALLERGY ORGANIZATION JOURNAL	NA	NA	30	Trearment	10.1186/s40413-018-0209-0
Advancement Of Teledentistry At The University Of Rochester's Eastman Institute For Oral Health	Kopycka-Kedzierawski DT, McLaren SW, Billings RJ.	United States of America	North America	Article	2018	Caries of permanent teeth	Dental caries	Observational studies	Health Aff (Millwood). 2018 Dec;37(12):1960-1966	HEALTH AFFAIRS	NA	NA	15	Diagnosis	10.1377/hlthaff.2018.05102
AID-GM: An Advanced System Supporting Continuous Monitoring of T1DM Patients	Salvi, E; Sacchi, L; Made, A; Calcaterra, V; Bellazzi, R; Larizza, C	Italy	Europe	Article	2018	Diabetes mellitus type I	Metabolic diseases	Study protocol/Pilot study	Stud Health Technol Inform. 2018;247:616-620	STUDIES IN HEALTH TECHNOLOGY AND INFORMATICS	NA	NA	1	Follow up	10.3233/978-1-61499-852-5-616
Testing the Efficacy of a Smartphone Application in Improving Medication Adherence, Among Children with ADHD	Weisman O, Schonherz Y, Harel T, Efron M, Elazar M, Gothelf D.	Israel	Asia	Article	2018	Conduct disorder	Neurodevelopmental diseases or disorders	Interventional studies	Isr J Psychiatry. 201855(2):59-63	ISRAEL JOURNAL OF PSYCHIATRY	NA	NA	0	Trearment	NA

Effectiveness of Telemonitoring Intervention in Children and Adolescents with Asthma: A Systematic Review and Meta-Analysis	Jung, Y., Kim, J., Park, D.A.	South Korea	Asia	Article	2018	Asthma	Asthma	Review/meta-analysis	Journal of Korean Academy of Nursing 48(4), pp. 389-406	JOURNAL OF KOREAN ACADEMY OF FUNDAMENTALS OF NURSING	0,984	NURSING	3	Follow up	10.4040/jkan.2018.48.4.389
Effect of the School-Based Telemedicine Enhanced Asthma Management (SB-TEAM) Program on Asthma Morbidity: A Randomized Clinical Trial	Halterman JS, Fagnano M, Tajon RS, Tremblay P, Wang H, Butz A, Perry TT, McConnochie KM.	United States of America	North America	Article	2018	Asthma	Asthma	Interventional studies	JAMA Pediatr. 2018 Mar 5172(3):e174938	JAMA PEDIATRICS	16,193	PEDIATRICS	76	Follow up	10.1001/jamapediatrics.2017.4938
Effect of the School-Based Telemedicine Enhanced Asthma Management (SB-TEAM) Program on Asthma Morbidity A Randomized Clinical Trial	Halterman, JS; Fagnano, M; Tajon, RS; Tremblay, P; Wang, HY; Butz, A; Perry, TT; McConnochie, KM	United States of America	North America	Article	2018	Asthma	Asthma	Interventional studies	JAMA Pediatr. 2018 Mar 5;172(3):e174938	JAMA PEDIATRICS	16,193	PEDIATRICS	76	Trearment	10.1001/jamapediatrics.2017.4938
Asthma Management in the Era of Smart-Medicine: Devices, Gadgets, Apps and Telemedicine	Katwa U, Rivera E.	United States of America	North America	Article	2018	Asthma	Asthma	Review/meta-analysis	Indian J Pediatr. 2018 Sep85(9):757-762	INDIAN JOURNAL OF PEDIATRICS	1,967	PEDIATRICS	20	Diagnosis & Treatment/Follow-up	10.1007/s12098-018-2611-6
ISPAD Clinical Practice Consensus Guidelines 2018: Diabetes technologies	Sherr, JL; Tauschmann, M; Battelino, T; de Bock, M; Forlenza, G; Roman, R; Hood, KK; Maahs, DM	United States of America	North America	Book Chapter	2018	Diabetes mellitus type I	Metabolic diseases	Observational studies	Pediatr Diabetes. 2018 Oct;19 Suppl 27:302-325.	PEDIATRIC DIABETES	4,866	PEDIATRICS	0	Follow up	10.1111/pedi.12731
Applying Interactive Mobile health to Asthma Care in Teens (AIM2ACT): Development and design of a randomized controlled trial	Fedele DA, McConville A, Graham Thomas J, McQuaid EL, Janicke DM, Turner EM, Moon J, Abu-Hasan M.	United States of America	North America	Article	2018	Asthma	Asthma	Interventional studies	Contemp Clin Trials. 2018 Jan64:230-237	CONTEMPORARY CLINICAL TRIALS	2,226	PHARMACOLOGY & PHARMACY	18	Trearment	0.1016/j.cct.2017.09.007
Experiences of an internet-based support and coaching model for adolescents and young adults with ADHD and autism spectrum disorder -a qualitative study	Sehlin H, Hedman Ahlström B, Andersson G, Wentz E.	Sweden	Europe	Article	2018	Autism spectrum disorders	Neurodevelopmental diseases or disorders	Interventional studies	BMC Psychiatry. 2018 Jan 1818(1):15	BMC PSYCHIATRY	3,63	PSYCHIATRY	13	Trearment	10.1186/s12888-018-1599-9
Working From Home: An Initial Pilot Examination of Videoconferencing-Based Cognitive Behavioral Therapy for Anxious Youth Delivered to the Home Setting	Carpenter AL, Pincus DB, Furr JM, Comer JS.	United States of America	North America	Article	2018	Anxiety disorders	Mental disorders	Interventional studies	Behav Ther. 2018 Nov49(6):917-930	BEHAVIOR THERAPY	4,183	PSYCHIATRY	25	Trearment	10.1016/j.beth.2018.01.007
Electronic Screen Media Use in Youth With Autism Spectrum Disorder	Gwynette MF, Sidhu SS, Ceranoglu TA.	United States of America	North America	Article	2018	Autism spectrum disorders	Neurodevelopmental diseases or disorders	Review/meta-analysis	Child Adolesc Psychiatr Clin N Am. 2018 Apr27(2):203-219	CHILD AND ADOLESCENT PSYCHIATRIC CLINICS OF NORTH AMERICA	2,41	PSYCHIATRY	19	Diagnosis & Treatment/Follow-up	10.1016/j.chc.2017.11.013

Telebehavioral Health Interventions: Diverse Populations and Settings	Myers K., Chronis-Tuscano A.M.	United States of America	North America	Conference abstract	2018	Major depressive disorder	Mental disorders	Guidelines/consensus paper	Journal of the American Academy of Child and Adolescent Psychiatry (2018) 57:10 Supplement (S8-S9)	JOURNAL OF THE AMERICAN ACADEMY OF CHILD AND ADOLESCENT PSYCHIATRY	8,829	PSYCHIATRY	0	Diagnosis & Treatment/Follow-up	10.1016/j.jaac.2018.07.038
Lessons Learned Across Pediatric Psychology Projects Using Home-Based Telebehavioral Health	Nelson E.-L., Patton S., Davis A., Wright S., Thompson N., Yadrich D., Smith C.	United States of America	North America	Conference abstract	2018	Diabetes mellitus type I	Metabolic diseases	Guidelines/consensus paper	Journal of the American Academy of Child and Adolescent Psychiatry (2018) 57:10 Supplement (S9-S10)	JOURNAL OF THE AMERICAN ACADEMY OF CHILD AND ADOLESCENT PSYCHIATRY	8,829	PSYCHIATRY	0	Diagnosis & Treatment/Follow-up	10.1016/j.jaac.2018.07.041
Intercontinental telehealth coaching of therapists to improve verbalizations by children with autism	Barkaia A., Stokes T.F., Mikiashvili T.	United States of America	North America	Article	2018	Autism spectrum disorders	Neurodevelopmental diseases or disorders	Case report/ case series	J Appl Behav Anal. 2017 Jul;50(3):582-589	JOURNAL OF APPLIED BEHAVIOR ANALYSIS	3,695	PSYCHOLOGY, CLINICAL	25	Trearment	10.1002/jaba.391
Delivering solid treatments on shaky ground: Feasibility study of an online therapy for child anxiety in the aftermath of a natural disaster	Stasiak K, Merry SN, Frampton C, Moor S.	New Zeland	Oceania	Article	2018	Anxiety disorders	Mental disorders	Interventional studies	Psychother Res. 2018 Jul28(4):643-653	PSYCHOTHERAPY RESEARCH	3,768	PSYCHOLOGY, CLINICAL	13	Trearment	10.1080/10503307.2016.1244617
Feasibility of Parent Training via Telehealth for Children with Autism Spectrum Disorder and Disruptive Behavior: A Demonstration Pilot	Bearss K, Burrell TL, Challa SA, Postorino V, Gillespie SE, Crooks C, Scahill L.	United States of America	North America	Article	2018	Autism spectrum disorders	Neurodevelopmental diseases or disorders	Interventional studies	J Autism Dev Disord. 2018 Apr48(4):1020-1030	JOURNAL OF AUTISM AND DEVELOPMENTAL DISORDERS	4,291	PSYCHOLOGY, DEVELOPMENTAL	51	Trearment	10.1007/s10803-017-3363-2
Early Identification of ASD Through Telemedicine: Potential Value for Underserved Populations	Juárez AP, Weitlauf AS, Nicholson A, Pasternak A, Broderick N, Hine J, Stainbrook JA, Warren Z.	United States of America	North America	Article	2018	Autism spectrum disorders	Neurodevelopmental diseases or disorders	Interventional studies	J Autism Dev Disord. 2018 Aug48(8):2601-2610	JOURNAL OF AUTISM AND DEVELOPMENTAL DISORDERS	4,291	PSYCHOLOGY, DEVELOPMENTAL	44	Diagnosis	10.1007/s10803-018-3524-y
Religious versus Conventional Internet-based Cognitive Behavioral Therapy for Depression	Tulbure BT, Andersson G, Sălăgean N, Pearce M, Koenig HG.	Romania	Europe	Article	2018	Major depressive disorder	Mental disorders	Interventional studies	J Relig Health. 2018 Oct57(5):1634-1648	JOURNAL OF RELIGION & HEALTH	1,898	PUBLIC, ENVIRONMENTAL & OCCUPATIONAL HEALTH	7	Trearment	10.1007/s10943-017-0503-0
Development of an eHealth Program for Parents of Adolescents With Type 1 Diabetes	Whittemore R, Zincavage RM, Jaser SS, Grey M, Coleman JL, Collett D, Delvy R, Basile Ibrahim B, Marceau LD.	United States of America	North America	Article	2018	Diabetes mellitus type I	Metabolic diseases	Interventional studies	Diabetes Educ. 2018 Feb44(1):72-82	DIABETES EDUCATOR	2,14	PUBLIC, ENVIRONMENTAL & OCCUPATIONAL HEALTH	7	Follow up	10.1177/0145721717748606

Diabetes and TelecommunicationS (DATES) study to support self-management for people with type 2 diabetes: a randomized controlled trial	Al-Ozairi E, Ridge K, Taghadom E, de Zoysa N, Tucker C, Stewart K, Stahl D, Ismail K.	Kuwait	Asia	Article	2018	Diabetes mellitus type I	Metabolic diseases	Interventional studies	BMC Public Health. 2018 Nov 1218(1):1249	BMC PUBLIC HEALTH	3,295	PUBLIC, ENVIRONMENTAL & OCCUPATIONAL HEALTH	5	Follow up	10.1186/s12889-018-6136-8
EFFECTS OF TELEHEALTH BY ALLIED HEALTH PROFESSIONALS AND NURSES IN RURAL AND REMOTE AREAS: A SYSTEMATIC REVIEW AND META-ANALYSIS	Speyer R.,Denman D.,Wilkes-Gillan S.,Chen Y.-W.,Bogaardt H.,Kim J.-H.,Heckathorn D.-E.,Cordier R.	Australia	Oceania	Article	2018	Asthma	Asthma	Review/meta-analysis	J Rehabil Med. 2018 Feb 28;50(3):225-235	JOURNAL OF REHABILITATION MEDICINE	2,912	REHABILITATION	69	Diagnosis & Treatment/Follow-up	10.2340/16501977-2297
Occupation-Based Coaching by Means of Telehealth for Families of Young Children With Autism Spectrum Disorder	Little LM, Pope E, Wallisch A, Dunn W.	United States of America	North America	Article	2018	Autism spectrum disorders	Neurodevelopmental diseases or disorders	Interventional studies	Am J Occup Ther. 2018 Mar/Apr72(2):7202205020p1-7202205020p7	AMERICAN JOURNAL OF OCCUPATIONAL THERAPY	2,246	REHABILITATION	29	Trearment	10.5014/ajot.2018.024786
Novel methods for device and adherence monitoring in asthma	Bonini, M; Usmani, OS	United Kingdom	Europe	Article	2018	Asthma	Asthma	Review/meta-analysis	Curr Opin Pulm Med. 2018 Jan;24(1):63-69	CURRENT OPINION IN PULMONARY MEDICINE	3,155	RESPIRATORY SYSTEM	19	Follow up	10.1097/MCP.0000000000000439
The nationwide program of allergic disease prevention as an implementation of GARD guidelines in Poland	Raciborski, F; Samolinski, B; Krzych-Falta, E; Grabczewska, A; Furman, F; Bieszczad, M; Morkisz, P; Witkowska, P; Bousquet, J; Gujski, M	Poland	Europe	Article	2018	Asthma	Asthma	Interventional studies	J Thorac Dis. 2018 Sep;10(9):5595-5604	JOURNAL OF THORACIC DISEASE	2,895	RESPIRATORY SYSTEM	2	Diagnosis	10.21037/jtd.2018.08.97
Telemonitoring in asthma control: a randomized controlled trial	Nemanic T, Sarc I, Skrgat S, Flezar M, Cukjati I, Marc Malovrh M.	Slovenia	Europe	Article	2019	Asthma	Asthma	Interventional studies	J Asthma. 2019 Jul56(7):782-790	JOURNAL OF ASTHMA	2,515	ALLERGY	5	Follow up	10.1080/02770903.2018.1493599
Comparing the outcomes of gold-standard dental examinations with photographic screening by mid-level dental providers	Park JS, Kruger E, Nicholls W, Estai M, Winters J, Tennant M.	Australia	Oceania	Article	2019	Caries of deciduous teeth	Dental caries	Interventional studies	Clin Oral Investig. 2019 May23(5):2383-2387	CLINICAL ORAL INVESTIGATIONS	3,573	DENTISTRY, ORAL SURGERY & MEDICINE	2	Diagnosis	10.1007/s00784-018-2700-y
Online Care Versus In-Person Care for Improving Quality of Life in Psoriasis: A Randomized Controlled Equivalency Trial	Armstrong, AW; Ford, AR; Chambers, CJ; Maverakis, E; Dunnick, CA; Chren, MM; Gelfand, JM; Gibbons, CM; Gibbons, BM; Lane, CJ	United States of America	North America	Article	2019	Psoriasis	Skin diseases	Interventional studies	J Invest Dermatol. 2019 May;139(5):1037-1044	JOURNAL OF INVESTIGATIVE DERMATOLOGY	8,551	DERMATOLOGY	18	Follow up	10.1016/j.jid.2018.09.039

Democratizing type 1 diabetes (T1D) knowledge in rural and underserved communities: Project echo T1D	Cuttriss N., Walker Walker A., Maahs D., Haller M., Anez-Zabala C., Yabut Yabut K., Hu H., Filipp S.	United States of America	North America	Conference abstract	2019	Diabetes mellitus type I	Metabolic diseases	Interventional studies	Diabetes Technology and Therapeutics (2019) 21 Supplement 1 (A118-A119)	DIABETES TECHNOLOGY & THERAPEUTICS	6,118	ENDOCRINOLOGY & METABOLISM	0	Trearment	10.1089/dia.2019.2525.abstracts
Cost-effectiveness of Shared Telemedicine Appointments in Young Adults With T1D: CoYoT1 Trial	Wan W, Nathan AG, Skandari MR, Zarei P, Reid MW, Raymond JK, Huang ES.	United States of America	North America	Article	2019	Diabetes mellitus type I	Metabolic diseases	Interventional studies	Diabetes Care. 2019 Aug42(8):1589-1592	DIABETES CARE	19,112	ENDOCRINOLOGY & METABOLISM	4	Trearment	10.2337/dc19-0363
Has Technology Improved Diabetes Management in Relation to Age, Gender, and Ethnicity?	Eiland L., Thangavelu T., Drincic A.	United States of America	North America	Article	2019	Diabetes mellitus type I	Metabolic diseases	Review/meta-analysis	Current Diabetes Reports (2019) 19:11	CURRENT DIABETES REPORTS	4,813	ENDOCRINOLOGY & METABOLISM	2	Diagnosis & Treatment/Follow-up	10.1007/s11892-019-1231-5
Improving Glycemic Control in Adults and Children With Type 1 Diabetes With the Use of Smartphone-Based Mobile Applications: A Systematic Review	Sun C, Malcolm JC, Wong B, Shorr R, Doyle MA.	Canada	North America	Article	2019	Diabetes mellitus type I	Metabolic diseases	Review/meta-analysis	Can J Diabetes. 2019 Feb43(1):51-58.e3	CANADIAN JOURNAL OF DIABETES	4,19	ENDOCRINOLOGY & METABOLISM	21	Trearment	10.1016/j.jcjd.2018.03.010
Mobile Health and Technology Usage by Patients in the Diabetes, Nutrition, and Weight Management Clinic at an Urban Academic Medical Center	Stockman MC, Modzelewski K, Steenkamp D.	United States of America	North America	Article	2019	Diabetes mellitus type I	Metabolic diseases	Observational studies	Diabetes Technol Ther. 2019 Jul21(7):400-405	DIABETES TECHNOLOGY & THERAPEUTICS	6,118	ENDOCRINOLOGY & METABOLISM	4	Trearment	10.1089/dia.2018.0369
The Emerging Role of Telemedicine and Mobile Health Technologies in Improving Diabetes Care	Garg, SK; Parkin, CG	United States of America	North America	Comment/editorial/letter	2019	Diabetes mellitus type I	Metabolic diseases	Review/meta-analysis	Diabetes Technol Ther. 2019 Jun;21(S2):S21-S23	DIABETES TECHNOLOGY & THERAPEUTICS	6,118	ENDOCRINOLOGY & METABOLISM	7	Trearment	10.1089/dia.2019.0090
Mobile-based insulin dose adjustment for type 2 diabetes in community and rural populations: study protocol for a pilot randomized controlled trial	Menon A., Gray L., Fatehi F., Bird D., Darssan D., Karunanithi M., Russell A.	Australia	Oceania	Article	2019	Diabetes mellitus type I	Metabolic diseases	Interventional studies	Therapeutic Advances in Endocrinology and Metabolism (2019) 10	THERAPEUTIC ADVANCES IN ENDOCRINOLOGY AND METABOLISM	3,536	ENDOCRINOLOGY & METABOLISM	3	Trearment	10.1177/2042018819836647
Development and Evaluation the Satisfaction of Using an Oral Health Survey Mobile Application	Detsomboonrat, P; Pisarnturakit, PP	Thailand	Asia	Article	2019	Caries of deciduous teeth	Dental caries	Observational studies	Telemed J E Health. 2019 Jan;25(1):55-59	TELEMEDICINE AND E-HEALTH	3,536	HEALTH CARE SCIENCES & SERVICES	2	Diagnosis	10.1089/tmj.2017.0288
Development and Evaluation of a Mobile Oral Health Application for Preschoolers	Campos, LFXA; Cavalcante, JP; Machado, DP; Maral, E; Silva, PGD; Rolim, JPML	Brazil	South America	Article	2019	Caries of deciduous teeth	Dental caries	Interventional studies	Telemed J E Health. 2019 Jun;25(6):492-498.	TELEMEDICINE AND E-HEALTH	3,536	HEALTH CARE SCIENCES & SERVICES	6	Prevention	10.1089/tmj.2018.0034

Access to Dermatological Care with an Innovative Online Model for Psoriasis Management: Results from a Randomized Controlled Trial	Ford, AR; Gibbons, CM; Torres, J; Kornmehl, HA; Singh, S; Young, PM; Chambers, CJ; Maverakis, E; Dunnick, CA; Armstrong, AW	United States of America	North America	Article	2019	Psoriasis	Skin diseases	Interventional studies	Telemed J E Health. 2019 Jul;25(7):619-627	TELEMEDICIN E AND E-HEALTH	3,536	HEALTH CARE SCIENCES & SERVICES	12	Follow up	10.1089/tmj.2018.0160
Reduced medical spending associated with increased use of a remote diabetes management program and lower mean blood glucose values	Whaley CM, Bollyky JB, Lu W, Painter S, Schneider J, Zhao Z, He X, Johnson J, Meadows ES.	United States of America	North America	Article	2019	Diabetes mellitus type I	Metabolic diseases	Observational studies	J Med Econ. 2019 Sep22(9):869-877	JOURNAL OF MEDICAL ECONOMICS	2,448	HEALTH CARE SCIENCES & SERVICES	16	Follow up	10.1080/13696998.2019.1609483
Clinical Effectiveness of Telemedicine in Diabetes Mellitus: A Meta-Analysis of 42 Randomized Controlled Trials	Tchero H, Kangambega P, Briatte C, Brunet-Houdard S, Retali GR, Rusch E.	United Kingdom	Europe	Article	2019	Diabetes mellitus type I	Metabolic diseases	Review/meta-analysis	Telemed J E Health. 2019 Jul25(7):569-583	TELEMEDICIN E AND E-HEALTH	3,536	HEALTH CARE SCIENCES & SERVICES	68	Diagnosis & Treatment/Follow-up	10.1089/tmj.2018.0128
Vortex Whistle and Smart Phone Application for Peak Flow Recordings in Asthmatic Children: A Feasibility Study	Mikalsen IB, Nassehi D, Øymar K.	Norway	Europe	Article	2019	Asthma	Asthma	Interventional studies	Telemed J E Health. 2019 Nov25(11):1077-1082	TELEMEDICIN E AND E-HEALTH	3,536	HEALTH CARE SCIENCES & SERVICES	8	Trearment	10.1089/tmj.2018.0270
The design and development of MyT1DHero: A mobile app for adolescents with type 1 diabetes and their parents	Holtz BE, Murray KM, Hershey DD, Richman J, Dunneback JK, Vyas A, Wood MA.	United States of America	North America	Article	2019	Diabetes mellitus type I	Metabolic diseases	Interventional studies	J Telemed Telecare. 2019 Apr25(3):172-180	JOURNAL OF TELEMEDICIN E AND TELECARE	6,184	HEALTH CARE SCIENCES & SERVICES	8	Follow up	10.1177/1357633X17745470
The Michigan child collaborative care program: Building a telepsychiatry consultation service	Marcus S., Malas N., Dopp R., Quigley J., Kramer A.C., Tengelitsch E., Patel P.D.	United States of America	North America	Conference abstract	2019	Anxiety disorders	Mental disorders	Observational studies	Psychiatric Services (2019) 70:9 (849-852)	PSYCHIATRIC SERVICES	3,084	HEALTH POLICY & SERVICES	6	Diagnosis & Treatment/Follow-up	10.1176/appi.ps.201800151
Assessing the Need for Mobile Health (mHealth) in Monitoring the Diabetic Lower Extremity	Wallace D, Perry J, Yu J, Mehta J, Hunter P, Cross KM.	Canada	North America	Article	2019	Diabetes mellitus type I	Metabolic diseases	Observational studies	JMIR Mhealth Uhealth. 2019 Apr 167(4):e11879	JMIR mHEALTH AND uHEALTH	4,773	MEDICAL INFORMATICS	4	Follow up	10.2196/11879
Effective Engagement of Adolescent Asthma Patients With Mobile Health-Supporting Medication Adherence	Kosse RC, Bouvy ML, Belitser SV, de Vries TW, van der Wal PS, Koster ES.	Netherlands	Europe	Article	2019	Asthma	Asthma	Interventional studies	JMIR Mhealth Uhealth. 2019 Mar 277(3):e12411	JMIR mHEALTH AND uHEALTH	4,773	MEDICAL INFORMATICS	21	Trearment	10.2196/12411
Use of Electronic Health and Its Impact on Doctor-Visiting Decisions Among People With Diabetes: Cross-Sectional Study	Hansen AH, Claudi T, Årsand E.	Norway	Europe	Article	2019	Diabetes mellitus type I	Metabolic diseases	Observational studies	J Med Internet Res. 2019 Apr 2621(4):e13678	JOURNAL OF MEDICAL INTERNET RESEARCH	5,428	MEDICAL INFORMATICS	2	Follow up	10.2196/13678

Hospital Utilization Among Rural Children Served by Pediatric Neurology Telemedicine Clinics	Dayal, P; Chang, CH; Benko, WS; Pollock, BH; Crossen, SS; Kissee, J; Ulmer, AM; Hoch, JS; Warner, L; Marcin, JP	United States of America	North America	Article	2019	Epilepsy	Brain disorders	Observational studies	JAMA Netw Open. 2019 Aug 2;2(8):e199364	JAMA NETWORK OPEN	8,485	MEDICINE, GENERAL & INTERNAL	12	Trearment	10.1001/jamanetworkopen.2019.9364
Responsive Asthma Care for Teens (ReACT): development protocol for an adaptive mobile health intervention for adolescents with asthma	Cushing CC, Fedele DA, Patton SR, McQuaid EL, Smyth JM, Prabhakaran S, Gierer S, Koskela-Staples N, Ortega A, Fleming KK, Nezu AM.	United States of America	North America	Article	2019	Asthma	Asthma	Study protocol/Pilot study	BMJ Open. 2019 Aug 209(8):e030029	BMJ OPEN	2,692	MEDICINE, GENERAL & INTERNAL	3	Follow up	10.1136/bmjopen-2019-030029
Clinical and economic outcomes of remotely delivered cognitive behaviour therapy versus treatment as usual for repeat unscheduled care users with severe health anxiety: a multicentre randomised controlled trial	Morriss R, Patel S, Malins S, Guo B, Higton F, James M, Wu M, Brown P, Boycott N, Kaylor-Hughes C, Morris M, Rowley E, Simpson J, Smart D, Stubley M, Kai J, Tyrer H.	multicenter	More than one continent	Article	2019	Anxiety disorders	Mental disorders	Interventional studies	BMC Med. 2019 Jan 2317(1):16	BMC MEDICINE	8,775	MEDICINE, GENERAL & INTERNAL	24	Diagnosis & Treatment/Follow-up	10.1186/s12916-019-1253-5
Study protocol for a randomized controlled trial to test for preventive effects of diabetic foot ulceration by telemedicine that includes sensor-equipped insoles combined with photo documentation	Ming A, Walter I, Alhajjar A, Leuckert M, Mertens PR.	Germany	Europe	Article	2019	Diabetes mellitus type I	Metabolic diseases	Study protocol/Pilot study	Trials. 2019 Aug 2220(1):521	TRIALS	2,279	MEDICINE, RESEARCH & EXPERIMENTAL	11	Trearment	10.1186/s13063-019-3623-x
The majority of skin lesions in pediatric primary care attention could be managed by Teledermatology	Giavina Bianchi M, Santos AP, Cordioli E.	United States of America	North America	Article	2019	Atopic dermatitis	Skin diseases	Interventional studies	PLoS One. 2019 Dec 214(12):e0225479	PLoS ONE	3,24	MULTIDISCIPLINARY SCIENCES	17	Diagnosis & Treatment/Follow-up	10.1371/journal.pone.0225479
Appointment completion in pediatric neurology telemedicine clinics serving underserved patients	Dayal, P; Chang, CH; Benko, WS; Ulmer, AM; Crossen, SS; Pollock, BH; Hoch, JS; Kissee, JL; Warner, L; Marcin, JP	United States of America	North America	Article	2019	Epilepsy	Brain disorders	Observational studies	Neurol Clin Pract. 2019 Aug;9(4):314-321	NEUROLOGY CLINICAL PRACTICE	NA	NA	10	Follow up	10.1212/CPJ.0000000000000649
A validation study of questionnaire towards mobile based health applications in uncontrolled diabetic population of India (South)	Ashok Kumar M, Shanmugasundaram P.	United Kingdom	Europe	Article	2019	Diabetes mellitus type I	Metabolic diseases	Interventional studies	Diabetes Metab Syndr. 2019 May-Jun13(3):2106-2110	DIABETOLOGY & METABOLIC SYNDROME	NA	NA	0	Follow up	10.1016/j.dsx.2019.04.037
What Is the Impact of Innovative Electronic Health Interventions in Improving Treatment Adherence in Asthma? The Pediatric Perspective	Licari A, Ferrante G, Marseglia Md GL, Corsello Md G, La Grutta S.	Italy	Europe	Article	2019	Asthma	Asthma	Review/meta-analysis	J Allergy Clin Immunol Pract. 2019 Nov-Dec7(8):2574-2579	JOURNAL OF ALLERGY AND CLINICAL IMMUNOLOGY: IN PRACTICE	NA	NA	6	Follow up	10.1016/j.jaip.2019.08.008

School-Based Telemedicine for Asthma Management	Perry TT, Turner JH.	United States of America	North America	Article	2019	Asthma	Asthma	Review/meta-analysis	J Allergy Clin Immunol Pract. 2019 Nov-Dec7(8):2524-2532	JOURNAL OF ALLERGY AND CLINICAL IMMUNOLOGY: IN PRACTICE	NA	NA	13	Trearment	10.1016/j.jaip.2019.08.009
The Good, the Bad, and the Unknown of Telemedicine in Asthma and Allergy Practice	Chen, A; Rehman, N; Portnoy, J	United States of America	North America	Comment/editorial/letter	2019	Asthma	Asthma	Review/meta-analysis	J Allergy Clin Immunol Pract. 2019 Nov-Dec;7(8):2580-2582	JOURNAL OF ALLERGY AND CLINICAL IMMUNOLOGY: IN PRACTICE	NA	NA	7	Follow up	10.1016/j.jaip.2019.08.017
Providing neurodiagnostic care through telemedicine	Padilla E.	United States of America	North America	Conference abstract	2019	Epilepsy	Brain disorders	Case report/ case series	Neurodiagnostic Journal (2019) 59:4 (241)	NEURODIAGNOSTIC JOURNAL	NA	NA	0	Diagnosis & Treatment/Follow-up	10.1080/21646821.2019.1682868
Telehealth and autism: Are telehealth language assessments reliable and feasible for children with autism?	Sutherland R, Trembath D, Hodge MA, Rose V, Roberts J.	Australia	Oceania	Article	2019	Autism spectrum disorders	Neurodevelopmental diseases or disorders	Review/meta-analysis	Int J Lang Commun Disord. 2019 Mar54(2):281-291	INTERNATIONAL JOURNAL OF LANGUAGE & COMMUNICATION DISORDERS	NA	NA	19	Trearment	10.1111/1460-6984.12440
The WHAAM Application: a Tool to Support the Evidence-Based Practice in the Functional Behaviour Assessment	Merlo G, Chiazzese G, Sanches-Ferreira M, Chifari A, Seta L, McGee C, Mirisola A, Giammusso I.	multicenter	More than one continent	Article	2019	Attention-deficit/hyperactivity disorder	Neurodevelopmental diseases or disorders	Interventional studies	J Innov Health Inform. 2018 Jun 2925(2):63-70	JOURNAL OF INNOVATION IN HEALTH INFORMATICS	NA	NA	5	Trearment	10.14236/jhi.v25i2.919
Impact Of eHealth in Allergic Diseases and Allergic Patients	Alvarez-Perea, A; Sanchez-Garcia, S; Cano, M; Antolin-Amerigo, D; Tsilochristou, O; Stukus, DR	Spain	Europe	Article	2019	Asthma	Asthma	Review/meta-analysis	J Investig Allergol Clin Immunol. 2019;29(2):94-102	JOURNAL OF INVESTIGATIONAL ALLERGOLOGY AND CLINICAL IMMUNOLOGY	NA	NA	15	Follow up	10.18176/jiaci.0354
Patient-Generated Health Data Integration and Advanced Analytics for Diabetes Management: The AID-GM Platform	Salvi E, Bosoni P, Tibollo V, Kruijver L, Calcaterra V, Sacchi L, Bellazzi R, Larizza C.	Italy	Europe	Article	2019	Diabetes mellitus type I	Metabolic diseases	Study protocol/Pilot study	Sensors (Basel). 2019 Dec 2420(1):128	SENSORS (BASEL)	NA	NA	6	Trearment	10.3390/s20010128
Telemedicine System Model to Help Children with Autism Spectrum Disorders	Seepold, R; Lebedev, G; Madrid, NM	multicenter	More than one continent	Article	2019	Autism spectrum disorders	Neurodevelopmental diseases or disorders	Study protocol/Pilot study	AIP Conference Proceedings 2140,020064	AIP CONFERENCE PROCEEDINGS	NA	NA	2	Trearment	https://doi.org/10.1063/1.5121989

Capability of implementing telehealth in a pediatric neurology population with high health disparities	Camayd-Munoz C., Miller E., Rollins J., Sewell K., Wenger J., Jonas R., Douglass L.	United States of America	North America	Conference abstract	2019	Epilepsy	Brain disorders	Observational studies	Annals of Neurology (2017) 82 Supplement 21 (S284). Date of Publication: 1 Oct 2017	ANNALS OF NEUROLOGY	10,422	NEUROSCIENCES	0	Trearment	https://doi.org/10.1111/dmcn.15256
Self-management of sick days in young people with type 1 diabetes enhanced by phone support: A qualitative study	Farrell K, Brunero S, Holmes-Walker DJ, Griffiths R, Salamonson Y.	United States of America	North America	Article	2019	Diabetes mellitus type I	Metabolic diseases	Interventional studies	Contemp Nurse. 2019 Apr-Jun55(2-3):171-184	CONTEMPORARY NURSE	1,787	NURSING	0	Follow up	10.1080/10376178.2019.1640620
A randomized controlled trial of a mobile application-assisted nurse-led model used to improve treatment outcomes in children with asthma	Lv S, Ye X, Wang Z, Xia W, Qi Y, Wang W, Chen Y, Cai X, Qian X.	China	Asia	Article	2019	Asthma	Asthma	Interventional studies	J Adv Nurs. 2019 Nov75(11):3058-3067	JOURNAL OF ADVANCED NURSING	3,187	NURSING	13	Trearment	10.1111/jan.14143
Characteristics of type 1 diabetes patients using continuous glucose monitoring systems and development of retinopathy	Khouri C.A., Ooms A., Thangmathesvaran L., Khouri P., Szirth B.	United States of America	North America	Conference abstract	2019	Diabetes mellitus type I	Metabolic diseases	Observational studies	Investigative Ophthalmology and Visual Science (2019) 60:9	INVESTIGATIVE OPHTHALMOLOGY AND VISUAL SCIENCE	4,799	OPHTHALMOLOGY	0	Follow up	NA
Assessment of a tele-diagnosis experimentation of autism spectrum disorders	Doyen C., Desailly E., Goupil V., Kaye K.	France	Europe	Conference abstract	2019	Autism spectrum disorders	Neurodevelopmental diseases or disorders	Observational studies	Developmental Medicine and Child Neurology (2019) 61 Supplement 2 (24)	DEVELOPMENTAL MEDICINE AND CHILD NEUROLOGY	1,7	PEDIATRICS	0	Diagnosis & Treatment/Follow-up	10.1111/dmcn.14244
Successful CGM initiation early in the course of T1D results in persistence of use	Prahalad P., Ith A., Scheinker D., Pageler N., Hood K., Addala A., Freeman A., Chmielewski A., Conrad B., Geels E., Leverenz J., Peterson K., Maahs D.	United States of America	North America	Conference abstract	2019	Diabetes mellitus type I	Metabolic diseases	Interventional studies	Pediatric Diabetes (2019) 20 Supplement 28 (14)	PEDIATRIC DIABETES	4,866	PEDIATRICS	0	Trearment	10.1111/pedi.12923
Association of a School-Based, Asthma-Focused Telehealth Program with Emergency Department Visits among Children Enrolled in South Carolina Medicaid	Bian J., Cristaldi K.K., Summer A.P., Su Z., Marsden J., Mauldin P.D., McElligott J.T.	United States of America	North America	Article	2019	Asthma	Asthma	Observational studies	JAMA Pediatrics (2019) 173:11 (1041-1048)	JAMA PEDIATRICS	16,193	PEDIATRICS	18	Trearment	10.1001/jamapediatrics.2019.3073
How does video-counseling work under real-life conditions? Results from the VIDIKI study, a multicenter, controled study evaluating the impact of monthly video consultations for children with type 1 diabetes using	Frielitz F.-S., Mueller-Godeffroy E., Eisemann N., Lange K., Doerdelmann J., Erdem A., Menrath I., Bokelmann J., Krasmann M., Kaczmarczyk P., Bertram B., Hiort O.,	Germany	Europe	Conference abstract	2019	Diabetes mellitus type I	Metabolic diseases	Interventional studies	Pediatric Diabetes (2019) 20 Supplement 28 (178)	PEDIATRIC DIABETES	4,866	PEDIATRICS	0	Follow up	10.1111/pedi.12924

a continuous glucose monitoring system	Katalinic A., Von Sengbusch S.														
Contributions and limits of Integrated Telemedicine in diabetes care and education program: Perceptions of families from Chilean remote rural area	Pelicand J., Silva M.B., Vergara S.B., Soto B.Ó.	Chile	South America	Conference abstract	2019	Diabetes mellitus type I	Metabolic diseases	Observational studies	Pediatric Diabetes (2019) 20 Supplement 28 (176)	PEDIATRIC DIABETES	4,866	PEDIATRICS	0	Trearment	10.1111/pedi.12924
Effect of Telehealth System on Glycemic Control in Children and Adolescents with Type 1 Diabetes	Döğer E, Bozbulut R, Soysal Acar AŞ, Ercan Ş, Kılınç Uğurlu A, Akbaş ED, Bideci A, Çamurdan O, Cinaz P.	Turkey	Europe	Article	2019	Diabetes mellitus type I	Metabolic diseases	Interventional studies	J Clin Res Pediatr Endocrinol. 2019 Feb 2011(1):70-75	JOURNAL OF CLINICAL RESEARCH IN PEDIATRIC ENDOCRINOLOGY	1,933	PEDIATRICS	20	Trearment	10.4274/jcrpe.galenos.2018.2018.0017
Evaluation of a mobile health intervention to support asthma self-management and adherence in the pharmacy	Kosse RC, Bouvy ML, de Vries TW, Koster ES.	Netherlands	Europe	Article	2019	Asthma	Asthma	Interventional studies	Int J Clin Pharm. 2019 Apr41(2):452-459	INTERNATIONAL JOURNAL OF CLINICAL PHARMACY	2,054	PHARMACOLOGY & PHARMACY	6	Trearment	0.1007/s11096-019-00798-3
IoT based assistive companion for hypersensitive individuals (ACHI) with autism spectrum disorder	Khullar V, Singh HP, Bala M.	India	Asia	Article	2019	Autism spectrum disorders	Neurodevelopmental diseases or disorders	Interventional studies	Asian J Psychiatr. 2019 Dec46:92-102	ASIAN JOURNAL OF PSYCHIATRY	3,543	PSYCHIATRY	9	Diagnosis & Treatment/Follow-up	10.1016/j.ajp.2019.09.030
Remote assessment of disease and relapse in major depressive disorder (RADAR-MDD): a multi-centre prospective cohort study protocol	Matcham F, Barattieri di San Pietro C, Bulgari V, de Girolamo G, Dobson R, Eriksson H, Folarin AA, Haro JM, Kerz M, Lamers F, Li Q, Manyakov NV, Mohr DC, Myin-Germeys I, Narayan V, Bwjh P, Ranjan Y, Rashid Z, Rintala A, Siddi S, Simblett SK, Wykes T, Hotopf M	multicenter	More than one continent	Article	2019	Major depressive disorder	Mental disorders	Interventional studies	BMC Psychiatry. 2019 Feb 18;19(1):72	BMC PSYCHIATRY	3,63	PSYCHIATRY	28	Follow up	10.1186/s12888-019-2049-z
Teens Using Screens for Help: Impact of Suicidal Ideation, Anxiety, and Depression Levels on Youth Preferences for Telemental Health Resources	Toscos, T; Coupe, A; Flanagan, M; Drouin, M; Carpenter, M; Reining, L; Roebuck, A; Mirro, MJ	United States of America	North America	Article	2019	Anxiety disorders	Mental disorders	Observational studies	JMIR Ment Health. 2019;6(6):e13230. Published 2019 Jun 21	JMIR MENTAL HEALTH	4,388	PSYCHIATRY	7	Diagnosis	10.2196/13230
Telemedicine and autism spectrum disorder in children and adolescents: Theoretical and practical guide	Doyen C., Goupil V., Desailly E., Oreve M.J., Kaye K.	France	Europe	Article	2019	Autism spectrum disorders	Neurodevelopmental diseases or disorders	Interventional studies	Annales Medico-Psychologiques (2019) 177:7 (702-709)	ANNALES MEDICO-PSYCHOLOGIQUES	0,38	PSYCHIATRY	0	Diagnosis & Treatment/Follow-up	10.1016/j.amp.2019.04.017

Out-of-home informal support important for medication adherence, diabetes distress, hemoglobin A1c among adults with type 2 diabetes	Mayberry LS, Piette JD, Lee AA, Aikens JE.	United States of America	North America	Article	2019	Diabetes mellitus type I	Metabolic diseases	Observational studies	J Behav Med. 2019 Jun42(3):493-501	JOURNAL OF BEHAVIORAL MEDICINE	2,96	PSYCHOLOGY, CLINICAL	4	Trearment	10.1007/s10865-018-0002-0
Telehealth as a Model for Providing Behaviour Analytic Interventions to Individuals with Autism Spectrum Disorder: A Systematic Review	Ferguson, J; Craig, EA; Dounavi, K	United Kingdom	Europe	Article	2019	Autism spectrum disorders	Neurodevelopmental diseases or disorders	Review/meta-analysis	J Autism Dev Disord. 2019 Feb;49(2):582-616	JOURNAL OF AUTISM AND DEVELOPMENTAL DISORDERS	4,291	PSYCHOLOGY, DEVELOPMENTAL	79	Trearment	10.1007/s10803-018-3724-5
A Randomised Controlled Trial of an Information Communication Technology Delivered Intervention for Children with Autism Spectrum Disorder Living in Regional Australia	Parsons D, Cordier R, Lee H, Falkmer T, Vaz S.	Australia	Oceania	Article	2019	Autism spectrum disorders	Neurodevelopmental diseases or disorders	Interventional studies	J Autism Dev Disord. 2019 Feb49(2):569-581	JOURNAL OF AUTISM AND DEVELOPMENTAL DISORDERS	4,291	PSYCHOLOGY, DEVELOPMENTAL	17	Prevention	10.1007/s10803-018-3734-3
Measuring the service system impact of a novel telediagnostic service program for young children with autism spectrum disorder	Stainbrook JA, Weitlauf AS, Juárez AP, Taylor JL, Hine J, Broderick N, Nicholson A, Warren Z.	United States of America	North America	Article	2019	Autism spectrum disorders	Neurodevelopmental diseases or disorders	Interventional studies	Autism. 2019 May23(4):1051-1056	AUTISM	5,689	PSYCHOLOGY, DEVELOPMENTAL	20	Diagnosis & Treatment/Follow-up	10.1177/136236131878779 7
MyDiaText™: Feasibililty and Functionality of a Text Messaging System for Youth With Type 1 Diabetes	Kaushal T, Montgomery KA, Simon R, Lord K, Dougherty J, Katz LEL, Lipman TH.	United States of America	North America	Article	2019	Diabetes mellitus type I	Metabolic diseases	Interventional studies	Diabetes Educ. 2019 Jun45(3):253-259	DIABETES EDUCATOR	2,14	PUBLIC, ENVIRONMENTAL & OCCUPATIONAL HEALTH	4	Trearment	10.1177/014572171983789 5
Home Telemedicine (CoYoT1 Clinic): A Novel Approach to Improve Psychosocial Outcomes in Young Adults With Diabetes	Bakhach M, Reid MW, Pyatak EA, Berget C, Cain C, Thomas JF, Klingensmith GJ, Raymond JK.	United States of America	North America	Article	2019	Diabetes mellitus type I	Metabolic diseases	Interventional studies	Diabetes Educ. 2019 Aug45(4):420-430	DIABETES EDUCATOR	2,14	PUBLIC, ENVIRONMENTAL & OCCUPATIONAL HEALTH	24	Trearment	10.1177/014572171985808 0
An Online Mindfulness-Based Cognitive Behavioral Therapy Intervention for Youth Diagnosed With Major Depressive Disorders: Protocol for a Randomized Controlled Trial	Ritvo, P; Daskalakis, ZJ; Tomlinson, G; Ravindran, A; Linklater, R; Chang, MK; Knyahnytska, Y; Lee, J; Alavi, N; Bai, S; Harber, L; Jain, T; Katz, J	Canada	North America	Article	2019	Major depressive disorder	Mental disorders	Study protocol/Pilot study	JMIR Res Protoc. 2019 Jul 29;8(7):e11591	JMIR RESEARCH PROTOCOLS	0,67	PUBLIC, ENVIRONMENTAL & OCCUPATIONAL HEALTH	2	Trearment	10.2196/11591
The home remote diagnostics of bronchial asthma in children with the using of telemedical system	Malinin S., Furman E., Rocheva E., Sokolovsky V., Furman G.	Russia	Europe	Conference abstract	2019	Asthma	Asthma	Observational studies	European Respiratory Journal (2019) 54 Supplement 63	EUROPEAN RESPIRATORY JOURNAL	16,671	RESPIRATORY SYSTEM	0	Diagnosis	10.1183/13993003.congress-2019.PA739

Effect of a mHealth intervention on adherence in adolescents with asthma: A randomized controlled trial	Kosse RC, Bouvy ML, de Vries TW, Koster ES.	Netherland	Europe	Article	2019	Asthma	Asthma	Interventional studies	Respir Med. 2019 Mar149:45-51	RESPIRATORY MEDICINE	2,054	RESPIRATORY SYSTEM	6	Trearment	10.1016/j.rmed.2019.02.009
Impact of eHealth on medication adherence among patients with asthma: A systematic review and meta-analysis	Jeminiwa R, Hohmann L, Qian J, Garza K, Hansen R, Fox BI.	United States of America	North America	Article	2019	Asthma	Asthma	Review/meta-analysis	Respir Med. 2019 Mar149:59-68	RESPIRATORY MEDICINE	3,415	RESPIRATORY SYSTEM	24	Trearment	10.1016/j.rmed.2019.02.011
Targeting quality of life in asthmatic children: The MyTEP pilot randomized trial	Montalbano L, Ferrante G, Cilluffo G, Gentile M, Arrigo M, La Guardia D, Allegra M, Malizia V, Gagliardo RP, Bonini M, La Grutta S.	Italy	Europe	Article	2019	Asthma	Asthma	Interventional studies	Respir Med. 2019 Jul153:14-19	RESPIRATORY MEDICINE	3,415	RESPIRATORY SYSTEM	11	Trearment	10.1016/j.rmed.2019.05.008
Improving asthma control by facilitating patient-centered care at school through telemedicine	Lin N.Y., Miller J.L., Ramsey R.R., Hommel K.A., Guilbert T.W.	United States of America	North America	Conference abstract	2019	Asthma	Asthma	Interventional studies	American Journal of Respiratory and Critical Care Medicine (2019) 199:9	AMERICAN JOURNAL OF RESPIRATORY AND CRITICAL CARE MEDICINE	21,405	RESPIRATORY SYSTEM	0	Trearment	10.1089/tmj.2017.0330
Clinical effect on asthma control using a novel digital self-management solution: a physician blinded randomized controlled crossover trial	Ljungberg, H; Carleborg, A; Nordlund, B	Sweden	Europe	Article	2019	Asthma	Asthma	Interventional studies	Eur Respir J. 2019 Nov 14;54(5):1900983	EUROPEAN RESPIRATORY JOURNAL	16,671	RESPIRATORY SYSTEM	6	Trearment	10.1183/13993003.00983-2019
INNER CITY ASTHMA TELE-MEDICINE DURING COVID PANDEMIC	Kwong K.	United States of America	North America	Conference abstract	2020	Asthma	Asthma	Observational studies	Annals of Allergy, Asthma and Immunology (2020) 125:5 Supplement (S11)	ANNALS OF ALLERGY, ASTHMA & IMMUNOLOGY	6,347	ALLERGY	0	Follow up	10.1016/j.anai.2020.08.054
DELIVERING EVIDENCE-BASED ASTHMA EDUCATION IN THE WORLD OF TELEMEDICINE: A QUALITY IMPROVEMENT PROJECT	Tison K.	United States of America	North America	Conference abstract	2020	Asthma	Asthma	Observational studies	Annals of Allergy, Asthma and Immunology (2020) 125:5 Supplement (S43-S44)	ANNALS OF ALLERGY, ASTHMA & IMMUNOLOGY	6,347	ALLERGY	0	Follow up	10.1016/j.anai.2020.08.148
Telehealth Enhanced Asthma Management (TEAM)	Turner J., Berlinski A., Chervinskiy S., Simmons L., Jones S., Carroll J., Harwell S., Perry T.	United States of America	North America	Conference abstract	2020	Asthma	Asthma	Interventional studies	Journal of Allergy and Clinical Immunology (2020) 145:2 Supplement (AB162)	JOURNAL OF ALLERGY AND CLINICAL IMMUNOLOGY	10,793	ALLERGY	0	Trearment	10.1016/j.jaci.2019.12.427

New Concepts and Technological Resources in Patient Education and Asthma Self-Management	Poowuttikul, P; Seth, D	United States of America	North America	Article	2020	Asthma	Asthma	Review/meta-analysis	Clin Rev Allergy Immunol. 2020 Aug;59(1):19-37	CLINICAL REVIEWS IN ALLERGY & IMMUNOLOGY	8,667	ALLERGY	10	Follow up	10.1007/s12016-020-08782-w
COVID-19: Pandemic Contingency Planning for the Allergy and Immunology Clinic	Shaker, MS; Oppenheimer, J; Grayson, M; Stukus, D; Hartog, N; Hsieh, EWY; Rider, N; Dutmer, CM; Vander Leek, TK; Kim, H; Chan, ES; Mack, D; Ellis, AK; Lang, D; Lieberman, J; Fleischer, D; Golden, DBK; Wallace, D; Portnoy, J; Mosnaim, G; Greenhawt, M	United States of America	North America	Article	2020	Asthma	Asthma	Review/meta-analysis	Journal of Allergy and Clinical Immunology: In Practice 2020 8: 5 (1477-1488.e5)	JOURNAL OF ALLERGY AND CLINICAL IMMUNOLOGY: IN PRACTICE	8,861	ALLERGY	192	Follow up	10.1016/j.jaip.2020.03.012
Impact of COVID-19 on Pediatric Asthma: Practice Adjustments and Disease Burden	Papadopoulos NG, Custovic A, Deschildre A, Mathioudakis AG, Phipatanakul W, Wong G, Xepapadaki P, Agache I, Bacharier L, Bonini M, Castro-Rodriguez JA, Chen Z, Craig T, Ducharme FM, El-Sayed ZA, Feleszko W, Fiocchi A, Garcia-Marcos L, Gern JE, Goh A, Gómez RM, Hamelmann EH, Hedlin G, Hossny EM, Jartti T, Kalayci O, Kaplan A, Konradsen J, Kuna P, Lau S, Le Souef P, Lemanske RF, Mäkelä MJ, Morais-Almeida M, Murray C, Nagaraju K, Namazova-Baranova L, Garcia AN, Yusuf OM, Pitrez PMC, Pohunek P, Pozo Beltrán CF, Roberts GC, Valiulis A, Zar HJ	United Kingdom	Europe	Article	2020	Asthma	Asthma	Guidelines/consensus paper	J Allergy Clin Immunol Pract. 2020 Sep;8(8):2592-2599.e3	JOURNAL OF ALLERGY AND CLINICAL IMMUNOLOGY: IN PRACTICE	8,861	ALLERGY	62	Diagnosis & Treatment/Follow-up	10.1016/j.jaip.2020.06.001
Bridging the healthcare gap: Building the case for epilepsy virtual clinics in the current healthcare environment	Lavin, B; Dormond, C; Scantlebury, MH; Frouin, PY; Brodie, MJ	multicenter	More than one continent	Article	2020	Epilepsy	Brain disorders	Review/meta-analysis	Epilepsy Behav. 2020 Oct;111:107262	EPILEPSY AND BEHAVIOR	2,937	BEHAVIORAL SCIENCES	8	Follow up	10.1016/j.yebeh.2020.107262

Telehealth in pediatric epilepsy care: A rapid transition during the COVID-19 pandemic	Sattar S., Kuperman R.	United States of America	North America	Article	2020	Epilepsy	Brain disorders	Guidelines/consensus paper	Epilepsy and Behavior (2020) 111	EPILEPSY AND BEHAVIOR	2,937	BEHAVIORAL SCIENCES	13	Diagnosis & Treatment/Follow-up	10.1016/j.yebeh.2020.107282
SARS-CoV-2-related rapid reorganization of an epilepsy outpatient clinic from personal appointments to telemedicine services: A German single-center experience	Willems L.M., Balcik Y., Noda A.H., Siebenbrodt K., Leimeister S., McCoy J., Kienitz R., Kiyose M., Reinecke R., Schäfer J.-H., Zöllner J.P., Bauer S., Rosenow F., Strzelczyk A.	Germany	Europe	Article	2020	Epilepsy	Brain disorders	Observational studies	Epilepsy and Behavior (2020) 112	EPILEPSY AND BEHAVIOR	2,937	BEHAVIORAL SCIENCES	14	Diagnosis & Treatment/Follow-up	10.1016/j.yebeh.2020.107483
Telemedicine, drug-resistant epilepsy, and ketogenic dietary therapies: A patient survey of a pediatric remote-care program during the COVID-19 pandemic	Semprino, M; Fasulo, L; Fortini, S; Molina, CIM; Gonzalez, L; Ramos, PA; Martinez, C; Caraballo, R	Argentina	South America	Article	2020	Epilepsy	Brain disorders	Interventional studies	Epilepsy & behavior : E&B vol. 112 (2020): 107493.	EPILEPSY AND BEHAVIOR	2,937	BEHAVIORAL SCIENCES	14	Trearment	10.1016/j.yebeh.2020.107493
Impact of COVID-19 pandemic on pediatric patients with epilepsy - The caregiver perspective	Trivisano, M; Specchio, N; Pietrafusa, N; Calabrese, C; Ferretti, A; Ricci, R; Renzetti, T; Raponi, M; Vigevano, F	Italy	Europe	Article	2020	Epilepsy	Brain disorders	Observational studies	Epilepsy Behav. 2020 Dec;113:107527	EPILEPSY AND BEHAVIOR	2,937	BEHAVIORAL SCIENCES	8	Follow up	10.1016/j.yebeh.2020.107527
What should we ask patients with epilepsy on telemedicine during the COVID-19 crisis? A checklist for clinicians	Kuroda, N	United States of America	North America	Comment/editorial/letter	2020	Epilepsy	Brain disorders	Guidelines/consensus paper	Epilepsy Behav. 2020 Oct;111:107184	EPILEPSY AND BEHAVIOR	2,937	BEHAVIORAL SCIENCES	3	Follow up	10.1016/j.yebeh.2020.107184
Telemedicine and epilepsy: A patient satisfaction survey of a pediatric remote care program	Fortini S., Espeche A., Caraballo R.	Argentina	South America	Article	2020	Epilepsy	Brain disorders	Observational studies	Epilepsy Research (2020) 165	EPILEPSY RESEARCH	3,045	CLINICAL NEUROLOGY	9	Trearment	10.1016/j.eplepsyres.2020.106370
A Pilot Randomized Controlled Trial to Assess the Impact of Motivational Interviewing on Initiating Behavioral Therapy for Migraine	Minen MT, Sahyoun G, Gopal A, Levitan V, Pirraglia E, Simon NM, Halpern A.	United States of America	North America	Article	2020	Migraine	Brain disorders	Interventional studies	Headache. 2020 Feb60(2):441-456	HEADACHE	5,887	CLINICAL NEUROLOGY	6	Trearment	10.1111/head.13738
Biofeedback Treatment App for Pediatric Migraine: Development and Usability Study	Stubberud A, Tronvik E, Olsen A, Gravdahl G, Linde M.	Norway	Europe	Article	2020	Migraine	Brain disorders	Observational studies	Headache. 2020 May60(5):889-901	HEADACHE	5,887	CLINICAL NEUROLOGY	6	Trearment	10.1111/head.13772

Migraine Care in the Era of COVID-19: Clinical Pearls and Plea to Insurers	Szperka, CL; Ailani, J; Barmherzig, R; Klein, BC; Minen, MT; Singh, RBH; Shapiro, RE	United States of America	North America	Article	2020	Migraine	Brain disorders	Review/meta-analysis	Headache : official publication of the American association for the study of headache , 2020, Vol.60(5), p.833-842	HEADACHE	5,887	CLINICAL NEUROLOGY	62	Trearment	10.1111/head.13810
Employee and Employer Benefits From a Migraine Management Program: Disease Outcomes and Cost Analysis	Schaetz L, Rimner T, Pathak P, Fang J, Chandrasekhar D, Mueller J, Sandor PS, Gantenbein AR.	Switzerland	Europe	Article	2020	Migraine	Brain disorders	Interventional studies	Headache. 2020 Oct60(9):1947-1960	HEADACHE	5,887	CLINICAL NEUROLOGY	4	Diagnosis & Treatment/Follow-up	10.1111/head.13933
Migraine Care in the Era of COVID-19: Clinical Pearls and Plea to Insurers	Szperka, CL; Ailani, J; Barmherzig, R; Klein, BC; Minen, MT; Singh, RBH; Shapiro, RE	United States of America	North America	Article	2020	Migraine	Brain disorders	Review/meta-analysis	Headache. 2020 May;60(5):833-842	HEADACHE	5,887	CLINICAL NEUROLOGY	62	Trearment	10.1111/head.13810
Teledentistry as a novel pathway to improve dental health in school children: a research protocol for a randomised controlled trial	Estai M, Kanagasingam Y, Mehdizadeh M, Vignarajan J, Norman R, Huang B, Spallek H, Irving M, Arora A, Kruger E, Tennant M.	Australia	Oceania	Article	2020	Caries of deciduous teeth	Dental caries	Study protocol/Pilot study	BMC Oral Health. 2020 Jan 1420(1):11	BMC ORAL HEALTH	2,757	DENTISTRY, ORAL SURGERY & MEDICINE	18	Prevention	10.1186/s12903-019-0992-1
Telemedicine approach for psoriasis management, time for application? A systematic review of published studies	Dahy A., El-Qushayri A.E., Mahmoud A.R., Al-kelany T.A., Salman S.	Egypt	Africa	Article	2020	Psoriasis	Skin diseases	Review/meta-analysis	Dermatologic Therapy (2020) 33:6	DERMATOLOGIC THERAPY	2,851	DERMATOLOGY	2	Diagnosis & Treatment/Follow-up	10.1111/dth.13908
Combining teledermatology with nonphysician members of the health care team to address access and compliance barriers in pediatric atopic dermatitis: A needs assessment	Kourosh AS, Schneider L, Hawryluk EB, Tong LX, Rea CJ, Kvedar J.	United States of America	North America	Comment/editorial/letter	2020	Atopic dermatitis	Skin diseases	Review/meta-analysis	J Am Acad Dermatol. 2020 Jul83(1):237-239	JOURNAL OF THE AMERICAN ACADEMY OF DERMATOLOGY	11,527	DERMATOLOGY	2	Diagnosis	10.1016/j.jaad.2019.12.012
Cell-phone acne' epidemic during the COVID-19 pandemic	Singh M, Pawar M, Maheswari A, Bothra A, Khunger N.	India	Asia	Conference abstract	2020	Acne vulgaris	Skin diseases	Case report/ case series	Clin Exp Dermatol. 2020 Oct45(7):903-905	CLINICAL AND EXPERIMENTAL DERMATOLOGY	3,47	DERMATOLOGY	1	Trearment	10.1111/ced.14360
Changes in access to educational and healthcare services for individuals with intellectual and developmental disabilities during COVID-19 restrictions	Jeste S., Hyde C., Distefano C., Halladay A., Ray S., Porath M., Wilson R.B., Thurm A.	United States of America	North America	Article	2020	Autism spectrum disorders	Neurodevelopmental diseases or disorders	Observational studies	Journal of Intellectual Disability Research (2020) 64:11 (825-833)	JOURNAL OF INTELLECTUAL DISABILITY RESEARCH	2,424	EDUCATION, SPECIAL	57	Follow up	10.1111/jir.12776

Caregiver Training Via Telehealth on Behavioral Procedures: A Systematic Review	Unholz-Bowden, EmilySend mail to Unholz-Bowden E.;McComas, Jennifer J.;McMaster, Kristen L.;Girtler, Shawn N.;Kolb, Rebecca L.;Shipchandler, Alefyah	United States of America	North America	Article	2020	Autism spectrum disorders	Neurodevelopmental diseases or disorders	Review/meta-analysis	Journal of Behavioral Education, 2020 29(2), pp. 246-281	JOURNAL OF BEHAVIORAL EDUCATION	2,396	EDUCATION, SPECIAL	21	Trearment	doi.org/10.1007/s10864-020-09381-7
A Systematic and Quality Review of Parent-Implemented Language and Communication Interventions Conducted via Telepractice	Akemoglu, Y; Muharib, R; Meadan, H	United States of America	North America	Article	2020	Autism spectrum disorders	Neurodevelopmental diseases or disorders	Review/meta-analysis	Journal of Behavioral Education 29(2), pp. 282-316	JOURNAL OF BEHAVIORAL EDUCATION	2,396	EDUCATION, SPECIAL	11	Trearment	https://doi.org/10.1007/s10864-019-09356-3
Evaluation of telephone and virtual visits for routine pediatric diabetes care during the COVID-19 pandemic	Fung A., Irvine M., Ayub A., Ziabakhsh S., Amed S., Hursh B.E.	Canada	North America	Article	2020	Diabetes mellitus type I	Metabolic diseases	Observational studies	Journal of Clinical and Translational Endocrinology (2020) 22	JOURNAL OF CLINICAL AND TRANSLATIONAL ENDOCRINOLOGY	0,51	ENDOCRINOLOGY & METABOLISM	11	Follow up	10.1016/j.jcte.2020.100238
Top 10 Tips for Successfully Implementing a Diabetes Telehealth Program	Crossen, S; Raymond, J; Neinstein, A	United States of America	North America	Article	2020	Diabetes mellitus type I	Metabolic diseases	Review/meta-analysis	Diabetes Technol Ther. 2020 Dec;22(12):920-928	DIABETES TECHNOLOGY & THERAPEUTICS	6,118	ENDOCRINOLOGY & METABOLISM	20	Trearment	10.1089/dia.2020.0042
Integrated telemedicine in diabetes care and education program: Whay do Chilean families living in remote rural area think about it?	Pelicand J., Silva M.B., Vergara S.B., Soto B.O.	Chile	South America	Conference abstract	2020	Diabetes mellitus type I	Metabolic diseases	Observational studies	Diabetes Technology and Therapeutics (2020) 22 Supplement 1 (A-30)	DIABETES TECHNOLOGY & THERAPEUTICS	6,118	ENDOCRINOLOGY & METABOLISM	0	Trearment	10.1089/dia.2020.2525.abstracts
Monthly, structured video consultations for children with type 1 diabetes have a positive effect on metabolic control, diabetes burden and treatment satisfaction. results from the vidiki study, a multicenter controlled trial	Von Sengbusch S., Eisemann N., Lange K., Doerdelmann J., Lemke S., Hiort O., Katalinic A., Frielitz F.-S.	Germany	Europe	Conference abstract	2020	Diabetes mellitus type I	Metabolic diseases	Interventional studies	Diabetes Technology and Therapeutics (2020) 22 Supplement 1 (A-31)	DIABETES TECHNOLOGY & THERAPEUTICS	6,118	ENDOCRINOLOGY & METABOLISM	0	Trearment	10.1089/dia.2020.2525.abstracts
Glycemic control in type 1 diabetes mellitus and COVID-19 lockdown: What comes after a "quarantine"?	Ceconi V, Barbi E, Tornese G.	Italy	Europe	Article	2020	Diabetes mellitus type I	Metabolic diseases	Review/meta-analysis	J Diabetes. 2020 Dec12(12):946-948	JOURNAL OF DIABETES	4,006	ENDOCRINOLOGY & METABOLISM	11	Trearment	10.1111/1753-0407.13110
Lower use of technology in youth with type 1 diabetes in locations distant from specialty care	Cobry E.C., Reznick-Lipina T., Pyle L., Slover R.H., Thomas J.F., Alonso G.T., Wadwa R.P.	United States of America	North America	Conference abstract	2020	Diabetes mellitus type I	Metabolic diseases	Observational studies	Diabetes (2020) 69 Supplement 1. Date of Publication: 1 Jun 2020	DIABETES	9,461	ENDOCRINOLOGY & METABOLISM	0	Trearment	10.2337/db20-1321-P

Caring for children and adolescents with type 1 diabetes mellitus: Italian Society for Pediatric Endocrinology and Diabetology (ISPED) statements during COVID-19 pandemia	d'Annunzio G, Maffeis C, Cherubini V, Rabbone I, Scaramuzza A, Schiaffini R, Minuto N, Piccolo G, Maghnie M.	Italy	Europe	Conference abstract	2020	Diabetes mellitus type I	Metabolic diseases	Guidelines/consensus paper	Diabetes Res Clin Pract. 2020 Oct168:108372	DIABETES RESEARCH AND CLINICAL PRACTICE	5,602	ENDOCRINOLOGY & METABOLISM	18	Follow up	10.1016/j.diabres.2020.108372
Caring for a child with type 1 diabetes during COVID-19 lockdown in a developing country: Challenges and parents' perspectives on the use of telemedicine	Odeh R, Gharaibeh L, Daher A, Kussad S, Alassaf A.	Jordan	Asia	Article	2020	Diabetes mellitus type I	Metabolic diseases	Observational studies	Diabetes Res Clin Pract. 2020 Oct168:108393	DIABETES RESEARCH AND CLINICAL PRACTICE	5,602	ENDOCRINOLOGY & METABOLISM	15	Follow up	10.1016/j.diabres.2020.108393
An Intervention to Reduce Hypoglycemia Fear in Parents of Young Kids with Type 1 Diabetes Through Video-Based Telemedicine (REDCHiP): Trial Design, Feasibility, and Acceptability	Marker AM, Monzon AD, Nelson EL, Clements MA, Patton SR.	United States of America	North America	Article	2020	Diabetes mellitus type I	Metabolic diseases	Interventional studies	Diabetes Technol Ther. 2020 Jan22(1):25-33	DIABETES TECHNOLOGY & THERAPEUTICS	6,111	ENDOCRINOLOGY & METABOLISM	12	Trearment	10.1089/dia.2019.0244
A Systematic Review of Pediatric Telediabetes Service Models	De Guzman KR, Snoswell CL, Taylor ML, Senanayake B, Haydon HM, Batch JA, Smith AC, Caffery LJ.	United States of America	North America	Article	2020	Diabetes mellitus type I	Metabolic diseases	Review/meta-analysis	Diabetes Technol Ther. 2020 Aug22(8):623-638	DIABETES TECHNOLOGY & THERAPEUTICS	6,118	ENDOCRINOLOGY & METABOLISM	21	Follow up	10.1089/dia.2019.0489
Type 1 Doing Well: Pilot Feasibility and Acceptability Study of a Strengths-Based mHealth App for Parents of Adolescents with Type 1 Diabetes	Hilliard ME, Cao VT, Eshtehardi SS, Minard CG, Saber R, Thompson D, Karaviti LP, Anderson BJ.	United States of America	North America	Article	2020	Diabetes mellitus type I	Metabolic diseases	Interventional studies	Diabetes Technol Ther. 2020 Nov22(11):835-845	DIABETES TECHNOLOGY & THERAPEUTICS	6,118	ENDOCRINOLOGY & METABOLISM	2	Trearment	10.1089/dia.2020.0048
Managing New-Onset Type 1 Diabetes During the COVID-19 Pandemic: Challenges and Opportunities	Garg SK, Rodbard D, Hirsch IB, Forlenza GP.	United States of America	North America	Article	2020	Diabetes mellitus type I	Metabolic diseases	Case report/ case series	Diabetes Technol Ther. 2020 Jun22(6):431-439	DIABETES TECHNOLOGY & THERAPEUTICS	6,111	ENDOCRINOLOGY & METABOLISM	83	Diagnosis & Treatment/Follow-up	10.1089/dia.2020.0161
A virtual clinic for the management of diabetes-type 1: study protocol for a randomised wait-list controlled clinical trial	Nerpin E, Toft E, Fischier J, Lindholm-Olinder A, Leksell J.	United Kingdom	Europe	Article	2020	Diabetes mellitus type I	Metabolic diseases	Interventional studies	BMC Endocr Disord. 2020 Sep 520(1):137	BMC ENDOCRINE DISORDERS	2,763	ENDOCRINOLOGY & METABOLISM	2	Follow up	10.1186/s12902-020-00615-3
Improving access and communication through telehealth for pediatric type 1 diabetes	Rasbach L.E., Freemark M., Purrington V., Griffis M., Annas A., Page L.C., Hall R., Benjamin R.	United States of America	North America	Conference abstract	2020	Diabetes mellitus type I	Metabolic diseases	Interventional studies	Diabetes (2020) 69 Supplement 1	DIABETES	9,461	ENDOCRINOLOGY & METABOLISM	3	Trearment	10.2337/db20-1317-P

Glycemic Control Improvement in Italian Children and Adolescents With Type 1 Diabetes Followed Through Telemedicine During Lockdown Due to the COVID-19 Pandemic	Predieri B, Leo F, Candia F, Lucaccioni L, Madeo SF, Pugliese M, Vivaccia V, Bruzzi P, Iughetti L.	Italy	Europe	Article	2020	Diabetes mellitus type I	Metabolic diseases	Observational studies	Front Endocrinol (Lausanne). 2020 Dec 711:595735	FRONTIERS IN ENDOCRINOLOGY (LAUSANNE)	5,555	ENDOCRINOLOGY & METABOLISM	38	Trearment	10.3389/fendo.2020.595735
Telehealth Increases Access to Care for Children Dealing with Suicidality, Depression, and Anxiety in Rural Emergency Departments	Fairchild, RM; Ferng-Kuo, SF; Rahmouni, H; Hardesty, D	United States of America	North America	Article	2020	Major depressive disorder	Mental disorders	Interventional studies	Telemedicine and e-Health.Nov 2020.1353-1362.	TELEMEDICINE AND E-HEALTH	3,536	HEALTH CARE SCIENCES & SERVICES	16	Trearment	10.1089/tmj.2019.0253
Effectiveness of theory-based digital self-management interventions for improving depression, anxiety, fatigue and self-efficacy in people with neurological disorders: A systematic review and meta-analysis	Lau, SCL; Bhattacharjya, S; Fong, MWM; Nicol, GE; Lenze, EJ; Baum, C; Hardi, A; Wong, AWK	United States of America	North America	Article	2020	Anxiety disorders	Mental disorders	Review/meta-analysis	J Telemed Telecare. 2020 Sep 20:1357633X20955122	JOURNAL OF TELEMEDICINE AND TELECARE	6,184	HEALTH CARE SCIENCES & SERVICES	3	Trearment	10.1177/1357633X20955122
Teledentistry: Increasing utilisation of oral-health services for children in rural areas	Surdu S, Langelier M.	United States of America	North America	Article	2020	Caries of deciduous teeth	Dental caries	Observational studies	J Telemed Telecare. 2020 Oct 18:1357633X20965425	JOURNAL OF TELEMEDICINE AND TELECARE	6,184	HEALTH CARE SCIENCES & SERVICES	0	Follow up	10.1177/1357633X20965425
Barriers, access and management of paediatric epilepsy with telehealth	Gali K., Joshi S., Hueneke S., Katzenbach A., Radecki L., Calabrese T., Fletcher L., Trandafir C., Wilson C., Goyal M., Wusthoff C.J., Le Pichon J.-B., Corvalan R., Golson A., Hardy J., Smith M., Cook E., Bonkowsky J.L.	United States of America	North America	Article	2020	Epilepsy	Brain disorders	Observational studies	Journal of telemedicine and telecare (2020) (1357633X20969531)	JOURNAL OF TELEMEDICINE AND TELECARE	6,184	HEALTH CARE SCIENCES & SERVICES	3	Diagnosis & Treatment/Follow-up	10.1177/1357633X20969531
Intelligent telehealth system to support epilepsy diagnosis	Molina E., Torres C.E.S., Salazar-Cabrera R., López D.M., Vargas-Cañas R.	Colombia	South America	Article	2020	Epilepsy	Brain disorders	Observational studies	Journal of Multidisciplinary Healthcare (2020) 13 (433-445)	JOURNAL OF MULTIDISCIPLINARY HEALTHCARE	2,404	HEALTH CARE SCIENCES & SERVICES	3	Diagnosis	10.2147/JMDH.S247878
Precision telemedicine through crowdsourced machine learning: Testing variability of crowd workers for video-based autism feature recognition	Washington P., Leblanc E., Dunlap K., Penev Y., Kline A., Paskov K., Sun M.W., Chrisman B., Stockham N., Varma M., Voss C., Haber N., Wall D.P.	United States of America	North America	Article	2020	Autism spectrum disorders	Neurodevelopmental diseases or disorders	Interventional studies	Journal of Personalized Medicine (2020) 10:3 (1-13)	JOURNAL OF PERSONALIZED MEDICINE	4,945	HEALTH CARE SCIENCES & SERVICES	0	Diagnosis	10.3390/jpm10030086

Reliability of mobile phone teledentistry in dental diagnosis and treatment planning in mixed dentition	AlShaya MS, Assery MK, Pani SC.	Saudi Arabia	Asia	Article	2020	Caries of deciduous teeth	Dental caries	Interventional studies	J Telemed Telecare. 2020 Jan-Feb26(1-2):45-52	JOURNAL OF TELEMEDICIN E AND TELECARE	6,184	HEALTH CARE SCIENCES & SERVICES	22	Diagnosis	10.1177/1357633X18793767
Home-based video visits for pediatric patients with poorly controlled type 1 diabetes	Crossen S, Glaser N, Sauers-Ford H, Chen S, Tran V, Marcin J.	United States of America	North America	Article	2020	Diabetes mellitus type I	Metabolic diseases	Interventional studies	J Telemed Telecare. 2020 Jul26(6):349-355	JOURNAL OF TELEMEDICIN E AND TELECARE	6,184	HEALTH CARE SCIENCES & SERVICES	1	Diagnosis & Treatment/Follow-up	10.1177/1357633X19828173
Response to 'Home-based video visits for pediatric patients with poorly controlled type 1 diabetes'	Crossen, S; Glaser, N; Sauers-Ford, H; Chen, S; Tran, V; Marcin, J	United States of America	North America	Article	2020	Diabetes mellitus type I	Metabolic diseases	Interventional studies	J Telemed Telecare. 2020 Jul;26(6):381	JOURNAL OF TELEMEDICIN E AND TELECARE	6,184	HEALTH CARE SCIENCES & SERVICES	1	Follow up	10.1177/1357633X19861217
A markov transition model for estimating the impact of pediatric asthma medication adherence on healthcare utilization and cost	Brinton D., Simpson A., Simpson K., Andrews A.	United States of America	North America	Conference abstract	2020	Asthma	Asthma	Observational studies	Health Services Research (2020) 55:SUPPL 1 (88-89)	HEALTH SERVICES RESEARCH	3,402	HEALTH POLICY & SERVICES	0	Follow up	10.1111/1475-6773.13454
Racial Differences in the Effectiveness of a Multifactorial Telehealth Intervention to Slow Diabetic Kidney Disease	Kobe EA, Diamantidis CJ, Bosworth HB, Davenport CA, Oakes M, Alexopoulos AS, Pendergast J, Patel UD, Crowley MJ.	United States of America	North America	Article	2020	Diabetes mellitus type I	Metabolic diseases	Interventional studies	Med Care. 2020 Nov58(11):968-973	MEDICAL CARE	2,983	HEALTH POLICY & SERVICES	2	Follow up	10.1097/MLR.0000000000001387
Effectiveness of Mobile Health Interventions on Diabetes and Obesity Treatment and Management: Systematic Review of Systematic Reviews	Wang, YF; Min, J; Khuri, J; Xue, H; Xie, B; Kaminsky, LA; Cheskin, LJ	United States of America	North America	Article	2020	Diabetes mellitus type I	Metabolic diseases	Review/meta-analysis	JMIR Mhealth Uhealth. 2020 Apr 28;8(4):e15400	JMIR mHEALTH AND uHEALTH	4,773	MEDICAL INFORMATICS	34	Trearment	10.2196/15400
Creating a Smartphone App for Caregivers of Children With Atopic Dermatitis With Caregivers, Health Care Professionals, and Digital Health Experts: Participatory Co-Design	Xu X, Griva K, Koh M, Lum E, Tan WS, Thng S, Car J.	Singapore	Asia	Article	2020	Atopic dermatitis	Skin diseases	Guidelines/consensus paper	JMIR Mhealth Uhealth. 2020 Oct 298(10):e16898	JMIR mHEALTH AND uHEALTH	4,773	MEDICAL INFORMATICS	5	Follow up	10.2196/16898
Superusers' Engagement in Asthma Online Communities: Asynchronous Web-Based Interview Study	De Simoni A, Shah AT, Fulton O, Parkinson J, Sheikh A, Panzarasa P, Pagliari C, Coulson NS, Griffiths CJ.	United Kingdom	Europe	Article	2020	Asthma	Asthma	Observational studies	J Med Internet Res. 2020 Jun 2322(6):e18185	JOURNAL OF MEDICAL INTERNET RESEARCH	5,428	MEDICAL INFORMATICS	4	Trearment	10.2196/18185
Video Consultations Between Patients and Clinicians in Diabetes, Cancer, and Heart Failure Services: Linguistic Ethnographic Study of	Shaw SE, Seuren LM, Wherton J, Cameron D, A'Court C, Vijayaraghavan S, Morris J, Bhattacharya S, Greenhalgh T.	United Kingdom	Europe	Article	2020	Diabetes mellitus type I	Metabolic diseases	Observational studies	J Med Internet Res. 2020 May 1122(5):e18378	JOURNAL OF MEDICAL INTERNET RESEARCH	5,428	MEDICAL INFORMATICS	42	Follow up	10.2196/18378

Video-Mediated Interaction															
Adaptive Mobile Health Intervention for Adolescents with Asthma: Iterative User-Centered Development	Fedele DA, Cushing CC, Koskela-Staples N, Patton SR, McQuaid EL, Smyth JM, Prabhakaran S, Gierer S, Nezu AM.	Canada	North America	Article	2020	Asthma	Asthma	Interventional studies	JMIR Mhealth Uhealth. 2020 May 68(5):e18400	JMIR mHEALTH AND uHEALTH	4,773	MEDICAL INFORMATICS	1	Trearment	10.2196/18400
Beneficial Features of a mHealth Asthma App for Children and Caregivers: Qualitative Study	Iio M, Miyaji Y, Yamamoto-Hanada K, Narita M, Nagata M, Ohya Y.	Japan	Asia	Article	2020	Asthma	Asthma	Observational studies	JMIR Mhealth Uhealth. 2020 Aug 248(8):e18506	JMIR mHEALTH AND uHEALTH	4,773	MEDICAL INFORMATICS	4	Trearment	10.2196/18506
mHealth app using machine learning to increase physical activity in diabetes and depression: clinical trial protocol for the DIAMANTE Study	Aguilera A, Figueroa CA, Hernandez-Ramos R, Sarkar U, Cemballi A, Gomez-Pathak L, Miramontes J, Yom-Tov E, Chakraborty B, Yan X, Xu J, Modiri A, Aggarwal J, Jay Williams J, Lyles CR.	United States of America	North America	Article	2020	Diabetes mellitus type I	Metabolic diseases	Interventional studies	BMJ Open. 2020 Aug 2010(8):e034723	BMJ OPEN	2,692	MEDICINE, GENERAL & INTERNAL	18	Prevention	10.1136/bmjopen-2019-034723
Teledentistry in dental care of children	Popovic, L; Odalovic, D; Zivkovic, D; Miladinovic, M; Lazic, Z; Duka, M; Zivkovic, M	Serbia	Europe	Article	2020	Caries of deciduous teeth	Dental caries	Review/meta-analysis	Vojnosanitetski Pregled,2020; 77(12), pp. 1323-1326	VOJNOSANITETSKI PREGLED	0,168	MEDICINE, GENERAL & INTERNAL	0	Diagnosis	10.2298/VSP180918196P
Discrepancy between Lung Function Measurements at Home and in the Hospital in Children with Asthma and CF	Gerzon, FLGR; Jobsis, Q; Bannier, MAGE; Winkens, B; Dompeling, E	Netherland	Europe	Article	2020	Asthma	Asthma	Interventional studies	J Clin Med. 2020 May 26;9(6):1617	JOURNAL OF CLINICAL MEDICINE	4,242	MEDICINE, GENERAL & INTERNAL	12	Follow up	10.3390/jcm9061617
Telehealth diagnoses of autism spectrum disorder in toddlers	Butsch M., Aites M., Cartisano T., Roy D., Jordan B., Flake E., Tolson D.	United States of America	North America	Conference abstract	2020	Autism spectrum disorders	Neurodevelopmental diseases or disorders	Observational studies	Journal of Investigative Medicine (2020) 68:1 (A162)	JOURNAL OF INVESTIGATIVE MEDICINE	2,895	MEDICINE, RESEARCH & EXPERIMENTAL	0	Diagnosis	10.1136/jim-2019-WMRC.375
Adapting home telehealth group appointment model (CoYoT1 clinic) for a low SES, publicly insured, minority young adult population with type 1 diabetes	Raymond JK, Reid MW, Fox S, Garcia JF, Miller D, Bisno D, Fogel JL, Krishnan S, Pyatak EA.	United States of America	North America	Article	2020	Diabetes mellitus type I	Metabolic diseases	Interventional studies	Contemp Clin Trials. 2020 Jan88:105896	CONTEMPORARY CLINICAL TRIALS	2,226	MEDICINE, RESEARCH & EXPERIMENTAL	4	Trearment	10.1016/j.cct.2019.105896

Use of telehealth for facilitating the diagnostic assessment of Autism Spectrum Disorder (ASD): A scoping review	Alfuraydan M, Croxall J, Hurt L, Kerr M, Brophy S.	United Kingdom	Europe	Article	2020	Autism spectrum disorders	Neurodevelopmental diseases or disorders	Review/meta-analysis	PLoS One. 2020 Jul 2315(7):e0236415	PLoS ONE	3,24	MULTIDISCIPLINARY SCIENCES	22	Diagnosis	10.1371/journal.pone.0236415
Telephone-based follow-up of children with epilepsy: Comparison of accuracy between a specialty nurse and a pediatric neurology fellow	Gulati S., Shruthi N.M., Panda P.K., Sharawat I.K., Josey M., Pandey R.M.	India	Asia	Article	2020	Epilepsy	Brain disorders	Interventional studies	Seizure (2020) 83 (98-103)	SEIZURE	NA	NA	0	Follow up	10.1016/j.seizure.2020.10.002
Teledermatology for acne patients: How to reduce face-to-face visits during COVID-19 pandemic	Villani A, Annunziata MC, Abategiovanni L, Fabbrocini G.	Italy	Europe	Comment/editorial/letter	2020	Acne vulgaris	Skin diseases	Observational studies	J Cosmet Dermatol. 2020 Aug19(8):1828	JOURNAL OF COSMETIC DERMATOLOGY	NA	NA	11	Follow up	10.1111/jocd.13519
Use and perception of telemedicine in people with type 1 diabetes during the COVID-19 pandemic-Results of a global survey	Scott SN, Fontana FY, Züger T, Laimer M, Stettler C.	Switzerland	Europe	Article	2020	Diabetes mellitus type I	Metabolic diseases	Observational studies	Endocrinol Diabetes Metab. 2020 Aug 294(1):e00180	ENDOCRINOLOGY, DIABETES & METABOLISM	NA	NA	20	Follow up	10.1002/edm2.180
Wearable Technology and How This Can Be Implemented into Clinical Practice	Greiwe, J; Nyenhuis, SM	United States of America	North America	Article	2020	Asthma	Asthma	Review/meta-analysis	Curr Allergy Asthma Rep. 2020 Jun 6;20(8):36	CURRENT ALLERGY AND ASTHMA REPORTS	NA	NA	28	Follow up	10.1007/s11882-020-00927-3
Telemedicine and emerging technologies for health care in allergy/immunology	Portnoy, JM; Pandya, A; Waller, M; Elliott, T	United States of America	North America	Article	2020	Asthma	Asthma	Review/meta-analysis	The Journal of allergy and clinical immunology vol. 145,2 (2020): 445-454.	THE JOURNAL OF ALLERGY AND CLINICAL IMMUNOLOGY	NA	NA	24	Follow up	10.1016/j.jaci.2019.12.903
Telemedical Asthma Education and Health Care Outcomes for School-Age Children: A Systematic Review	Culmer N, Smith T, Stager C, Wright A, Burgess K, Johns S, Watt M, Desch M.	United States of America	North America	Article	2020	Asthma	Asthma	Review/meta-analysis	J Allergy Clin Immunol Pract. 2020 Jun8(6):1908-1918	JOURNAL OF ALLERGY AND CLINICAL IMMUNOLOGY: IN PRACTICE	NA	NA	12	Follow up	10.1016/j.jaip.2020.02.005
The Future of Telehealth in Allergy and Immunology Training	Keswani, A; Brooks, JP; Khoury, P	United States of America	North America	Article	2020	Asthma	Asthma	Review/meta-analysis	J Allergy Clin Immunol Pract. 2020 Jul-Aug;8(7):2135-2141	JOURNAL OF ALLERGY AND CLINICAL IMMUNOLOGY: IN PRACTICE	NA	NA	8	Follow up	10.1016/j.jaip.2020.05.009
The Unexpected Risks of COVID-19 on Asthma Control in Children	Oreskovic NM, Kinane TB, Aryee E, Kuhlthau KA, Perrin JM.	United States of America	North America	Article	2020	Asthma	Asthma	Review/meta-analysis	J Allergy Clin Immunol Pract. 2020 Sep8(8):2489-2491	JOURNAL OF ALLERGY AND CLINICAL IMMUNOLOGY: IN PRACTICE	NA	NA	27	Follow up	10.1016/j.jaip.2020.05.027

Work Group Report: COVID-19: Unmasking Telemedicine	Hare, N; Bansal, P; Bajowala, SS; Abramson, SL; Chervinskiy, S; Corriel, R; Hauswirth, DW; Kakumanu, S; Mehta, R; Rashid, Q; Rupp, MR; Shih, J; Mosnaim, GS	United States of America	North America	Article	2020	Asthma	Asthma	Review/meta-analysis	J Allergy Clin Immunol Pract. 2020 Sep;8(8):2461-2473.e3	JOURNAL OF ALLERGY AND CLINICAL IMMUNOLOGY: IN PRACTICE	NA	NA	32	Follow up	10.1016/j.jaip.2020.06.038
Pediatric Asthma Health Care Utilization, Viral Testing, and Air Pollution Changes During the COVID-19 Pandemic	Taquechel K, Diwadkar AR, Sayed S, Dudley JW, Grundmeier RW, Kenyon CC, Henrickson SE, Himes BE, Hill DA.	United States of America	North America	Article	2020	Asthma	Asthma	Observational studies	J Allergy Clin Immunol Pract. 2020 Nov-Dec8(10):3378-3387.e11	JOURNAL OF ALLERGY AND CLINICAL IMMUNOLOGY: IN PRACTICE	NA	NA	47	Follow up	10.1016/j.jaip.2020.07.057
Biomedical REAl-Time Health Evaluation (BREATHE): toward an mHealth informatics platform	Bui, AAT; Hosseini, A; Rocchio, R; Jacobs, N; Ross, MK; Okelo, S; Lurmann, F; Eckel, S; Dzubur, E; Dunton, G; Gilliland, F; Sarrafzadeh, M; Habre, R	United States of America	North America	Article	2020	Asthma	Asthma	Study protocol/Pilot study	JAMIA Open. 2020 May 7;3(2):190-200	JAMIA OPEN	NA	NA	8	Follow up	10.1093/jamiaopen/ooaa011
Using a Systems Engineering Approach to Design an Interactive Mobile Health Application for Improving Asthma Self-management	Li X, Wyatt TH, Velur Rajashekaran P, Bayless AK, Odom L.	United States of America	North America	Article	2020	Asthma	Asthma	Review/meta-analysis	Comput Inform Nurs. 2020 Dec 439(4):221-228	COMPUTERS INFORMATICS NURSING	NA	NA	0	Follow up	10.1097/CIN.0000000000000691
Monthly Video-Consultation for Children With Type 1 Diabetes Using a Continuous Glucose Monitoring System: Design of ViDiKi, a Multimethod Intervention Study to Evaluate the Benefit of Telemedicine	Frielitz FS, Müller-Godeffroy E, Hübner J, Eisemann N, Dördelmann J, Menrath I, Katalinic A, Hiort O, von Sengbusch S.	Germany	Europe	Article	2020	Diabetes mellitus type I	Metabolic diseases	Observational studies	J Diabetes Sci Technol. 2020 Jan14(1):105-111	JOURNAL OF DIABETES SCIENCE AND TECHNOLOGY	NA	NA	18	Follow up	10.1177/1932296819861991
Personal Experiences With COVID-19 and Diabetes Technology: All for Technology Yet Not Technology for All	Monaghan M, Marks B.	United States of America	North America	Comment/editorial/letter	2020	Diabetes mellitus type I	Metabolic diseases	Review/meta-analysis	J Diabetes Sci Technol. 2020 Jul14(4):762-763	JOURNAL OF DIABETES SCIENCE AND TECHNOLOGY	NA	NA	2	Follow up	10.1177/1932296820930005
Benefits of Technology in the Age of COVID-19 and Diabetes. .Mobile Phones From a Rwanda Perspective	Krisiunas E, Sibomana L.	United States of America	North America	Article	2020	Diabetes mellitus type I	Metabolic diseases	Case report/case series	J Diabetes Sci Technol. 2020 Jul14(4):748-749	JOURNAL OF DIABETES SCIENCE AND TECHNOLOGY	NA	NA	4	Diagnosis & Treatment/Follow-up	10.1177/1932296820930032
WEARCON: wearable home monitoring in children with asthma reveals a strong association with hospital	van der Kamp MR, Klaver EC, Thio BJ, Driessen JMM, de Jongh FHC, Tabak M, van der Palen J, Hermens HJ.	United Kingdom	Europe	Article	2020	Asthma	Asthma	Interventional studies	BMC Med Inform Decis Mak. 2020 Aug 1420(1):192	BMC MEDICAL INFORMATICS AND DECISION MAKING	NA	NA	5	Follow up	10.1186/s12911-020-01210-1

based assessment of asthma control															
Remote monitoring and treatment of children and adolescents with type 1 diabetes	Laptev D.N., Emelyanov A.O., Samoilova Y.G., Khramova E.B., Petriaikina E.E., Rybkina I.G., Filimonova A.Y., Peterkova V.A.	Russia	Europe	Article	2020	Diabetes mellitus type I	Metabolic diseases	Interventional studies	Problemy endokrinologii (2020) 66:4 (50-60)	PROBLEMY ENDOKRINOL OGII	NA	NA	0	Follow up	10.14341/pro bl12201
Model for Taking Care of Patients with Early Childhood Caries during the SARS-Cov-2 Pandemic	Cianetti S, Pagano S, Nardone M, Lombardo G.	Italy	Europe	Article	2020	Caries of deciduous teeth	Dental caries	Review/meta-analysis	Int J Environ Res Public Health. 2020 May 2617(11):3751	ENVIRONME NTAL RESEARCH A ND PUBLIC HEALTH	NA	NA	21	Follow up	10.3390/ijerp h17113751
Feasibility and effectiveness of teleconsultation in children with epilepsy amidst the ongoing COVID-19 pandemic in a resource-limited country	Panda P.K., Dawman L., Panda P., Sharawat I.K.	India	Asia	Article	2020	Epilepsy	Brain disorders	Observational studies	Seizure (2020) 81 (29-35). Date of Publication: 1 Oct 2020	SEIZURE	0,798	NEUROSCIENC ES	30	Diagnosis & Treatment/F ollow-up	10.1016/j.seiz ure.2020.07.0 13
Tablet-based electroencephalography diagnostics for patients with epilepsy in the West African Republic of Guinea	Sokolov, E; Bachir, DHA; Sakadi, F; Williams, J; Vogel, AC; Schaekermann, M; Tassiou, N; Bah, AK; Khatri, V; Hotan, GC; Ayub, N; Leung, E; Fantaneanu, TA; Patel, A; Vyas, M; Milligan, T; Villamar, MF; Hoch, D; Purves, S; Esmaeili, B; Stanley, M; Lehn-Schioler, T; Tellez-Zenteno, J; Gonzalez-Giraldo, E; Tolokh, I; Heidarian, L; Worden, L; Jadeja, N; Fridinger, S; Lee, L; Law, E; Abass, CF; Mateen, FJ	United States of America	North America	Article	2020	Epilepsy	Brain disorders	Interventional studies	European journal of neurology vol. 27,8 (2020): 1570-1577.	EUROPEAN JOURNAL OF NEUROLOGY	6,577	NEUROSCIENC ES	3	Diagnosis & Treatment/F ollow-up	10.1111/ene.1 4291
Tele-assisted behavioral intervention for families with children with autism spectrum disorders: A randomized control trial	Marino F., Chilà P., Failla C., Crimi I., Minutoli R., Puglisi A., Arnao A.A., Tartarisco G., Ruta L., Vagni D., Pioggia G.	Italy	Europe	Article	2020	Autism spectrum disorders	Neurodev elopmenta l diseases or disorders	Interventional studies	Brain Sciences (2020) 10:9 (1-12)	BRAIN SCIENCES	3,394	NEUROSCIENC ES	13	Treament	10.3390/brain sci10090649
Using Hybrid Telepractice for Supporting Parents of Children with ASD during the COVID-19 Lockdown: A Feasibility Study in Iran	Samadi, SA; Bakhshalizadeh-Moradi, S; Khandani, F; Foladgar, M; Poursaid-Mohammad, M; McConkey, R	Iran	Asia	Article	2020	Autism spectrum disorders	Neurodev elopmenta l diseases or disorders	Observational studies	Brain Sciences 2020 1 0:11 (1-14)	BRAIN SCIENCES	3,394	NEUROSCIENC ES	8	Treament	10.3390/brain sci10110892

Pediatric diabetic retinopathy telescreening	Strul S, Zheng Y, Gangaputra S, Datye K, Chen Q, Maynard L, Pittel E, Russell W, Donahue S.	United States of America	North America	Article	2020	Diabetes mellitus type I	Metabolic diseases	Observational studies	J AAPOS. 2020 Feb24(1):10.e1-10.e5	JOURNAL OF AAPOS	1,22	PEDIATRICS	3	Diagnosis	10.1016/j.jaapos.2019.10.010
A Multimodal Telehealth Strategy to Improve Pediatric Epilepsy Care	Niemann, MH; Alvarado, MC; Camayd-Munoz, C; Jonas, RR; Wenger, JK; Douglass, LM	United States of America	North America	Article	2020	Epilepsy	Brain disorders	Review/meta-analysis	Pediatr Clin North Am. 2020 Aug;67(4):629-634	PEDIATRIC CLINICS OF N ORTH AMERICA	3,278	PEDIATRICS	2	Follow up	10.1016/j.pcl.2020.04.004
Implementing Telehealth in Pediatric Type 1 Diabetes Mellitus	Fogel JL, Raymond JK.	United States of America	North America	Article	2020	Diabetes mellitus type I	Metabolic diseases	Review/meta-analysis	Pediatr Clin North Am. 2020 Aug67(4):661-664	PEDIATRIC CLINICS OF N ORTH AMERICA	3,278	PEDIATRICS	8	Trearment	10.1016/j.pcl.2020.04.009
Pediatric Asthma Management During the COVID-19 Pandemic: Results of a National Survey	Sanchez-Garcia, S; Ruiz-Hornillos, J; Escudero, C	Spain	Europe	Article	2020	Asthma	Asthma	Observational studies	(2020) Pediatric, Allergy, Immunology, and Pulmonology, 33 (4) , pp. 199-203.	PEDIATRIC, ALLERGY, IMMUNOLOG Y, AND PULMONOLO GY	1,349	PEDIATRICS	2	Follow up	10.1089/ped.2020.1250
Management of Infantile Spasms During the COVID-19 Pandemic	Grinspan, ZM; Mytinger, JR; Baumer, FM; Ciliberto, MA; Cohen, BH; Dlugos, DJ; Harini, C; Hussain, SA; Joshi, SM; Keator, CG; Knupp, KG; McGoldrick, PE; Nickels, KC; Park, JT; Pasupuleti, A; Patel, AD; Shahid, AM; Shellhaas, RA; Shrey, DW; Singh, RK; Wolf, SM; Yozawitz, EG; Yuskaitis, CJ; Waugh, JL; Pearl, PL	United States of America	North America	Article	2020	Epilepsy	Brain disorders	Review/meta-analysis	J Child Neurol. 2020 Oct;35(12):828-834	JOURNAL OF CHILD NEUROLOGY	1,987	PEDIATRICS	18	Diagnosis & Treatment/F ollow-up	10.1177/088307382093373 9
Care Delivery for Children With Epilepsy During the COVID-19 Pandemic: An International Survey of Clinicians	Wirrell, EC; Grinspan, ZM; Knupp, KG; Jiang, YW; Hammeed, B; Mytinger, JR; Patel, AD; Nabbout, R; Specchio, N; Cross, JH; Shellhaas, RA	United States of America	North America	Article	2020	Epilepsy	Brain disorders	Observational studies	J Child Neurol. 2020 Nov;35(13):924-933	JOURNAL OF CHILD NEUROLOGY	1,987	PEDIATRICS	24	Follow up	10.1177/088307382094018 9
Telehealth delivery of adherence and medication management system improves outcomes in inner-city children with asthma	Lin NY, Ramsey RR, Miller JL, McDowell KM, Zhang N, Hommel K, Guilbert TW.	United States of America	North America	Article	2020	Asthma	Asthma	Observational studies	Pediatr Pulmonol. 2020 Apr55(4):858-865	PEDIATRIC DIABETES	3,039	PEDIATRICS	16	Trearment	10.1002/ppul.24623

Introducing telehealth and adherence monitoring to school-centered asthma management	Ross KR, Szefler SJ.	United States of America	North America	Comment/editorial/letter	2020	Asthma	Asthma	Review/meta-analysis	Pediatr Pulmonol. 2020 Mar55(3):565-567	PEDIATRIC DIABETES	3,039	PEDIATRICS	0	Follow up	10.1002/ppul.24663
School-Based Telemedicine Interventions for Asthma: A Systematic Review	Kim CH, Lieng MK, Rylee TL, Gee KA, Marcin JP, Melnikow JA.	United States of America	North America	Article	2020	Asthma	Asthma	Review/meta-analysis	Acad Pediatr. 2020 Sep-Oct20(7):893-901	ACADEMIC PEDIATRICS	3,107	PEDIATRICS	10	Trearment	10.1016/j.acap.2020.05.008
Implementing Telehealth in Pediatric Asthma	Perry TT, Margiotta CA.	United States of America	North America	Article	2020	Asthma	Asthma	Review/meta-analysis	Pediatr Clin North Am. 2020 Aug67(4):623-627	PEDIATRIC CLINICS OF N ORTH AMERICA	3,278	PEDIATRICS	3	Prevention	10.1016/j.pcl.2020.04.003
Economic evaluation of interventions for the treatment of asthma in children: A systematic review	Halmai LA, Neilson AR, Kilonzo M.	multicente r	More than one continent	Article	2020	Asthma	Asthma	Review/meta-analysis	Pediatr Allergy Immunol. 2020 Feb31(2):150-157	PEDIATRIC ALLERGY AN D IMMUNOLO GY	6,377	PEDIATRICS	1	Diagnosis & Treatment/F ollow-up	10.1111/pai.13129
Managing childhood allergies and immunodeficiencies during respiratory virus epidemics – The 2020 COVID-19 pandemic: A statement from the EAACI-section on pediatrics	Brough H.A., Kalayci O., Sediva A., Untersmayr E., Munblit D., Rodriguez del Rio P., Vazquez-Ortiz M., Arasi S., Alvaro-Lozano M., Tsabouri S., Galli E., Beken B., Eigenmann P.A.	multicente r	More than one continent	Article	2020	Asthma	Asthma	Review/meta-analysis	Pediatric Allergy and Immunology (2020) 31:5 (442-448)	PEDIATRIC ALLERGY AND IMMUNOLOG Y	6,377	PEDIATRICS	68	Follow up	10.1111/pai.13262
Intervention to reduce hypoglycemia fear in parents of young kids using video-based telehealth (REDCHiP)	Patton SR, Clements MA, Marker AM, Nelson EL.	United States of America	North America	Article	2020	Diabetes mellitus type I	Metabolic diseases	Interventional studies	Pediatr Diabetes. 2020 Feb21(1):112-119	PEDIATRIC DIABETES	4,866	PEDIATRICS	13	Trearment	10.1111/pedi.12934
Outcomes of monthly video consultations as an add-on to regular care for children with type 1 diabetes: A 6-month quasi-randomized clinical trial followed by an extension phase	von Sengbusch S, Eisemann N, Mueller-Godeffroy E, Lange K, Doerdelmann J, Erdem A, Menrath I, Bokelmann J, Krasmann M, Kaczmarczyk P, Bertram B, Hiort O, Katalinic A, Frielitz FS.	Germany	Europe	Article	2020	Diabetes mellitus type I	Metabolic diseases	Interventional studies	Pediatr Diabetes. 2020 Dec21(8):1502-1515	PEDIATRIC DIABETES	4,866	PEDIATRICS	13	Follow up	10.1111/pedi.13133
Telehealth remote patient monitoring: An effective tool for improving outcomes in children with poorly controlled diabetes	Armstrong M.K., Sukumaran A., Pasha S., Clemente E., Luckey K., Dixit N.	United States of America	North America	Conference abstract	2020	Diabetes mellitus type I	Metabolic diseases	Interventional studies	Hormone Research in Paediatrics (2017) 88 Supplement 1 (604-605). Date of Publication: 2017	HORMONE RESEARCH IN PAEDIATRICS	2,852	PEDIATRICS	0	Follow up	10.1159/000481424

COVID-19: Technology-Supported Remote Assessment of Pediatric Asthma at Home	van der Kamp, MR; Tabak, M; de Rooij, SEJA; van Lierop, PPE; Thio, BJ	Netherland	Europe	Article	2020	Asthma	Asthma	Review/meta-analysis	Front Pediatr. 2020 Sep 8;8:529	FRONTIERS IN PEDIATRICS	3,418	PEDIATRICS	0	Follow up	10.3389/fped.2020.00529
Transition to Adult Care in Youth with Epilepsy: One Center's Experience with a Transition Program and Its Integration within Telemedicine	Jones G.L., Hickam T., Wellman C., Modrcin A., Abdelmoity A., Le Pichon J.-B.	United States of America	North America	Article	2020	Epilepsy	Brain disorders	Case report/ case series	Journal of Pediatric Epilepsy (2020) 9:4 (119-124)	JOURNAL OF PEDIATRIC EPILEPSY	0,06	PEDIATRICS	0	Follow up	I https://doi.org/ 10.1055/s-0040-1716826
Electronic Mental Health as an Option for Egyptian Psychiatry: Cross-Sectional Study	Kamel, MM; Westenberg, JN; Choi, F; Tabi, K; Badawy, A; Ramy, H; Elsawi, H; Krausz, M	Canada	North America	Article	2020	Major depressive disorder	Mental disorders	Observational studies	JMIR Ment Health. 2020 Aug 13;7(8):e19591.	JMIR MENTAL HEALTH	4,388	PSYCHIATRY	5	Follow up	10.2196/19591
Future of psychiatry	Shejekar S., Nemlekar S., Srivastava A.	India	Asia	Conference abstract	2020	Autism spectrum disorders	Neurodevelopmental diseases or disorders	Review/meta-analysis	Indian Journal of Psychiatry (2020) 62:7 Supplement 1 (S121)	INDIAN JOURNAL OF PSYCHIATRY	1,759	PSYCHIATRY	0	Diagnosis & Treatment/Follow-up	NA
NOVEL EARLY INTERVENTION IN AUTISM SPECTRUM DISORDER: BASIC PRINCIPLES AND NEW RESEARCH	Hardan A., Gengoux G.W., Veenstra-VanderWeele J.	United States of America	North America	Conference abstract	2020	Autism spectrum disorders	Neurodevelopmental diseases or disorders	Case report/ case series	Journal of the American Academy of Child and Adolescent Psychiatry (2020) 59:10 Supplement (S271)	JOURNAL OF THE AMERICAN ACADEMY OF CHILD AND ADOLESCENT PSYCHIATRY	8,829	PSYCHIATRY	0	Trearment	10.1016/j.jaac.2020.07.565
From Digital Mental Health Interventions to Digital Addiction: Where the Two Fields Converge	Elias Aboujaoude , Lina Gega	United States of America	North America	Article	2020	Major depressive disorder	Mental disorders	Review/meta-analysis	Front Psychiatry. 2020 Jan 21;10:1017	FRONTIERS IN PSYCHIATRY	4,157	PSYCHIATRY	4	Diagnosis & Treatment/Follow-up	10.3389/fpsyt.2019.01017
Using a Smartphone App and Clinician Portal to Enhance Brief Cognitive Behavioral Therapy for Childhood Anxiety Disorders	Silk JS, Pramana G, Sequeira SL, Lindhiem O, Kendall PC, Rosen D, Parmanto B.	United States of America	North America	Article	2020	Anxiety disorders	Mental disorders	Interventional studies	Behav Ther. 2020 Jan51(1):69-84	BEHAVIOR THERAPY	4,183	PSYCHIATRY	16	Trearment	10.1016/j.beth.2019.05.002
Interpretation Bias Modification Versus Progressive Muscle Relaxation for Social Anxiety Disorder: A Web-Based Controlled Trial	Cougle JR, Wilver NL, Day TN, Summers BJ, Okey SA, Carlton CN.	United States of America	North America	Article	2020	Anxiety disorders	Mental disorders	Interventional studies	Behav Ther. 2020 Jan51(1):99-112	BEHAVIOR THERAPY	4,183	PSYCHIATRY	10	Trearment	10.1016/j.beth.2019.05.009
4.1 PIVOTAL RESPONSE TREATMENT: APPLICATION TO NEW POPULATIONS AND SERVICE-DELIVERY MODELS	Gengoux G.W., Shahabuddin A., Schwartzman J., Schuck R., Strong K., Ardel C., Hardan A.	United States of America	North America	Conference abstract	2020	Autism spectrum disorders	Neurodevelopmental diseases or disorders	Review/meta-analysis	Journal of the American Academy of Child and Adolescent Psychiatry	JOURNAL OF THE AMERICAN ACADEMY OF CHILD AND	8,829	PSYCHIATRY	0	Diagnosis & Treatment/Follow-up	10.1016/j.jaac.2020.07.566

									(2020) 59:10 Supplement (S271-S272). Date of Publication: 1 Oct 2020	ADOLESCENT PSYCHIATRY					
32.3 ONLINE HELP-SEEKING PRIOR TO DIAGNOSIS: CAN WEB-BASED RESOURCES HELP REDUCE THE BURDEN OF MOOD DISORDERS IN YOUNG PEOPLE?	Van Meter A., Birnbaum M., Ali A., Kane J.	United States of America	North America	Conference abstract	2020	Anxiety disorders	Mental disorders	Observational studies	Journal of the American Academy of Child and Adolescent Psychiatry (2020) 59:10 Supplement (S315-S316)	JOURNAL OF THE AMERICAN ACADEMY OF CHILD AND ADOLESCENT PSYCHIATRY	8,829	PSYCHIATRY	4	Diagnosis & Treatment/Follow-up	10.1016/j.jaac.2020.07.739
51.2 CHALLENGES WITH MANAGING CHILDREN AND ADOLESCENTS WITH ADHD DURING THE COVID-19 PANDEMIC: A REVIEW OF THE LITERATURE	McGowan G., Conrad R., Potts H.	United States of America	North America	Conference abstract	2020	Attention-deficit/hyperactivity disorder	Neurodevelopmental diseases or disorders	Review/meta-analysis	Journal of the American Academy of Child and Adolescent Psychiatry (2020) 59:10 Supplement (S251)	JOURNAL OF THE AMERICAN ACADEMY OF CHILD AND ADOLESCENT PSYCHIATRY	8,829	PSYCHIATRY	4	Diagnosis & Treatment/Follow-up	10.1016/j.jaac.2020.08.412
Telemedicine in the Management of ADHD: Literature Review of Telemedicine in ADHD	Spencer, T; Noyes, E; Biederman, J	United States of America	North America	Article	2020	Attention-deficit/hyperactivity disorder	Neurodevelopmental diseases or disorders	Review/meta-analysis	Journal of Attention Disorders. 2020;24(1):3-9	JOURNAL OF ATTENTION DISORDERS	3,256	PSYCHIATRY	17	Trearment	10.1177/1087054719859081
Functional assessment and function-based treatment delivered via telehealth: A brief summary	Schieltz, KM; Wacker, DP	United States of America	North America	Article	2020	Conduct disorder	Neurodevelopmental diseases or disorders	Review/meta-analysis	J Appl Behav Anal. 2020 Jul;53(3):1242-1258	JOURNAL OF APPLIED BEHAVIOR ANALYSIS	3,695	PSYCHOLOGY, CLINICAL	19	Trearment	10.1002/jaba.742
Evaluation of a telehealth parent training program in teaching self-care skills to children with autism	Boutain AR, Sheldon JB, Sherman JA.	United States of America	North America	Article	2020	Autism spectrum disorders	Neurodevelopmental diseases or disorders	Case report/ case series	J Appl Behav Anal. 2020 Jul53(3):1259-1275	JOURNAL OF APPLIED BEHAVIOR ANALYSIS	3,695	PSYCHOLOGY, CLINICAL	11	Follow up	10.1002/jaba.743
A randomized clinical trial of a virtual-training program for teaching applied-behavior-analysis skills to parents of children with autism spectrum disorder	Fisher WW, Luczynski KC, Blowers AP, Vosters ME, Pisman MD, Craig AR, Hood SA, Machado MA, Lesser AD, Piazza CC.	United States of America	North America	Article	2020	Autism spectrum disorders	Neurodevelopmental diseases or disorders	Interventional studies	J Appl Behav Anal. 2020 Sep53(4):1856-1875	JOURNAL OF APPLIED BEHAVIOR ANALYSIS	3,695	PSYCHOLOGY, CLINICAL	4	Trearment	10.1002/jaba.778
A Randomized Controlled Trial of Functional Communication Training via Telehealth for Young Children with Autism Spectrum Disorder	Lindgren S, Wacker D, Schieltz K, Suess A, Pelzel K, Kopelman T, Lee J, Romani P, O'Brien M.	United States of America	North America	Article	2020	Autism spectrum disorders	Neurodevelopmental diseases or disorders	Interventional studies	J Autism Dev Disord. 2020 Dec50(12):4449-4462	JOURNAL OF AUTISM AND DEVELOPMENTAL DISORDERS	4,291	PSYCHOLOGY, DEVELOPMENTAL	23	Trearment	10.1007/s10803-020-04451-1

140. Early Lessons Learned from Implementing Telehealth in Rural School Based Health Centers	Vallabhan M.K., Jimenez E.Y., McCauley G., Kong A.S.	Mexico	South America	Conference abstract	2020	Asthma	Asthma	Case report/ case series	Journal of Adolescent Health (2020) 66:2 Supplement (S71-S72)	JOURNAL OF ADOLESCENT HEALTH	4,828	PSYCHOLOGY, DEVELOPMENTAL	0	Diagnosis	10.1016/j.jadohealth.2019.11.143
Brief Report: A Pilot Online Pivotal Response Treatment Training Program for Parents of Toddlers with Autism Spectrum Disorder	McGarry E, Vernon T, Baktha A.	United States of America	North America	Article	2020	Autism spectrum disorders	Neurodevelopmental diseases or disorders	Interventional studies	J Autism Dev Disord. 2020 Sep50(9):3424-3431	JOURNAL OF AUTISM AND DEVELOPMENTAL DISORDERS	4,291	PSYCHOLOGY, DEVELOPMENTAL	8	Trearment	10.1007/s10803-019-04100-2
Brief Report: Preliminary Feasibility of the TEDI: A Novel Parent-Administered Telehealth Assessment for Autism Spectrum Disorder Symptoms in the First Year of Life	Talbott MR, Dufek S, Zwaigenbaum L, Bryson S, Brian J, Smith IM, Rogers SJ.	United States of America	North America	Article	2020	Autism spectrum disorders	Neurodevelopmental diseases or disorders	Interventional studies	J Autism Dev Disord. 2020 Sep50(9):3432-3439	JOURNAL OF AUTISM AND DEVELOPMENTAL DISORDERS	4,291	PSYCHOLOGY, DEVELOPMENTAL	16	Diagnosis	10.1007/s10803-019-04314-4
Bridging the Needs of Adolescent Diabetes Care During COVID-19: A Nurse-Led Telehealth Initiative	Lim ST, Yap F, Chin X.	Singapore	Asia	Article	2020	Diabetes mellitus type I	Metabolic diseases	Interventional studies	J Adolesc Health. 2020 Oct67(4):615-617	JOURNAL OF ADOLESCENT HEALTH	4,828	PSYCHOLOGY, DEVELOPMENTAL	7	Trearment	10.1016/j.jadohealth.2020.07.012
An eHealth Program for Parents of Adolescents With T1DM Improves Parenting Stress: A Randomized Control Trial	Whittemore R, Coleman J, Delvy R, Zincavage R, Ambrosoli JA, Shi L, Kato B, Marceau L.	United States of America	North America	Article	2020	Diabetes mellitus type I	Metabolic diseases	Interventional studies	Diabetes Educ. 2020 Feb46(1):62-72	DIABETES EDUCATOR	2,14	PUBLIC, ENVIRONMENTAL & OCCUPATIONAL HEALTH	1	Trearment	10.1177/0145721719890372
Working Toward an mHealth Platform for Adolescents with Type 1 Diabetes: Focus Groups With Teens, Parents, and Providers	Sinisterra M, Kelly KP, Shneider C, El-Zein A, Swartwout E, Deyo P, Streisand R.	United States of America	North America	Article	2020	Diabetes mellitus type I	Metabolic diseases	Interventional studies	Diabetes Educ. 2020 Oct46(5):444-454	DIABETES EDUCATOR	2,14	PUBLIC, ENVIRONMENTAL & OCCUPATIONAL HEALTH	0	Trearment	10.1177/0145721720943123
Reducing Emotional Distress for Childhood Hypoglycemia in Parents (REDCHiP): Protocol for a Randomized Clinical Trial to Test a Video-Based Telehealth Intervention	Patton, SR; McConville, A; Marker, AM; Monzon, AD; Driscoll, KA; Clements, MA	United States of America	North America	Article	2020	Diabetes mellitus type I	Metabolic diseases	Study protocol/Pilot study	JMIR Res Protoc. 2020 Aug 18;9(8):e17877	JMIR RESEARCH PROTOCOLS	0,67	PUBLIC, ENVIRONMENTAL & OCCUPATIONAL HEALTH	0	Trearment	10.2196/17877
Managing Asthma and Obesity Related Symptoms (MATADORS): An mHealth Intervention to Facilitate Symptom Self-Management among Youth	Nichols M, Teufel R, Miller S, Madisetti M, Giovanni CS, Chike-Harris K, Jones L, Prentice M, Ruggiero K, Kelechi T.	United States of America	North America	Article	2020	Asthma	Asthma	Interventional studies	Int J Environ Res Public Health. 2020 Oct 2317(21):7750	ENVIRONMENTAL RESEARCH AND PUBLIC HEALTH	3,39	PUBLIC, ENVIRONMENTAL & OCCUPATIONAL HEALTH	2	Follow up	10.3390/ijerph17217750

Detection of Asthma Exacerbation in Adolescent and Adult Subjects with Chronic Asthma Using A Cough-Centred, Smartphone-Based Algorithm	Claxton S., Porter P., Brisbane J., Bear N., Peltonin V., Woods J., Smith C., Purdie F., Abeyratne U.	Australia	Oceania	Conference abstract	2020	Asthma	Asthma	Observational studies	Respirology (2020) 25 (115)	RESPIROLOGY	6,424	RESPIRATORY SYSTEM	0	Diagnosis	10.1111/resp.13778
Pilot study of an emr-integrated smartphone-telemedicine program as a virtual primary care extension for underserved younger adults with asthma (Teams-Technology Enabled Asthma Management System)	Mammen J.R., Halterman J., Berliant M.N., Turgeon K., Philibert A., Java J., Reznik M., Feldman J.M., Fortuna R., Schoonmaker J.D., Crowley A., Frey S.M., Arcoleo K.J.	United States of America	North America	Conference abstract	2020	Asthma	Asthma	Interventional studies	American Journal of Respiratory and Critical Care Medicine (2020) 201:1	AMERICAN JOURNAL OF RESPIRATORY AND CRITICAL CARE MEDICINE	21,405	RESPIRATORY SYSTEM	0	Trearment	https://doi.org/10.1164/ajrccm-conference.2020.201.1_MeetingAbstracts.A7756
Are Caregivers Of Pediatric Patients With Asthma Willing To Use Telemedicine To Prevent Emergency Room Visits?	Padam J.	United States of America	North America	Conference abstract	2021	Asthma	Asthma	Observational studies	Journal of Allergy and Clinical Immunology (2021) 147:2 Supplement (AB51)	JOURNAL OF ALLERGY AND CLINICAL IMMUNOLOGY	10,793	ALLERGY	0	Diagnosis	10.1016/j.jaci.2020.12.212
An Institutional Survey of Patient Satisfaction with Telemedicine Services in Pediatric Allergy During the COVID-19 Pandemic	Lanier K., Kuruvilla M., Shih J.	United States of America	North America	Conference abstract	2021	Asthma	Asthma	Observational studies	Journal of Allergy and Clinical Immunology (2021) 147:2 Supplement (AB169)	JOURNAL OF ALLERGY AND CLINICAL IMMUNOLOGY	10,793	ALLERGY	0	Diagnosis	10.1016/j.jaci.2020.12.600
The rise of telemedicine in a tertiary paediatric allergy service-how often and for which patients?	Collis H., Sonmez-Ajtai S., Hayes E.	United Kingdom	Europe	Conference abstract	2021	Asthma	Asthma	Observational studies	Clinical and Experimental Allergy (2021) 51:1 (164)	CLINICAL AND EXPERIMENTAL ALLERGY	5,018	ALLERGY	0	Follow up	10.1111/cea.13812 Entry date 2021-02-25 (Full record)
Ten Rules for Implementation of a Telemedicine Program to Care for Patients with Asthma	Persaud, YK; Portnoy, JM	United States of America	North America	Article	2021	Asthma	Asthma	Review/meta-analysis	J Allergy Clin Immunol Pract. 2021 Jan;9(1):13-21	JOURNAL OF ALLERGY AND CLINICAL IMMUNOLOGY: IN PRACTICE	8,861	ALLERGY	11	Follow up	10.1016/j.jaip.2020.10.005
Clinical and economic impact of telemedicine in the management of pediatric asthma in Jordan: a pharmacist-led intervention	Shdaifat M.B.M., Khasawneh R.A., Alefan Q.	Jordan	Asia	Article	2021	Asthma	Asthma	Interventional studies	J Asthma. 2021 May 8:1-11	JOURNAL OF ASTHMA	2,515	ALLERGY	0	Trearment	10.1080/02770903.2021.1924774
Digital health interventions in children with asthma	Ferrante G, Licari A, Marseglia GL, La Grutta S.	Italy	Europe	Article	2021	Asthma	Asthma	Review/meta-analysis	Clin Exp Allergy. 2021 Feb51(2):212-220	CLINICAL AND EXPERIMENTAL ALLERGY	5,018	ALLERGY	10	Diagnosis & Treatment/Follow-up	10.1111/cea.13793

Paediatric and Adolescent Asthma: A Narrative Review of Telemedicine and Emerging Technologies for the Post COVID-19 Era	Davies B., Kenia P., Nagakumar P., Gupta A.	United Kingdom	Europe	Article	2021	Asthma	Asthma	Review/meta-analysis	Clinical and experimental allergy : journal of the British Society for Allergy and Clinical Immunology (2021)	CLINICAL AND EXPERIMENTAL ALLERGY	5,018	ALLERGY	10	Treatment	10.1111/cea.13836
Paediatric and adolescent asthma: A narrative review of telemedicine and emerging technologies for the post-COVID-19 era	Davies B, Kenia P, Nagakumar P, Gupta A.	United Kingdom	Europe	Article	2021	Asthma	Asthma	Review/meta-analysis	Clin Exp Allergy. 2021 Mar51(3):393-401	CLINICAL AND EXPERIMENTAL ALLERGY	5,018	ALLERGY	11	Treatment	10.1111/cea.13836
Diagnosing and managing childhood absence epilepsy by telemedicine	Stafstrom C.E., Sun L.R., Kossoff E.H., Dabrowski A.K., Singhi S., Kelley S.A.	United States of America	North America	Article	2021	Epilepsy	Brain disorders	Case report/ case series	Epilepsy Behav. 2021 Feb;115:107404	EPILEPSY AND BEHAVIOR	2,937	BEHAVIORAL SCIENCES	3	Diagnosis & Treatment/Follow-up	10.1016/j.yebeh.2020.107404
Impact of COVID-19 pandemic in pediatric patients with epilepsy with neuropsychiatric comorbidities: A telemedicine evaluation	Pasca L., Zanaboni M.P., Grumi S., Totaro M., Ballante E., Varesio C., De Giorgis V.	Italy	Europe	Article	2021	Epilepsy	Brain disorders	Observational studies	Epilepsy and Behavior (2021) 115	EPILEPSY AND BEHAVIOR	2,937	BEHAVIORAL SCIENCES	8	Follow up	10.1016/j.yebeh.2020.107519
Association between telemedicine and incidence of status epilepticus during the COVID-19 pandemic	Kubota T., Kuroda N.	multicenter	More than one continent	Article	2021	Epilepsy	Brain disorders	Observational studies	Epilepsy and Behavior (2021) 124 Article Number: 108303	EPILEPSY AND BEHAVIOR	2,937	BEHAVIORAL SCIENCES	5	Follow up	10.1016/j.yebeh.2021.108303
Feasibility of using "SMARTER" methodology for monitoring precipitating conditions of pediatric migraine episodes	Connelly MA, Boorigie ME.	United States of America	North America	Article	2021	Migraine	Brain disorders	Interventional studies	Headache. 2021 Mar61(3):500-510	HEADACHE	5,887	CLINICAL NEUROLOGY	1	Follow up	10.1111/head.14028
Early impact of the COVID-19 pandemic on outpatient migraine care in Hawaii: Results of a quality improvement survey	Smith M, Nakamoto M, Crocker J, Tiffany Morden F, Liu K, Ma E, Chong A, Van N, Vajjala V, Carrazana E, Viereck J, Liow K.	United States of America	North America	Article	2021	Migraine	Brain disorders	Observational studies	Headache. 2021 Jan61(1):149-156	HEADACHE	5,887	CLINICAL NEUROLOGY	8	Follow up	10.1111/head.14030
Remote electrical neuromodulation for acute treatment of migraine in adolescents	Hershey AD, Lin T, Gruper Y, Harris D, Ironi A, Berk T, Szperka CL, Berenson F.	United States of America	North America	Article	2021	Migraine	Brain disorders	Interventional studies	Headache. 2021 Feb61(2):310-317	HEADACHE	5,887	CLINICAL NEUROLOGY	6	Treatment	10.1111/head.14042
Factors Associated With, and Mitigation Strategies for, Health Care Disparities Faced by Patients With Headache Disorders	Kiarashi J. (jkiarashi@gmail.com) ,VanderPluym J.,Szperka C.L.,Turner S.,Minen M.T.,Broner S.,Ross A.C.,Wagstaff	United States of America	North America	Article	2021	Migraine	Brain disorders	Review/meta-analysis	Neurology. 2021 Aug 10;97(6):280-289	NEUROLOGY	9,91	CLINICAL NEUROLOGY	2	Diagnosis & Treatment/Follow-up	10.1212/WNL.0000000000012261

	A.E.,Anto M.,Marzouk M.,Monteith T.S.,Rosen N.,Manrriquez S.L.,Seng E.,Finkel A.,Charleston L.														
"How to" operate a pediatric neuropsychology practice during the COVID-19 pandemic: Real tips from one practice's experience	Loman M., Vogt E., Miller L., Landsman R., Duong P., Kasten J., DeFrancisco D., Koop J., Heffelfinger A.	United States of America	North America	Article	2021	Autism spectrum disorders	Neurodevelopmental diseases or disorders	Guidelines/consensus paper	Child Neuropsychology (2021) 27:2 (251-279)	CHILD NEUROPSYCHOLOGY	2,3	CLINICAL NEUROLOGY	10	Prevention	10.1080/09297049.2020.1830962
Telemedicine Applications for the Evaluation of Patients with Non-Acute Headache: A Narrative Review	Noutsios, CD; Boisvert-Plante, V; Perez, J; Hudon, J; Ingelmo, P	Canada	North America	Article	2021	Tension-type headache	Brain disorders	Review/meta-analysis	Journal of pain research vol. 14 1533-1542. 1 Jun. 2021	JOURNAL OF PAIN RESEARCH	3,133	CLINICAL NEUROLOGY	4	Trearment	10.2147/JPR.S309542
Telemedicine in epilepsy care: doc-to-doc applications Part I: State of the art, challenges, and perspectives	Mues, S; Hamer, HM; von Podewils, F; Sotoodeh, A; Rosenow, F; Wellmer, J; Zoellner, JP	Germany	Europe	Article	2021	Epilepsy	Brain disorders	Review/meta-analysis	Z. Epileptol. 34, 294–298 (2021)	ZEITSCHRIFT FUR EPILEPTOLOGIE	0,13	CLINICAL NEUROLOGY	0	Follow up	10.1007/s10309-021-00424-1
Mobile photographic screening for dental caries in children: Diagnostic performance compared to unaided visual dental examination	Estai, M; Kanagasingam, Y; Mehdizadeh, M; Vignarajan, J; Norman, R; Huang, BY; Spallek, H; Irving, M; Arora, A; Kruger, E; Tennant, M	Australia	Oceania	Article	2021	Caries of deciduous teeth	Dental caries	Interventional studies	J Public Health Dent. 2021 Jan 26	JOURNAL OF PUBLIC HEALTH DENTISTRY	1,821	DENTISTRY, ORAL SURGERY & MEDICINE	0	Diagnosis	10.1111/jphd.12443
CariesCare International adapted for the pandemic in children: Caries OUT multicentre single-group interventional study protocol	Martignon S, Cortes A, Douglas GVA, Newton JT, Pitts NB, Avila V, Usuga-Vacca M, Gamboa LF, Deery C, Abreu-Placeres N, Bonifacio C, Braga MM, Carletto-Körber F, Castro P, P Cerezo M, Chavarría N, Cifuentes OL, Echeverri B, Jácome-Liévano S, Kuzmina I, Lara JS, Manton D, Martínez-Mier EA, Melo P, Muller-Bolla M, Ochoa E, Osorio JR, Ramos K, Sanabria AF, Sanjuán J, San-Martín M, Squassi A, Velasco AK, Villena R, Zandona AF, Beltrán EO.	multicenter	More than one continent	Article	2021	Caries of permanent teeth	Dental caries	Observational studies	BMC Oral Health. 2021 Jul 121(1):329	BMC ORAL HEALTH	2,757	DENTISTRY, ORAL SURGERY & MEDICINE	2	Diagnosis & Treatment/Follow-up	10.1186/s12903-021-01674-1

Teledentistry and its applications in paediatric dentistry: A literature review	Sharma, H; Suprabha, BS; Rao, A	India	Asia	Article	2021	Caries of deciduous teeth	Dental caries	Review/meta-analysis	Pediatric Dental Journal, Volume 31, Issue 3, 2021, Pages 203-215	PEDIATRIC DENTAL JOURNAL	6,089	DENTISTRY, ORAL SURGERY & MEDICINE	3	Treament	10.1016/j.pdj.2021.08.003
Mobile photographic screening for dental caries in children: Diagnostic performance compared to unaided visual dental examination	Estai, M; Kanagasingam, Y; Mehdizadeh, M; Vignarajan, J; Norman, R; Huang, BY; Spallek, H; Irving, M; Arora, A; Kruger, E; Tennant, M	Australia	Oceania	Article	2021	Caries of deciduous teeth	Dental caries	Interventional studies	Journal of public health dentistry. (2021): --. Web	JOURNAL OF PUBLIC HEALTH DENTISTRY	1,821	DENTISTRY, ORAL SURGERY & MEDICINE	0	Diagnosis	10.1111/jphd.12443
Development and evaluation of a gamified smart phone mobile health application for oral health promotion in early childhood: a randomized controlled trial	Zolfaghari M, Shirmohammadi M, Shahhosseini H, Mokhtaran M, Mohebbi SZ.	Iran	Asia	Article	2021	Caries of deciduous teeth	Dental caries	Interventional studies	BMC Oral Health. 2021 Jan 721(1):18	BMC ORAL HEALTH	2,757	DENTISTRY, ORAL SURGERY & MEDICINE	2	Prevention	10.1186/s12903-020-01374-2
Teledentistry Implementation in a Private Pediatric Dental Practice During the COVID-19 Pandemic	Brecher EA, Keels MA, Carrico CK, Hamilton DS.	United States of America	North America	Article	2021	Caries of deciduous teeth	Dental caries	Observational studies	Pediatr Dent. 2021 Nov 1543(6):463-467	PEDIATRIC DENTISTRY	1,874	DENTISTRY, ORAL SURGERY & MEDICINE	0	Treament	NA
Impact of the COVID-19 pandemic on children with psoriasis	Beytout Q, Pepiot J, Maruani A, Devulder D, Aubert R, Beylot-Barry M, Amici JM, Jullien D, Mahé E	France	Europe	Article	2021	Psoriasis	Skin diseases	Observational studies	Ann Dermatol Venereol. 2021 Jun;148(2):106-111	ANNALES DE DERMATOLOGIE ET DE VENEREOLOGIE	0,777	DERMATOLOGY	5	Follow up	10.1016/j.annder.2021.01.005
Asynchronous Teledermatology Assessment of Young Adult Acne Likely Concordant With In-Person Evaluation	Jacoby T, Woolard A, Chamoun S, Moy R.	Sweden	Europe	Article	2021	Acne vulgaris	Skin diseases	Interventional studies	J Drugs Dermatol. 2021 Apr 120(4):432-435	JOURNAL OF DRUGS IN DERMATOLOGY	2,114	DERMATOLOGY	1	Diagnosis	10.36849/JDD.2021.5688
Care for children with atopic dermatitis in the Netherlands during the COVID-19 pandemic: Lessons from the first wave and implications for the future	Ragamin A, de Wijs LEM, Hijnen DJ, Arends NJT, Schuttelaar MLA, Pasmans SGMA, Bronner MB.	Netherland	Europe	Article	2021	Atopic dermatitis	Skin diseases	Interventional studies	J Dermatol. 2021 Dec48(12):1863-1870	JOURNAL OF DERMATOLOGY	4,005	DERMATOLOGY	0	Follow up	10.1111/1346-8138.16130
Avatar-based Teledermatology to Address Access Barriers in Pediatric Atopic Dermatitis: Developing a Patient Guided Platform	Pollock S., Rice S.M., Thomas M., Hawryluk E.B., Kourosh A.S.	United States of America	North America	Conference abstract	2021	Atopic dermatitis	Skin diseases	Observational studies	Journal of Cutaneous Medicine and Surgery (2021) 25:1 SUPPL (58S-59S)	JOURNAL OF CUTANEOUS MEDICINE AND SURGERY	2,092	DERMATOLOGY	0	Follow up	10.1177/12034754211037815
A systematic review of technological approaches for autism spectrum disorder assessment in children: Implications for the COVID-19 pandemic	Dahiya AV, DeLucia E, McDonnell CG, Scarpa A.	United States of America	North America	Article	2021	Autism spectrum disorders	Neurodevelopmental diseases or disorders	Review/meta-analysis	Res Dev Disabil. 2021 Feb109:103852	RESEARCH IN DEVELOPMENTAL DISABILITIES	3,23	EDUCATION, SPECIAL	6	Diagnosis & Treatment/Follow-up	10.1016/j.ridd.2021.103852

Looking at Europe's recent behavioral telehealth practices for children and families impacted by neurodevelopmental disabilities	Kingsdorf, S; Pancocha, K	Czech Republic	Europe	Article	2021	Autism spectrum disorders	Neurodevelopmental diseases or disorders	Review/meta-analysis	International Journal of Developmental Disabilities (Article in press)	INTERNATIONAL JOURNAL OF DEVELOPMENTAL DISABILITIES	0,55	EDUCATION, SPECIAL	0	Trearment	10.1080/20473869.2021.1925403
It took a pandemic: Perspectives on impact, stress, and telehealth from caregivers of people with autism	White SW, Stoppelbein L, Scott H, Spain D.	United States of America	North America	Article	2021	Autism spectrum disorders	Neurodevelopmental diseases or disorders	Observational studies	Res Dev Disabil. 2021 Jun113:103938	RESEARCH IN DEVELOPMENTAL DISABILITIES	3,23	EDUCATION, SPECIAL	10	Trearment	10.1016/j.ridd.2021.103938
Internet-based versus face-to-face intervention training for parents of young children with excessive screen-time and autism spectrum disorder–like symptoms: a comparative study	Sadeghi S., Pouretemad H.R., Shalani B.	Iran	Asia	Article	2021	Autism spectrum disorders	Neurodevelopmental diseases or disorders	Interventional studies	International Journal of Developmental Disabilities (2021)	INTERNATIONAL JOURNAL OF DEVELOPMENTAL DISABILITIES	0,55	EDUCATION, SPECIAL	1	Follow up	10.1080/20473869.2021.1895699
A Technology-Enabled Adaptation of Face-to-Face Caregiver-Mediated JASPER Intervention: Preliminary Examination of Video Conferenced Caregiver Coaching	Shire SY, Worthman LB, Arbuckle S.	United States of America	North America	Article	2021	Autism spectrum disorders	Neurodevelopmental diseases or disorders	Interventional studies	Am J Intellect Dev Disabil. 2021 Sep 1126(5):421-434	AMERICAN JOURNAL ON INTELLECTUAL AND DEVELOPMENTAL DISABILITIES	2,5	EDUCATION, SPECIAL	1	Prevention	10.1352/1944-7558-126.5.421
Feasibility and Acceptability of a Synchronous Online Parent-Mediated Early Intervention for Children with Autism in a Low Resource Setting During COVID-19 Pandemic	Sengupta, K; Javeri, A; Mascarenhas, C; Khaparde, O; Mahadik, S	India	Asia	Article	2021	Autism spectrum disorders	Neurodevelopmental diseases or disorders	Observational studies	International Journal of Disability, Development and Education (2021)	INTERNATIONAL JOURNAL OF DISABILITY DEVELOPMENT AND EDUCATION	1,543	EDUCATION, SPECIAL	0	Follow up	https://doi.org/10.1080/1034912X.2021.1937957
Glycaemic control in the paediatric and young adult population with type 1 diabetes following a single telehealth visit - what have we learned from the COVID-19 lockdown?	Rachmiel M, Lebenthal Y, Mazor-Aronovitch K, Brener A, Levek N, Levran N, Chorna E, Dekel M, Barash G, Landau Z, Pinhas-Hamiel O.	Israel	Asia	Article	2021	Diabetes mellitus type I	Metabolic diseases	Observational studies	Acta Diabetol. 2021 Jun58(6):697-705	ACTA DIABETOLOGICA	4,28	ENDOCRINOLOGY & METABOLISM	9	Trearment	10.1007/s00592-021-01673-2
Telemedicine to enhance pediatric diabetes care: Video consultations as an extension of outpatient diabetes care	von Sengbusch S., Frielitz F.S., Braune K., Boss K., Raile K.	Germany	Europe	Article	2021	Diabetes mellitus type I	Metabolic diseases	Review/meta-analysis	Diabetologe (2021) 17:6 (638-646)	DIABETOLOGE	0,421	ENDOCRINOLOGY & METABOLISM	0	Follow up	10.1007/s11428-021-00758-4
Effect of mobile health based peripartum management of gestational diabetes mellitus on postpartum diabetes: A randomized controlled trial	Huang F, Zhang S, Tian Y, Li L, Li Y, Chen X, Sun X, Fan Y, Ma W, Liu C, Gao L, Xue X, Ma L.	China	Asia	Article	2021	Diabetes mellitus type I	Metabolic diseases	Interventional studies	Diabetes Res Clin Pract. 2021 May175:108775	DIABETES RESEARCH AND CLINICAL PRACTICE	5,602	ENDOCRINOLOGY & METABOLISM	0	Trearment	10.1016/j.diabres.2021.108775

COVID-19 pandemic lockdown in young people with type 1 diabetes: Positive results of an unprecedented challenge for patients through telemedicine and change in use of continuous glucose monitoring	Salabelle C, Ly Sall K, Eroukhmanoff J, Franc S, Oumbiche H, Zrafi WS, Dang Duy TL, Valentim C, Gaston F, Fernandes S, Faucherand M, Penfornis A, Amadou C.	France	Europe	Article	2021	Diabetes mellitus type I	Metabolic diseases	Review/meta-analysis	Prim Care Diabetes. 2021 Oct;15(5):884-886	PRIMARY CARE DIABETES	2,459	ENDOCRINOLOGY & METABOLISM	3	Follow up	10.1016/j.pcd.2021.06.013
Assessing the benefits and challenges of video consultations for the treatment of children with type 1 diabetes - A qualitative study among diabetes professionals	Frielitz F.-S., Dördelmann J., Lemke S., Lange K., Hiort O., Katalinic A., Von Sengbusch S.	Germany	Europe	Article	2021	Diabetes mellitus type I	Metabolic diseases	Interventional studies	Experimental and Clinical Endocrinology and Diabetes (2021) 129:11 (831-836)	EXPERIMENTAL AND CLINICAL ENDOCRINOLOGY AND DIABETES	2,949	ENDOCRINOLOGY & METABOLISM	0	Trearment	10.1055/a-1149-8814
The Effectiveness of Virtual Training on the MiniMed™ 670G System in People with Type 1 Diabetes During the COVID-19 Pandemic	Vigersky RA, Velado K, Zhong A, Agrawal P, Cordero TL.	United States of America	North America	Article	2021	Diabetes mellitus type I	Metabolic diseases	Observational studies	Diabetes Technol Ther. 2021 Feb23(2):104-109	DIABETES TECHNOLOGY & THERAPEUTICS	6,118	ENDOCRINOLOGY & METABOLISM	24	Trearment	10.1089/dia.2020.0234
Youth with Type 1 Diabetes Had Improvement in Continuous Glucose Monitoring Metrics During the COVID-19 Pandemic	Abdulhussein, FS; Chesser, H; Boscardin, WJ; Gitelman, SE; Wong, JC	United States of America	North America	Article	2021	Diabetes mellitus type I	Metabolic diseases	Observational studies	Diabetes Technol Ther. 2021 Oct;23(10):684-691	DIABETES TECHNOLOGY & THERAPEUTICS	6,118	ENDOCRINOLOGY & METABOLISM	1	Follow up	10.1089/dia.2021.0131
Influence of Telemedicine on the Number of Visits and HbA1c Determinations in Latin American Children with Type 1 Diabetes	Hirschler V., Molinari C., Figueroa Sobrero A., Pelicand J., Pinto Ibárcena P., Del Aguila Villar C.M., Scaiola E., Bocco P., Gonzalez D.S., Mac A., Ramirez Trillo C., Mora Brito E., Acosta J., Lapertosa S., Gonzalez C.D.	Argentina	South America	Article	2021	Diabetes mellitus type I	Metabolic diseases	Review/meta-analysis	Diabetes Technology and Therapeutics (2021) 23:11 (731-736)	DIABETES TECHNOLOGY & THERAPEUTICS	6,118	ENDOCRINOLOGY & METABOLISM	0	Follow up	10.1089/dia.2021.0189
Challenges to telemedicine transition during COVID-19; insights from 21 us diabetes and endocrinology clinics	Lee J., Carlson E., Demeterco-Berggren C., Corathers S., Jimenez-Vega J., Vendrame F., Weinstock R., Ebekozien O.	United States of America	North America	Conference abstract	2021	Diabetes mellitus type I	Metabolic diseases	Observational studies	Diabetes Technology and Therapeutics (2021) 23:SUPPL 2 (A45-A46)	DIABETES TECHNOLOGY & THERAPEUTICS	6,118	ENDOCRINOLOGY & METABOLISM	0	Diagnosis & Treatment/Follow-up	10.1089/dia.2021.2525.abstracts
Telemedicine in paediatric diabetes-patient and clinicians' view and response to remote consultations during COVID-19	Clarke E., Princy P., Deakin M., Mehta F., Millar K., Simmons A., Ghatak A.	United Kingdom	Europe	Conference abstract	2021	Diabetes mellitus type I	Metabolic diseases	Case report/ case series	Diabetes Technology and Therapeutics (2021) 23:SUPPL 2 (A133-A134)	DIABETES TECHNOLOGY & THERAPEUTICS	6,118	ENDOCRINOLOGY & METABOLISM	0	Follow up	10.1089/dia.2021.2525.abstracts

COVID-19 pandemic lockdown effect in adolescents and young adults with type 1 diabetes: Positive results of an unprecedented challenge for telemedicine and patient self-management	Salabelle C., Ly Sall K., Eroukhmanoff J., Franc S., Dang Duy T.-L., Amadou C.	France	Europe	Conference abstract	2021	Diabetes mellitus type I	Metabolic diseases	Observational studies	Diabetes Technology and Therapeutics (2021) 23:SUPPL 2 (A134-A135)	DIABETES TECHNOLOGY & THERAPEUTICS	6,118	ENDOCRINOLOGY & METABOLISM	0	Follow up	10.1089/dia.2021.2525.abstracts
Transition to remote diabetes care in COVID-19 times: Experiences from a specialized type 1 diabetes clinic	Nefs G., Winterdijk P., Dekker P., De Vries M., Sas T., Mul D., Veeze H., Aanstoot H.-J.	Netherland	Europe	Conference abstract	2021	Diabetes mellitus type I	Metabolic diseases	Observational studies	Diabetes Technology and Therapeutics (2021) 23:SUPPL 2 (A138)	DIABETES TECHNOLOGY & THERAPEUTICS	6,118	ENDOCRINOLOGY & METABOLISM	0	Follow up	10.1089/dia.2021.2525.abstracts
Parental concerns of COVID-19 and pediatric diabetes	Udo E., Shah A., Yafi M.	United States of America	North America	Conference abstract	2021	Diabetes mellitus type I	Metabolic diseases	Observational studies	Diabetes Technology and Therapeutics (2021) 23:SUPPL 2 (A182)	DIABETES TECHNOLOGY & THERAPEUTICS	6,118	ENDOCRINOLOGY & METABOLISM	0	Follow up	10.1089/dia.2021.2525.abstracts
Optimizing workflows to close disparities in telehealth use	Prahalad P., Leverenz B., Freeman A., Grover M., Shah S., Conrad B., Stafford D., Maahs D.	United States of America	North America	Conference abstract	2021	Diabetes mellitus type I	Metabolic diseases	Observational studies	Diabetes Technology and Therapeutics (2021) 23:SUPPL 2 (A43-A44)	DIABETES TECHNOLOGY & THERAPEUTICS	6,118	ENDOCRINOLOGY & METABOLISM	0	Follow up	10.1089/dia.2021.2525.abstracts
Intervening on hypoglycemia fear in parents of young children using direct-tohome video-based telehealth	Patton S.	United States of America	North America	Conference abstract	2021	Diabetes mellitus type I	Metabolic diseases	Interventional studies	Diabetes Technology and Therapeutics (2021) 23:SUPPL 2 (A8-A9)	DIABETES TECHNOLOGY & THERAPEUTICS	6,118	ENDOCRINOLOGY & METABOLISM	0	Follow up	10.1089/dia.2021.2525.abstracts
Direct-to-consumer telehealth to support youth with type 1 diabetes (T1D) predicted to experience a rise in hemoglobin A1c (A1c): A pragmatic trial	Dewit E., Williams D., Patton S., Mullaney C., Ferro D., Noland K., Skrabonja L., Spartz B., Elliott R., Kenyon R., Mcdonough R., Mehta S., D'Avolio L., Clements M.	United States of America	North America	Conference abstract	2021	Diabetes mellitus type I	Metabolic diseases	Interventional studies	Diabetes Technology and Therapeutics (2021) 23:SUPPL 2 (A48)	DIABETES TECHNOLOGY & THERAPEUTICS	6,118	ENDOCRINOLOGY & METABOLISM	0	Trearment	10.1089/dia.2021.2525.abstracts
Exclusive telemedicine during covid pandemic: what we learn about its impact on diabetes metabolic control, care and satisfaction of families in our Chilean rural-urban center?	Pelicand J., Muñoz A.A., Muñoz S.Z., Ordonez C.N., Ponce M.C., Vásquez C.C.	Chile	South America	Conference abstract	2021	Diabetes mellitus type I	Metabolic diseases	Observational studies	Diabetes Technology and Therapeutics (2021) 23:SUPPL 2 (A146)	DIABETES TECHNOLOGY & THERAPEUTICS	6,118	ENDOCRINOLOGY & METABOLISM	0	Follow up	10.1089/dia.2021.2525.abstracts Entry date 2021-07-16 (Full record)

Remote patient monitoring for youth with type 1 diabetes (T1D) predicted to experience a rise in hemoglobin A1C (A1C)	Williams D.D., Ferro D., Lockee B., Carrothers S., Barnes M.S., DeWit E., Albert S., Spartz B., McDonough R.J., Clements M.A.	United States of America	North America	Conference abstract	2021	Diabetes mellitus type I	Metabolic diseases	Interventional studies	Journal of Diabetes (2021) 13:SUPPL 1 (17)	JOURNAL OF DIABETES	4,006	ENDOCRINOLOGY & METABOLISM	0	Follow up	10.1111/1753-0407.13227
Parental expectations before and after 12-month experience with video consultations combined with regular outpatient care for children with type 1 diabetes: a qualitative study	von Sengbusch S., Doerdelmann J., Lemke S., Lange K., Hiort O., Katalinic A., Frielitz F.S.	Germany	Europe	Article	2021	Diabetes mellitus type I	Metabolic diseases	Interventional studies	Diabetic Medicine (2021) 38:6	DIABETIC MEDICINE	4,359	ENDOCRINOLOGY & METABOLISM	7	Follow up	10.1111/dme.14410
The automated pancreas: A review of technologies and clinical practice	Biester T., Tauschmann M., Chobot A., Kordonouri O., Danne T., Kapellen T., Dovc K.	Germany	Europe	Article	2021	Diabetes mellitus type I	Metabolic diseases	Review/meta-analysis	Diabetes, Obesity and Metabolism (2021)	DIABETES, OBESITY AND METABOLISM	6,577	ENDOCRINOLOGY & METABOLISM	1	Diagnosis & Treatment/Follow-up	10.1111/dom.14576
Telemedicine, health disparities and seizure control in pediatric epilepsy during the COVID-19 pandemic	Kaufman M., Fitzgerald M., Massey S., Fridinger S., Prelack M., Elis C., Ortiz-Gonzalez X., Fried L., DiGiovine M., Melamed S., Malcolm M., Banwell B., Stephenson D., Witzman S., Gonzalez A., Dlugos D., Kessler S., Goldberg E., Abend N., Helbig I.	United States of America	North America	Conference abstract	2021	Epilepsy	Brain disorders	Observational studies	Epilepsia (2021) 62:SUPPL 3 (25)	EPILEPSIA	5,866	ENDOCRINOLOGY & METABOLISM	0	Follow up	10.1111/epi.17079
Attitude towards Telemedicine use in patients with epilepsy during the pandemic COVID-19 in Mexico, according to a survey	Gonzalez Villagomez E.M., Santos-Peyret A., Thompson A.	Mexico	South America	Conference abstract	2021	Epilepsy	Brain disorders	Observational studies	Epilepsia (2021) 62:SUPPL 3 (320-321)	EPILEPSIA	5,866	ENDOCRINOLOGY & METABOLISM	0	Trearment	10.1111/epi.17079
Glycemic control in children and teenagers with type 1 diabetes around lockdown for COVID-19: A continuous glucose monitoring-based observational study	Wu X, Luo S, Zheng X, Ding Y, Wang S, Ling P, Yue T, Xu W, Yan J, Weng J.	China	Asia	Article	2021	Diabetes mellitus type I	Metabolic diseases	Observational studies	J Diabetes Investig. 2021 Sep12(9):1708-1717	JOURNAL OF DIABETES INVESTIGATION	4,232	ENDOCRINOLOGY & METABOLISM	9	Follow up	10.1111/jdi.13519
Predictors of engagement in clinical care: Coyot1 to California	Garcia J.J.F., Reid M.W., Pyatak E., Fox D.S., Fogel J.L., Salcedo-Rodriguez E., Bisno D.I., Miller D., Mittal A., Raymond J.	United States of America	North America	Conference abstract	2021	Diabetes mellitus type I	Metabolic diseases	Interventional studies	Diabetes (2021) 70:SUPPL 1	DIABETES	9,461	ENDOCRINOLOGY & METABOLISM	0	Diagnosis	10.2337/db21-537-P
Assessing group engagement during parent-focused telehealth interventions in pediatric type 1 diabetes	Monzon A., Clements M.A., Patton S.R.	United States of America	North America	Conference abstract	2021	Diabetes mellitus type I	Metabolic diseases	Interventional studies	Diabetes (2021) 70:SUPPL 1	DIABETES	9,461	ENDOCRINOLOGY & METABOLISM	1	Trearment	10.2337/db21-568-P

Telemedicine in the COVID-19 era: Taking care of children with obesity and diabetes mellitus	Umano G.R., Di Sessa A., Guarino S., Gaudino G., Marzuillo P., Giudice E.M.D.	Italy	Europe	Article	2021	Diabetes mellitus type I	Metabolic diseases	Review/meta-analysis	World Journal of Diabetes (2021) 12:5 (651-657)	WORLD JOURNAL OF DIABETES	3,763	ENDOCRINOLOGY & METABOLISM	0	Diagnosis & Treatment/Follow-up	10.4239/wjd.v12.i5.651
Telemedicine and urban diabetes during COVID-19 pandemic in Milano, Italy during lock-down: epidemiological and sociodemographic picture	Luzi L., Carruba M., Crialesi R., Da Empoli S., Dagani R., Lovati E., Nicolucci A., Berra C.C., Cipponeri E., Vaccaro K., Lenzi A.	Italy	Europe	Article	2021	Diabetes mellitus type I	Metabolic diseases	Observational studies	Acta Diabetologica (2021) 58:7 (919-927)	ACTA DIABETOLOGICA	4,28	ENDOCRINOLOGY & METABOLISM	5	Trearment	10.1007/s00592-021-01700-2
Telemedicine to enhance pediatric diabetes care Video consultations as an extension of outpatient diabetes care	von Sengbusch, S; Frielitz, FS; Braune, K; Boss, K; Raile, K	Germany	Europe	Article	2021	Diabetes mellitus type I	Metabolic diseases	Review/meta-analysis	Diabetologe 17, 638–646 (2021).	DIABETOLOGE	0,421	ENDOCRINOLOGY & METABOLISM	0	Trearment	10.1007/s11428-021-00758-4
Automated insulin delivery (AID) systems in diabetology	Biester, T; Dovc, K; Chobot, A; Tauschmann, M; Kapellen, T	Germany	Europe	Article	2021	Diabetes mellitus type I	Metabolic diseases	Review/meta-analysis	Diabetologe, 2021;30 (627 - 637)	DIABETOLOGE	0,421	ENDOCRINOLOGY & METABOLISM	3	Trearment	10.1007/s11428-021-00777-1
Has COVID-19 lockdown improved glycaemic control in pediatric patients with type 1 diabetes? An analysis of continuous glucose monitoring metrics	Lombardo F, Salzano G, Bombaci B, Basile P, Lucania G, Alibrandi A, Passanisi S.	Italy	Europe	Article	2021	Diabetes mellitus type I	Metabolic diseases	Observational studies	Diabetes Res Clin Pract. 2021 Aug178:108988	DIABETES RESEARCH AND CLINICAL PRACTICE	5,602	ENDOCRINOLOGY & METABOLISM	0	Follow up	10.1016/j.diabres.2021.108988
A Scoping Review and General User's Guide for Facilitating the Successful Use of eHealth Programs for Diabetes in Clinical Care	Fisher L, Glasgow RE, Huebschmann A.	United States of America	North America	Article	2021	Diabetes mellitus type I	Metabolic diseases	Review/meta-analysis	Diabetes Technol Ther. 2021 Feb;23(2):133-145	DIABETES TECHNOLOGY & THERAPEUTICS	6,118	ENDOCRINOLOGY & METABOLISM	2	Trearment	10.1089/dia.2020.0383
Improved Estimation of Glycated Hemoglobin from Continuous Glucose Monitoring and Past Glycated Hemoglobin Data	Chrzanowski J, Michalak A, Łosiewicz A, Kuśmierczyk H, Mianowska B, Szadkowska A, Fendler W.	Poland	Europe	Article	2021	Diabetes mellitus type I	Metabolic diseases	Interventional studies	Diabetes Technol Ther. 2021 Apr23(4):293-305	DIABETES TECHNOLOGY & THERAPEUTICS	6,111	ENDOCRINOLOGY & METABOLISM	2	Follow up	10.1089/dia.2020.0433
A Virtual Training Program for the Tandem t:slim X2 Insulin Pump: Implementation and Outcomes	Pinsker JE, Singh H, McElwee Malloy M, Constantin A, Leas S, Kriegel K, Habif S.	United States of America	North America	Article	2021	Diabetes mellitus type I	Metabolic diseases	Interventional studies	Diabetes Technol Ther. 2021 Jun23(6):467-470	DIABETES TECHNOLOGY & THERAPEUTICS	6,118	ENDOCRINOLOGY & METABOLISM	3	Follow up	10.1089/dia.2020.0602
Adoption of Telemedicine for Type 1 Diabetes Care During the COVID-19 Pandemic	Lee JM, Carlson E, Albanese-O'Neill A, Demeterco-Berggren C, Corathers SD, Vendrame F, Weinstock RS, Prahalad P, Alonso GT, Kamboj M, DeSalvo DJ, Malik FS,	United States of America	North America	Article	2021	Diabetes mellitus type I	Metabolic diseases	Observational studies	Diabetes Technol Ther. 2021 Sep23(9):642-651	DIABETES TECHNOLOGY & THERAPEUTICS	6,118	ENDOCRINOLOGY & METABOLISM	4	Follow up	10.1089/dia.2021.0080

	Izquierdo R, Ebekozien O.														
Increasing capacity with fixed resources through automation and task delegation at a tertiary pediatric type 1 diabetes clinic	Vitko A., Osmanlliu E., Johari R., Prahalad P., Maahs D., Scheinker D.	United States of America	North America	Conference abstract	2021	Diabetes mellitus type I	Metabolic diseases	Observational studies	Diabetes Technology and Therapeutics (2021) 23:SUPPL 2 (A139-A140)	DIABETES TECHNOLOGY & THERAPEUTICS	6,118	ENDOCRINOLOGY & METABOLISM	0	Follow up	10.1089/dia.2021.2525.abstracts
Covidentary 2 tele-exercise program: An innovative instrument to implement physical activity in children and adolescents with type 1 diabetes	Calcaterra V., Vandoni M., Carnevale Pellino V., Mameli C., Tornese G., Macedoni M., Redaelli F., Cascella C., Chianese A., Zuccotti G.V., Iafusco D.	Italy	Europe	Conference abstract	2021	Diabetes mellitus type I	Metabolic diseases	Interventional studies	Diabetes Technology and Therapeutics (2021) 23:SUPPL 2 (A69-A70)	DIABETES TECHNOLOGY & THERAPEUTICS	6,118	ENDOCRINOLOGY & METABOLISM	0	Trearment	10.1089/dia.2021.2525.abstracts
Epilepsy care during the COVID-19 pandemic	Cross, JH; Kwon, CS; Asadi-Pooya, AA; Balagura, G; Gomez-Iglesias, P; Guekht, A; Hall, J; Ikeda, A; Kishk, NA; Murphy, P; Kissani, N; Naji, Y; Perucca, E; Perez-Poveda, JC; Sanya, EO; Trinka, E; Zhou, D; Wiebe, S; Jette, N	United Kingdom	Europe	Article	2021	Epilepsy	Brain disorders	Observational studies	Epilepsia 2021 62:10 (2322-2332)	EPILEPSIA	5,866	ENDOCRINOLOGY & METABOLISM	8	Follow up	10.1111/epi.17045
Predictors of engagement in clinical care: Coyot1 to California	Garcia J.J.F., Reid M.W., Pyatak E., Fox D.S., Fogel J.L., Salcedo-Rodriguez E., Bisno D.I., Miller D., Mittal A., Raymond J.	United States of America	North America	Conference abstract	2021	Diabetes mellitus type I	Metabolic diseases	Interventional studies	Diabetes (2021) 70:SUPPL 1	DIABETES	9,461	ENDOCRINOLOGY & METABOLISM	0	Diagnosis	10.2337/db21-537-P
The Rapid Transition to Telemedicine and Its Effect on Access to Care for Patients With Type 1 Diabetes During the COVID-19 Pandemic	Tilden DR, Datye KA, Moore DJ, French B, Jaser SS.	United States of America	North America	Article	2021	Diabetes mellitus type I	Metabolic diseases	Observational studies	Diabetes Care. 2021 Jun44(6):1447-1450	DIABETES CARE	19,112	ENDOCRINOLOGY & METABOLISM	10	Follow up	10.2337/dc20-2712
The Effect of Lockdown and Physical Activity on Glycemic Control in Italian Children and Young Patients With Type 1 Diabetes	Minuto N, Bassi M, Montobbio C, Vinci F, Mercuri C, Perri FN, Cabri M, Calevo MG, d'Annunzio G, Maghnie M.	Italy	Europe	Article	2021	Diabetes mellitus type I	Metabolic diseases	Observational studies	Front Endocrinol (Lausanne). 2021 Jul 1312:690222	FRONTIERS IN ENDOCRINOLOGY (LAUSANNE)	5,555	ENDOCRINOLOGY & METABOLISM	4	Trearment	10.3389/fendo.2021.690222
TELEMEDICINE IN PAEDIATRIC DIABETES - PATIENT AND CLINICIANS' VIEW AND RESPONSE TO REMOTE CONSULTATIONS DURING COVID-19	Clarke, E; Princy, P; Deakin, M; Mehta, F; Millar, K; Simmons, A; Ghatak, A	United Kingdom	Europe	Conference abstract	2021	Diabetes mellitus type I	Metabolic diseases	Observational studies	Diabetes Technology & Therapeutics.Jun 2021.A-1-A-206.	DIABETES TECHNOLOGY & THERAPEUTICS	6,118	ENDOCRINOLOGY & METABOLISM	0	Follow up	NA

What's the Name of the Game? The Impact of eHealth on Productive Interactions in Chronic Care Management	Wannheden, C; Schwarz, UV; Ostenson, CG; Harenstam, KP; Stenfors, T	Sweden	Europe	Article	2021	Diabetes mellitus type I	Metabolic diseases	Observational studies	NA	SUSTAINABILITY	3,251	ENVIRONMENTAL STUDIES	0	Follow up	https://doi.org/10.3390/su13095221
A meta-analysis on the effect of telemedicine on the management of attention deficit and hyperactivity disorder in children and adolescents	Bemanalizadeh M., Yazdi M., Yaghini O., Kelishadi R.	Iran	Asia	Article	2021	Conduct disorder	Neurodevelopmental diseases or disorders	Review/meta-analysis	Journal of telemedicine and telecare (2021) (1357633X211045186)	JOURNAL OF TELEMEDICINE AND TELECARE	6,184	HEALTH CARE SCIENCES & SERVICES	0	Trearment	10.1177/1357633X211045186
Group engagement in parent-focused telehealth interventions for families of children with type 1 diabetes	Monzon, AD; Clements, MA; Patton, SR	United States of America	North America	Article	2021	Diabetes mellitus type I	Metabolic diseases	Interventional studies	J Telemed Telecare. 2021 Dec 21:1357633X211067074	JOURNAL OF TELEMEDICINE AND TELECARE	6,184	HEALTH CARE SCIENCES & SERVICES	0	Follow up	10.1177/1357633X211067074
Evaluating the Financial Sustainability of the School-Based Telemedicine Asthma Management Program	Crabtree-Ide C., Lillvis D.F., Nie J., Fagnano M., Tajon R.S., Tremblay P., Halterman J.S., Noyes K.	United States of America	North America	Article	2021	Asthma	Asthma	Interventional studies	Population Health Management (2021) 24:6 (664-674)	POPULATION HEALTH MANAGEMENT	2,459	HEALTH CARE SCIENCES & SERVICES	0	Trearment	10.1089/pop.2020.0361
Validity of Telemedicine for Diagnosing Autism Spectrum Disorder: Protocol-Guided Video Recording Evaluation	Sutantio JD, Pusponegoro HD, Sekartini R.	Indonesia	Asia	Article	2021	Autism spectrum disorders	Neurodevelopmental diseases or disorders	Observational studies	Telemed J E Health. 2021 Apr27(4):427-431	TELEMEDICINE AND E-HEALTH	3,536	HEALTH CARE SCIENCES & SERVICES	3	Diagnosis	10.1089/tmj.2020.0035
Impact of Pediatric Electronic Consultations in a Federally Qualified Health Center	Porto A, Rubin K, Wagner K, Chang W, Macri G, Anderson D.	United States of America	North America	Article	2021	Asthma	Asthma	Observational studies	Telemed J E Health. 2021 Dec27(12):1379-1384	TELEMEDICINE AND E-HEALTH	3,536	HEALTH CARE SCIENCES & SERVICES	1	Diagnosis & Treatment/Follow-up	10.1089/tmj.2020.0394
Nurse Perspectives Regarding Implementation of an Asthma Monitoring Mobile Health Application in the School Setting	Johnson EE, MacGeorge C, Andrews A, King KL, Teufel RJ, Brinton DL, Kruis R, Hale KC, Ford D, Sterba KR.	United States of America	North America	Article	2021	Asthma	Asthma	Observational studies	Telemed J E Health. 2021 Aug27(8):955-962	TELEMEDICINE AND E-HEALTH	3,536	HEALTH CARE SCIENCES & SERVICES	0	Follow up	10.1089/tmj.2021.0100
Caregiver's Opinions on the Design of the Screens of a Future Gamified Mobile Application for Self-Management of Type 1 Diabetes in Children in Saudi Arabia	Alsalman, D.M., Ali, Z.B., Alnosaier, Z., Alotaibi, N., Alanzi, T.M.	Saudi Arabia	Asia	Article	2021	Diabetes mellitus type I	Metabolic diseases	Observational studies	International Journal of Telemedicine and Applications,2021,8822676	INTERNATIONAL JOURNAL OF TELEMEDICINE AND APPLICATIONS	NA	HEALTH CARE SCIENCES & SERVICES	0	Follow up	10.1155/2021/8822676
Parent Ratings of Generalized and Indirect Effects of Functional Communication Training for Children with Autism Spectrum Disorder	O'Brien M.J., Pelzel K.E., Hendrix N.M., Schieltz K.M., Miller K., Call N.A., Tsami L., Lerman D.C., Berg W.K., Kopelman T.G.,	United States of America	North America	Article	2021	Autism spectrum disorders	Neurodevelopmental diseases or disorders	Interventional studies	Behavior modification (2021) (01454455211018815)	BMC HEALTH SERVICES RESEARCH	2,655	HEALTH CARE SCIENCES & SERVICES	1	Trearment	10.1177/01454455211018815

	Wacker D.P., Lindgren S.D.														
Formative Evaluation for Implementation of a Low Literacy Pictorial Asthma Action Plan Delivered via Telehealth Improves Asthma Control	Vallabhan MK, Jimenez EY, McCauley GL, Willyard H, Kong AS.	United States of America	North America	Article	2021	Asthma	Asthma	Observational studies	Am J Med Qual. 2021 Jul-Aug 0136(4):229-237	AMERICAN JOURNAL OF MEDICAL QUALITY	1,852	HEALTH CARE SCIENCES & SERVICES	0	Follow up	10.1177/106286062094683 8
Development and preliminary results of an Electronic Medical Record (EMR)-integrated smartphone telemedicine program to deliver asthma care remotely	Mammen JR, Java JJ, Halterman J, Berliant MN, Crowley A, Frey SM, Reznik M, Feldman JM, Schoonmaker JD, Arcoleo K.	United States of America	North America	Article	2021	Asthma	Asthma	Interventional studies	J Telemed Telecare. 2021 May27(4):217-230	JOURNAL OF TELEMEDICIN E AND TELECARE	6,184	HEALTH CARE SCIENCES & SERVICES	6	Treament	10.1177/1357633X1987002 5
Going virtual: youth attitudes toward and experiences of virtual mental health and substance use services during the COVID-19 pandemic	Hawke, LD; Sheikhan, NY; MacCon, K; Henderson, J	Canada	North America	Article	2021	Anxiety disorders	Mental disorders	Observational studies	BMC Health Serv Res. 2021;21(1):340	BMC HEALTH SERVICES RESEARCH	2,655	HEALTH CARE SCIENCES & SERVICES	3	Follow up	10.1186/s12913-021-06321-7
ECHO Autism: Evaluation of Participants' Perceptions of Collaborative Telementoring Network	Becevic, M; Nair, P; Wallach, E; Hoffman, K; Sohl, K	United States of America	North America	Article	2021	Autism spectrum disorders	Neurodev elopmenta l diseases or disorders	Observational studies	J Patient Exp. 2021 Dec 20;8:2374373521 1065292	JOURNAL OF PATIENT EXPERIENCE	0,53	HEALTH CARE SCIENCES & SERVICES	0	Treament	10.1177/237437352110652 92
Smartphone App for monitoring Asthma in children and adolescents	Mayoral K, Garin O, Caballero-Rabasco MA, Praena-Crespo M, Bercedo A, Hernandez G, Castillo J, Lizano Barrantes C, Pardo Y, Ferrer M	Spain	Europe	Article	2021	Asthma	Asthma	Study protocol/Pilot study	Qual Life Res. 2021 Nov;30(11):3127-3144	QUALITY OF LIFE RESEARCH	4,147	HEALTH POLICY & SERVICES	6	Follow up	10.1007/s11136-020-02706-z
A Systematic Review and Meta-Analysis of Change in Health-Related Quality of Life for Interactive Telehealth Interventions for Patients With Asthma	Snoswell C.L., Rahja M., Lalor A.F.	Australia	Oceania	Article	2021	Asthma	Asthma	Review/meta-analysis	Value in Health (2021) 24:2 (291-302)	VALUE IN HEALTH	5,728	HEALTH POLICY & SERVICES	2	Follow up	10.1016/j.jval.2020.09.006
Telemedicine in the Time of the COVID-19 Pandemic: Results from the First Survey among Italian Pediatric Diabetes Centers	Tornese, G; Schiaffini, R; Mozzillo, E; Franceschi, R; Frongia, A; Scaramuzza, A	Italy	Europe	Article	2021	Diabetes mellitus type I	Metabolic diseases	Observational studies	Healthcare (Switzerland),9(7),815	HEALTHCARE (SWITZERLAN D)	2,656	HEALTH POLICY & SERVICES	7	Follow up	10.3390/healthcare9070815
Shaping Workflows in Digital and Remote Diabetes Care During the COVID-19 Pandemic via Service Design: Prospective, Longitudinal,	Braune K, Boss K, Schmidt-Herzel J, Gajewska KA, Thieffry A, Schulze L, Posern B, Raile K.	Germany	Europe	Article	2021	Diabetes mellitus type I	Metabolic diseases	Interventional studies	JMIR Mhealth Uhealth. 2021 Apr 59(4):e24374	JMIR mHEALTH AND uHEALTH	4,773	MEDICAL INFORMATICS	6	Treament	10.2196/24374

Open-label Feasibility Trial															
Implementation of Telemental Health Services Before COVID-19: Rapid Umbrella Review of Systematic Reviews	Barnett, P; Goulding, L; Casetta, C; Jordan, H; Sheridan-Rains, L; Steare, T; Williams, J; Wood, L; Gaughran, F; Johnson, S	United Kingdom	Europe	Article	2021	Anxiety disorders	Mental disorders	Review/meta-analysis	J Med Internet Res. 2021;23(7):e26492	JOURNAL OF MEDICAL INTERNET RESEARCH	5,428	MEDICAL INFORMATICS	5	Diagnosis & Treatment/Follow-up	10.2196/26492
Mobile applications for emerging adults transitioning to independent diabetes monitoring	Schindler-Ruwisch J, Peters A.	United States of America	North America	Article	2021	Diabetes mellitus type I	Metabolic diseases	Review/meta-analysis	Inform Health Soc Care. 2021 Mar 246(1):56-67	INFORMATICS FOR HEALTH AND SOCIAL CARE	2,439	MEDICAL INFORMATICS	0	Follow up	10.1080/17538157.2020.1837839
An mHealth-Based Intervention for Adolescents With Type 1 Diabetes and Their Parents: Pilot Feasibility and Efficacy Single-Arm Study	Holtz B, Mitchell KM, Holmstrom AJ, Cotten SR, Dunneback JK, Jimenez-Vega J, Ellis DA, Wood MA.	United States of America	North America	Article	2021	Diabetes mellitus type I	Metabolic diseases	Interventional studies	JMIR Mhealth Uhealth. 2021 Sep 149(9):e23916	JMIR mHEALTH AND uHEALTH	4,773	MEDICAL INFORMATICS	1	Treatment	10.2196/23916
Online Mindfulness-Based Cognitive Behavioral Therapy Intervention for Youth With Major Depressive Disorders: Randomized Controlled Trial	Ritvo P, Knyahnytska Y, Pirbaglou M, Wang W, Tomlinson G, Zhao H, Linklater R, Bai S, Kirk M, Katz J, Harber L, Daskalakis Z.	United States of America	North America	Article	2021	Conduct disorder	Neurodevelopmental diseases or disorders	Interventional studies	J Med Internet Res. 2021 Mar 1023(3):e24380	JOURNAL OF MEDICAL INTERNET RESEARCH	5,428	MEDICAL INFORMATICS	4	Treatment	10.2196/24380
Effective German and English Language mHealth Apps for Self-management of Bronchial Asthma in Children and Adolescents: Comparison Study	Franzmair J, Diesner-Treiber SC, Voitl JJM, Voitl P.	Austria	Europe	Article	2021	Asthma	Asthma	Review/meta-analysis	JMIR Mhealth Uhealth. 2021 May 199(5):e24907	JMIR mHEALTH AND uHEALTH	4,773	MEDICAL INFORMATICS	1	Follow up	10.2196/24907
TELEmedicine for EPIlepsy Care (TELE-EPIC): Protocol of a randomised, open controlled non-inferiority clinical trial	Licchetta L., Trivisano M., Baldin E., Mohamed S., Raschi E., Mostacci B., Zenesini C., Contin M., Vigevano F., Bisulli F., Tinuper P., Vignatelli L.	Italy	Europe	Article	2021	Epilepsy	Brain disorders	Interventional studies	BMJ Open (2021) 11:12	BMJ OPEN	2,692	MEDICINE, GENERAL & INTERNAL	0	Follow up	10.1136/bmjopen-2021-053980
Efficacy of parent-mediated communication-focused treatment in toddlers with autism (PACT) delivered via videoconferencing: a randomised controlled trial study protocol	Jurek L, Occelli P, Denis A, Amestoy A, Maffre T, Dauchez T, Oreve MJ, Baghdadli A, Schroder C, Jay A, Zelmar A, Revah-Levy A, Gallifet N, Aldred C, Garg S, Green J, Touzet S, Geoffray MM	France	Europe	Article	2021	Autism spectrum disorders	Neurodevelopmental diseases or disorders	Interventional studies	BMJ Open. 2021 Apr 7;11(4):e044669	BMJ OPEN	2,692	MEDICINE, GENERAL & INTERNAL	0	Treatment	10.1136/bmjopen-2020-044669

Internet-assisted cognitive behavioural therapy with telephone coaching for anxious Finnish children aged 10-13 years: study protocol for a randomised controlled trial	Luntamo T, Korpilahti-Leino T, Ristkari T, Hinkka-Yli-Salomäki S, Kurki M, Sinokki A, Lamminen K, Saanakorpi K, Saarinen S, Maunuksela M, Sourander S, Toivonen K, Zadkova A, Suilamo M, Casagrande L, Palmroth J, Sourander A.	Finland	Europe	Article	2021	Anxiety disorders	Mental disorders	Interventional studies	BMJ Open. 2021 Jun 2311(6):e045474	BMJ OPEN	2,692	MEDICINE, GENERAL & INTERNAL	0	Diagnosis & Treatment/Follow-up	10.1136/bmjopen-2020-045474
Coaching via telehealth: Caregiver-mediated interventions for young children on the waitlist for an autism diagnosis using single-case design	Kunze M.G., Machalicek W., Wei Q., Joseph S.St.	United States of America	North America	Article	2021	Autism spectrum disorders	Neurodevelopmental diseases or disorders	Interventional studies	Journal of Clinical Medicine (2021) 10:8	JOURNAL OF CLINICAL MEDICINE	4,242	MEDICINE, GENERAL & INTERNAL	2	Trearment	10.3390/jcm10081654
Emerging Needs and Viability of Telepsychiatry During and Post COVID-19 Era: A Literature Review	Gude, J; Subhedar, RV; Zhang, MH; Jain, P; Bhela, J; Bangash, F; Veluri, N; Hsieh, YC; Sheikh, BZ; Shah, MR; Mansuri, Z; Aedma, K; Patel, UK; Parikh, T	United States of America	North America	Article	2021	Major depressive disorder	Mental disorders	Review/meta-analysis	Cureus. 2021 Aug 7;13(8):e16974.	CUREUS	NA	MEDICINE, GENERAL & INTERNAL	0	Trearment	10.7759/cureus.16974
The primary care assessment and research of a telephone intervention for neuropsychiatric conditions with education and resources study: Design, rationale, and sample of the PARTNERs randomized controlled trial	Rodie, DJ; Fitzgibbon, K; Perivolaris, A; Crawford, A; Geist, R; Levinson, A; Mitchell, B; Oslin, D; Sunderji, N; Mulsant, BH	Canada	North America	Article	2021	Major depressive disorder	Mental disorders	Interventional studies	Contemp Clin Trials. 2021 Apr;103:106284	CONTEMPORARY CLINICAL TRIALS	2,226	MEDICINE, RESEARCH & EXPERIMENTAL	0	Trearment	10.1016/j.cct.2021.106284
Individualization of diabetes treatment by automated insulin delivery	Biester, T; Dovc, K; Chobot, A; Tauschmann, M; Kapellen, T	Germany	Europe	Article	2021	Diabetes mellitus type I	Metabolic diseases	Review/meta-analysis	Monatsschr Kinderheilkd. 2021 Jul 13:1-8	MONATSSCHR KINDERHEILKD	NA	NA	0	Trearment	10.1007/s00112-021-01239-0
The effect of the COVID-19 pandemic on telemedicine in pediatric diabetes centers in Italy: Results from a longitudinal survey	Tornese G, Schiaffini R, Mozzillo E, Franceschi R, Frongia AP, Scaramuzza A	Italy	Europe	Article	2021	Diabetes mellitus type I	Metabolic diseases	Observational studies	Diabetes Research and Clinical Practice (2021) 179,109030	DIABETES RESEARCH AND CLINICAL PRACTICE	NA	NA	4	Follow up	10.1016/j.diabres.2021.109030
Adapting to telemedicine in the COVID-19 era: Feasibility of dried blood spot testing for hemoglobin A1c	Roberts AJ, Malik F, Pihoker C, Dickerson JA.	United Kingdom	Europe	Article	2021	Diabetes mellitus type I	Metabolic diseases	Interventional studies	Diabetes Metab Syndr. 2021 Jan-Feb15(1):433-437	DIABETOLOGY & METABOLIC SYNDROME	NA	NA	1	Follow up	10.1016/j.dsx.2021.02.010

School-based autism evaluations in the COVID-19 era	Brunson McClain M, Roanhorse TT, Harris B, Heyborne M, Zemantic PK, Azad G.	United States of America	North America	Article	2021	Autism spectrum disorders	Neurodevelopmental diseases or disorders	Review/meta-analysis	Sch Psychol. 2021 Sep36(5):377-387	SCHOOL PSYCHOLOGY	NA	NA	1	Treament	10.1037/spq0000447
Video Consultation for Parents with a Child Newly Diagnosed with Type 1 Diabetes: A Qualitative Study	Doerdelmann, J; Frielitz, FS; Lange, K; Meinsen, T; Reimers, S; Ottersberg, T; Katalinic, A; Hiort, O; von Sengbusch, S	Germany	Europe	Article	2021	Diabetes mellitus type I	Metabolic diseases	Case report/ case series	Exp Clin Endocrinol Diabetes. 2021 Oct 20	EXPERIMENTAL AND CLINICAL ENDOCRINOLOGY AND DIABETES	NA	NA	0	Follow up	10.1055/a-1655-5471
Use of Telehealth in Fellowship-Affiliated Developmental Behavioral Pediatric Practices During the COVID-19 Pandemic	Wallis KE, Mulé C, Mittal S, Cerda N, Shaffer R, Scott A, Langkamp D, Augustyn M, Perrin E, Soares N, Blum NJ.	United States of America	North America	Article	2021	Conduct disorder	Neurodevelopmental diseases or disorders	Observational studies	J Dev Behav Pediatr. 2021 May 142(4):314-321	JOURNAL OF DEVELOPMENTAL AND BEHAVIORAL PEDIATRICS	NA	NA	8	Follow up	10.1097/DBP.0000000000000897
Using telehealth for rural paediatric diabetics: Does it deliver good care?	Williams M.	United States of America	North America	Article	2021	Diabetes mellitus type I	Metabolic diseases	Observational studies	J Paediatr Child Health. 2021 Jan57(1):109-113	JOURNAL OF PAEDIATRICS AND CHILD HEALTH	NA	NA	0	Follow up	10.1111/jpc.15149
Effectiveness of Internet and Phone-Based Interventions on Diabetes Management of Children and Adolescents With Type 1 Diabetes: A Systematic Review	Zhao, XL; Huang, HQ; Zheng, SL	China	Asia	Article	2021	Diabetes mellitus type I	Metabolic diseases	Review/meta-analysis	Worldviews Evid Based Nurs. 2021 Jun;18(3):217-225	WORLDVIEWS ON EVIDENCE-BASED NURSING	NA	NA	1	Treament	10.1111/wvn.12511
Disparities in Telemedicine Use for Subspecialty Diabetes Care During COVID-19 Shelter-In-Place Orders	Haynes SC, Kompala T, Neinstein A, Rosenthal J, Crossen S.	United States of America	North America	Article	2021	Diabetes mellitus type I	Metabolic diseases	Interventional studies	J Diabetes Sci Technol. 2021 Sep15(5):986-992	JOURNAL OF DIABETES SCIENCE AND TECHNOLOGY	NA	NA	5	Diagnosis & Treatment/Follow-up	10.1177/19322968219978 51
How Providers in Child Neurology Transitioned to Telehealth During COVID-19 Pandemic	Bain J.M., Dyer C.-A., Galvin M., Goldman S., Selman J., Silver W.G., Tom S.E.	United States of America	North America	Article	2021	Autism spectrum disorders	Neurodevelopmental diseases or disorders	Observational studies	Child Neurology Open (2021) 8	CHILD NEUROLOGY OPEN	NA	NA	0	Follow up	10.1177/2329048X211022976
Mental Health Implications of the COVID-19 Pandemic Among Children and Adolescents: What Do We Know so Far?	Listernick, ZI; Badawy, SM	United States of America	North America	Article	2021	Major depressive disorder	Mental disorders	Review/meta-analysis	Pediatric Health Med Ther. 2021 Dec 20;12:543-549	PEDIATRIC HEALTH, MEDICINE AND THERAPEUTICS	NA	NA	0	Diagnosis & Treatment/Follow-up	10.2147/PHMT.S315887
Online health survey on epileptic children during coronavirus disease-2019 pandemic	Azmy A., Abushady E.M., Shady M.M.A.	Egypt	Africa	Article	2021	Epilepsy	Brain disorders	Observational studies	Open Access Macedonian Journal of Medical Sciences (2021) 9:B (392-397)	OPEN ACCESS MACEDONIAN JOURNAL OF MEDICAL SCIENCES	NA	NA	0	Follow up	10.3889/oamjms.2021.6128

The Effect of Web-based Diabetes Education on the Metabolic Control, Self-efficacy and Quality of Life of Adolescents with Type 1 Diabetes Mellitus in Turkey	Ayar D.,Ozturk C.,Grey M.	Turkey	Europe	Article	2021	Diabetes mellitus type I	Metabolic diseases	Interventional studies	Journal of Pediatric Research 2021 8:2 (131-138)	JOURNAL OF PEDIATRIC RESEARCH	NA	NA	0	Follow up	10.4274/JPR.GALENOS.2020.61214
Telehealth and Autism Prior to and in the Age of COVID-19: A Systematic and Critical Review of the Last Decade	Ellison KS, Guidry J, Picou P, Adenuga P, Davis TE 3rd.	United States of America	North America	Article	2021	Autism spectrum disorders	Neurodevelopmental diseases or disorders	Review/meta-analysis	Clin Child Fam Psychol Rev. 2021 Sep24(3):599-630	CLINICAL CHILD AND FAMILY PSYCHOLOGY REVIEW	NA	NA	6	Diagnosis & Treatment/Follow-up	10.1007/s10567-021-00358-0
Using the Hub and Spoke Model of Telemental Health to Expand the Reach of Community Based Care in the United States	Williams, C	United States of America	North America	Article	2021	Attention-deficit/hyperactivity disorder	Neurodevelopmental diseases or disorders	Interventional studies	Community Ment Health J. 2021 Jan;57(1):49-56	COMMUNITY MENTAL HEALTH JOURNAL	NA	NA	1	Diagnosis	10.1007/s10597-020-00675-8
Newest Diabetes-Related Technologies for Pediatric Type 1 Diabetes and Its Impact on Routine Care: a Narrative Synthesis of the Literature	Dos Santos, TJ; Rodrigues, TC; Punales, M; Arrais, RF; Kopacek, C	Spain	Europe	Article	2021	Diabetes mellitus type I	Metabolic diseases	Review/meta-analysis	Curr Pediatr Rep. 2021 Aug 20:1-12	CURRENT PEDIATRICS REPORTS	NA	NA	0	Trearment	10.1007/s40124-021-00248-7
Current Trends in Telehealth Applications to Deliver Social Communication Interventions for Young Children with or at Risk for Autism Spectrum Disorder	Simacek, J; Elmquist, M; Dimian, AF; Reichle, J	United States of America	North America	Article	2021	Autism spectrum disorders	Neurodevelopmental diseases or disorders	Review/meta-analysis	Curr Dev Disord Rep. 2021;8(1):15-23	CURRENT DEVELOPMENTAL DISORDERS REPORTS	NA	NA	9	Trearment	10.1007/s40474-020-00214-w
People living with type 1 diabetes point of view in COVID-19 times (COVIDT1 study): Disease impact, health system pitfalls and lessons for the future	Tejera-Perez C, Moreno-Pérez Ó, Rios J, Reyes-García R.	Spain	Europe	Article	2021	Diabetes mellitus type I	Metabolic diseases	Observational studies	Diabetes Res Clin Pract. 2021 Jan171:108547	DIABETES RESEARCH AND CLINICAL PRACTICE	NA	NA	6	Trearment	10.1016/j.diabres.2020.108547
The impact of a prolonged lockdown and use of telemedicine on glycemic control in people with type 1 diabetes during the COVID-19 outbreak in Saudi Arabia	Alharthi SK, Alyusuf EY, Alguwaihes AM, Alfadda A, Al-Sofiani ME.	Saudi Arabia	Asia	Article	2021	Diabetes mellitus type I	Metabolic diseases	Observational studies	Diabetes Res Clin Pract. 2021 Mar173:108682	DIABETES RESEARCH AND CLINICAL PRACTICE	NA	NA	8	Follow up	10.1016/j.diabres.2021.108682
Virtual training on the hybrid close loop system in people with type 1 diabetes (T1D) during the COVID-19 pandemic	Gómez AM, Henao D, Parra D, Kerguelen A, Pinilla MV, Muñoz OM, Rondón M.	Colombia	South America	Article	2021	Diabetes mellitus type I	Metabolic diseases	Observational studies	Diabetes Metab Syndr. 2021 Jan-Feb15(1):243-247	DIABETOLOGY & METABOLIC SYNDROME	NA	NA	11	Trearment	10.1016/j.dsx.2020.12.041

Impact of Technology-Based Interventions on Patient-Reported Outcomes in Asthma: A Systematic Review	Doshi H, Hsia B, Shahani J, Mowrey W, Jariwala SP.	United States of America	North America	Article	2021	Asthma	Asthma	Review/meta-analysis	J Allergy Clin Immunol Pract. 2021 Jun;9(6):2336-2341	JOURNAL OF ALLERGY AND CLINICAL IMMUNOLOGY	NA	NA		7	Trearment	10.1016/j.jaip.2021.01.027
When people with type 1 diabetes become adults - Diabetes technology and transition - do we need new models?	Datz N, Kordonouri O, Danne T. Wenn	Germany	Europe	Article	2021	Diabetes mellitus type I	Metabolic diseases	Review/meta-analysis	Dtsch Med Wochenschr. 2021 Sep;146(18):1200-1205. German	THE DEUTSCHE MEDIZINISCHE WOCHENSCHRIFT	NA	NA		0	Trearment	10.1055/a-1332-4603
Efficacy of a Telehealth Parent Training Intervention for Children with Autism Spectrum Disorder: Rural versus Urban Areas	Dahiya A.V., Ruble L., Kuravackel G., Scarpa A.	United States of America	North America	Article	2021	Autism spectrum disorders	Neurodevelopmental diseases or disorders	Interventional studies	Evidence-Based Practice in Child and Adolescent Mental Health (2021), 7(1), pp. 41-55	EVIDENCE-BASED PRACTICE IN CHILD AND ADOLESCENT MENTAL HEALTH	NA	NA		1	Trearment	10.1080/23794925.2021.1941431
Telehealth Delivery of the RELAX Intervention for Families of Adolescents Diagnosed with ADHD: Preliminary Treatment Outcomes and Evidence of Acceptability and Feasibility	Breaux R., Shroff D.M., Cash A.R., Swanson C.S., Carlton C., Bertollo J.R., Dahiya A.V.	United States of America	North America	Article	2021	Attention-deficit/hyperactivity disorder	Neurodevelopmental diseases or disorders	Interventional studies	Evidence-Based Practice in Child and Adolescent Mental Health (2021)	EVIDENCE-BASED PRACTICE IN CHILD AND ADOLESCENT MENTAL HEALTH	NA	NA		0	Trearment	10.1080/23794925.2021.1970053
The Value of Telehealth and a Team-Based Approach in Improving Developmental and Behavioral Care During the COVID-19 Pandemic	Barnhardt EW, Steingass K, Levine A, Jurbank M, Piercefield J, Nyp SS.	United States of America	North America	Article	2021	Conduct disorder	Neurodevelopmental diseases or disorders	Case report/ case series	J Dev Behav Pediatr. 2021 Sep 142(7):602-604	JOURNAL OF DEVELOPMENTAL AND BEHAVIORAL PEDIATRICS	NA	NA		1	Trearment	10.1097/DBP.0000000000000997
Telehealth in type 1 diabetes	Kompala, T; Neinstein, AB	United States of America	North America	Article	2021	Diabetes mellitus type I	Metabolic diseases	Review/meta-analysis	Current Opinion in Endocrinology & Diabetes and Obesity: February 2021 - Volume 28 - Issue 1 - p 21-29	CURRENT OPINION IN ENDOCRINOLOGY & DIABETES AND OBESITY	NA	NA		10	Trearment	10.1097/MED.0000000000000600
The results of mobile tecnologies using for achieving and maintenance of asthma control in children	Kalugina V., Namazova-Baranova L., Levina J., Vishneva E., Arimova P., Alekseeva A., Efendieva K., Volkov K.	Russia	Europe	Conference abstract	2021	Asthma	Asthma	Interventional studies	Allergy: European Journal of Allergy and Clinical Immunology (2021) 76:SUPPL 110 (113)	ALLERGY: EUROPEAN JOURNAL OF ALLERGY AND CLINICAL IMMUNOLOGY	NA	NA		0	Follow up	10.1111/all.15095
Telemedicine in epilepsy management during the coronavirus disease 2019 pandemic	Kikuchi K., Hamano S.-I., Horiguchi A., Nonoyama H., Hirata Y., Matsuura R., Koichihara R., Oka A., Hirano D.	Japan	Asia	Article	2021	Epilepsy	Brain disorders	Observational studies	Pediatrics international : official journal of the Japan Pediatric Society (2021)	PEDIATRICS INTERNATIONAL : OFFICIAL JOURNAL OF THE JAPAN PEDIATRIC SOCIETY	NA	NA		3	Follow up	10.1111/ped.14972

Mobile technologies in achieving and maintaining asthma control in children: First results of MedQuizBot Chat Bot	Arimova P.S., Namazova-Baranova L.S., Levina J.G., Kalugina V.G., Vishneva E.A., Kharitonova E.Yu.	Russia	Europe	Article	2021	Asthma	Asthma	Interventional studies	Pediatricheskaya Farmakologiya (2021) 18:3 (214-220)	PEDIATRICHESKAYA FARMAKOLOGIYA	NA	NA	0	Follow up	10.15690/pf.v18i3.2279
Development and validation of a MHEALTH technology for the promotion of self-care for adolescents with diabetes	Alves LFPA, Maia MM, Araújo MFM, Damasceno MMC, Freitas RWJF.	Brazil	South America	Article	2021	Diabetes mellitus type I	Metabolic diseases	Study protocol/Pilot study	Cien Saude Colet. 2021 May26(5):1691-1700	CIÊNCIA & SAÚDE COLETIVA	NA	NA	0	Trearment	10.1590/1413-81232021265.04602021
Diagnosis of Autistic Spectrum Disorder-ASD, adapting to the new reality, Telehealth	Velarde-Incháustegui M., Ignacio-Espíritu M.E., Cárdenas-Soza A.	Peru	South America	Article	2021	Autism spectrum disorders	Neurodevelopmental diseases or disorders	Interventional studies	Revista de Neuro-Psiquiatria (2021) 84:3 (175-182)	REVISTA DE NEURO-PSIQUIATRIA	NA	NA	0	Diagnosis	10.20453/rnp.v84i3.4034
Improvement in glycaemic control in paediatric and young adult type 1 diabetes patients during COVID-19 pandemic: role of telemedicine and lifestyle changes	Lazzeroni P, Motta M, Monaco S, Laudisio SR, Furoncoli D, Maffini V, Rubini M, Tchana B, Ruberto C, Dodi I, Iovane B.	Italy	Europe	Article	2021	Diabetes mellitus type I	Metabolic diseases	Review/meta-analysis	Acta Biomed. 2021 Nov 392(5):e2021399	ACTA BIOMEDICA	NA	NA	1	Follow up	10.23750/abm.v92i5.11911
Nocturnal Hypoglycaemia in Patients with Diabetes Mellitus: Database Analysis of a Cohort Using Telemedicine Support for Self-Monitoring of Blood Glucose over a 10-Year-Long Period	Jermendy G, Kecskes A, Nagy A.	Hungary	Europe	Article	2021	Diabetes mellitus type I	Metabolic diseases	Observational studies	Medicina (Kaunas). 2021 Feb 1457(2):167	MEDICINA (KAUNAS)	NA	NA	0	Follow up	10.3390/medicina57020167
Use of telemedicine for the management of type 1 diabetes in children and adolescents in Bangladesh during the COVID-19 pandemic	Zabeen, B; Bhowmik, B; Huda, K; Naz, F; Tayyeb, S; Azad, K	Bangladesh	Asia	Article	2021	Diabetes mellitus type I	Metabolic diseases	Observational studies	Journal of Diabetology, 2021; 12(18-21)	JOURNAL OF DIABETOLOGY	NA	NA	2	Follow up	10.4103/jod.jod_55_20
Technology-Based Assessments and Treatments of Anxiety in Autistic Individuals: Systematic Review and Narrative Synthesis	Adams, L; Valmaggia, L; Simonoff, E	United Kingdom	Europe	Article	2021	Autism spectrum disorders	Neurodevelopmental diseases or disorders	Review/meta-analysis	NA	REVIEW JOURNAL OF AUTISM AND DEVELOPMENTAL DISORDERS	NA	NA	0	Trearment	https://doi.org/10.1007/s40489-021-00275-6
Selection of trustworthy crowd workers for telemedical diagnosis of pediatric autism spectrum disorder	Washington P, Leblanc E, Dunlap K, Penev Y, Varma M, Jung JY, Chrisman B, Sun MW, Stockham N, Paskov KM, Kalantarian H, Voss C, Haber N, Wall DP.	United States of America	North America	Article	2021	Autism spectrum disorders	Neurodevelopmental diseases or disorders	Interventional studies	Pac Symp Biocomput. 202126:14-25	PACIFIC SYMPOSIUM ON BIOCOMPUTING	NA	NA	7	Diagnosis	NA

COVID-19 lockdown and episodes of hypoglycemia among patients with diabetes fasting the holy month of ramadan 2020	ALGhareeb Z.A., AlSaffar Z., Alnaji A., Al Ghareeb G., Al Maalu G.	Saudi Arabia	Asia	Conference abstract	2021	Diabetes mellitus type I	Metabolic diseases	Observational studies	Revista Argentina de Endocrinologia y Metabolismo (2021) 58:SUPPL 1 (123-125)	REVISTA ARGENTINA DE ENDOCRINOLOGIA Y METABOLISMO	NA	NA	0	Follow up	NA
Physician and patient satisfaction with the switch to remote outpatient encounters in epilepsy clinics during the Covid-19 pandemic	Teng, T; Sareidaki, DE; Chemaly, N; Bar, C; Coste-Zeitoun, D; Kuchenbuch, M; Nabbout, R	France	Europe	Article	2021	Epilepsy	Brain disorders	Observational studies	Seizure, Volume 91, 2021, Pages 60-65	SEIZURE-EUROPEAN JOURNAL OF EPILEPSY	3,184	NEUROSCIENCES	1	Diagnosis & Treatment/Follow-up	10.1016/j.seizure.2021.05.013
Utilization of telemedicine to support caregivers of young children with ASD and their Part C service providers: a comparison of intervention outcomes across three models of service delivery	Corona LL, Stainbrook JA, Simcoe K, Wagner L, Fowler B, Weitlauf AS, Juárez AP, Warren Z.	United States of America	North America	Article	2021	Conduct disorder	Neurodevelopmental diseases or disorders	Observational studies	J Neurodev Disord. 2021 Sep 1513(1):38	JOURNAL OF NEURODEVELOPMENTAL DISORDERS	4,025	NEUROSCIENCES	0	Trearment	10.1186/s11689-021-09387-w
DINOSAUR: an integrated cognitive-behavioral treatment for anxiety in young children with ASD	Keefer A., Vasa R.A.	United States of America	North America	Article	2021	Anxiety disorders	Mental disorders	Interventional studies	Journal of Neurodevelopmental Disorders (2021) 13:1	JOURNAL OF NEURODEVELOPMENTAL DISORDERS	4,025	NEUROSCIENCES	0	Trearment	10.1186/s11689-021-09396-9
A multidisciplinary telerehabilitation approach for supporting social interaction in autism spectrum disorder families: An italian digital platform in response to covid-19	Vallefuoco E., Purpura G., Gison G., Bonifacio A., Tagliabue L., Broggi F., Scuccimarra G., Pepino A., Nacinovich R.	Italy	Europe	Article	2021	Autism spectrum disorders	Neurodevelopmental diseases or disorders	Interventional studies	Brain Sciences (2021) 11:11	BRAIN SCIENCES	3,394	NEUROSCIENCES	0	Trearment	10.3390/brainsci11111404
Effects of mobile health interventions on improving glycemic stability and quality of life in patients with type 1 diabetes: A meta-analysis	Chin-Jung L, Hsiao-Yean C, Yeu-Hui C, Kuan-Chia L, Hui-Chuan H.	Taiwan	Asia	Article	2021	Diabetes mellitus type I	Metabolic diseases	Review/meta-analysis	Res Nurs Health. 2021 Feb44(1):187-200	RESEARCH IN NURSING & HEALTH	2,228	NURSING	1	Diagnosis & Treatment/Follow-up	10.1002/nur.22094
A Telehealth Initiative to Decrease No-Show Rates in a Pediatric Asthma Mobile Clinic	Van Houten L, Deegan K, Siemer M, Walsh S.	United States of America	North America	Article	2021	Asthma	Asthma	Interventional studies	J Pediatr Nurs. 2021 Jul-Aug59:143-150	JOURNAL OF PEDIATRIC NURSING-NURSING CARE OF CHILDREN & FAMILIES	2,145	NURSING	0	Prevention	10.1016/j.pedn.2021.04.005
The Use of Technology to Improve Outcomes in Children with Asthma	McGee PL.	United States of America	North America	Article	2021	Asthma	Asthma	Review/meta-analysis	J Pediatr Nurs. 2021 Nov-Dec61:173-175	JOURNAL OF PEDIATRIC NURSING-NURSING CARE OF CHILDREN & FAMILIES	2,145	NURSING	0	Follow up	10.1016/j.pedn.2021.05.014

A Quality Improvement Initiative to Improve Attention-Deficit/Hyperactivity Disorder Follow-Up Rates Using School-Based Telemedicine	Milne Wenderlich A, Li R, Baldwin CD, Contento N, Herendeen N, Rand CM. A	United States of America	North America	Article	2021	Attention-deficit/hyperactivity disorder	Neurodevelopmental diseases or disorders	Observational studies	Acad Pediatr. 2021 Sep-Oct;21(7):1253-1261	ACADEMIC PEDIATRICS	3,107	PEDIATRICS	0	Follow up	10.1016/j.acap.2021.04.004
Management and specialist care for users with autism spectrum disorder in day-care centers during the Covid-19 health emergency	Patane S., Scaltrito M.V., Caporlingua C., Tricoli M., Finocchio S., Alosi N., Maggio R., Gervasi T., Motta G.	Italy	Europe	Conference abstract	2021	Autism spectrum disorders	Neurodevelopmental diseases or disorders	Observational studies	Developmental Medicine and Child Neurology (2021) 63:SUPPL 2 (49)	DEVELOPMENTAL MEDICINE AND CHILD NEUROLOGY	5,449	PEDIATRICS	0	Follow up	10.1111/dmcn.14881
Changes to care delivery at nine international pediatric diabetes clinics in response to the COVID-19 global pandemic	Sarteau AC, Souris KJ, Wang J, Ramadan AA, Addala A, Bowlby D, Corathers S, Forsander G, King B, Law JR, Liu W, Malik F, Pihoker C, Seid M, Smart C, Sundberg F, Tandon N, Yao M, Headley T, Mayer-Davis E.	multicenter	More than one continent	Article	2021	Diabetes mellitus type I	Metabolic diseases	Observational studies	Pediatr Diabetes. 2021 May22(3):463-468	PEDIATRIC DIABETES	4,866	PEDIATRICS	6	Diagnosis & Treatment/Follow-up	10.1111/pedi.13180
"Telemedicine" in children and adolescents with type 1 diabetes: A systematic review and meta-analysis of randomized controlled trials	Chettiyarammel M.	China	Asia	Article	2021	Diabetes mellitus type I	Metabolic diseases	Review/meta-analysis	Pediatric Diabetes (2021) 22:SUPPL 29 (5)	PEDIATRIC DIABETES	4,866	PEDIATRICS	1	Diagnosis & Treatment/Follow-up	10.1111/pedi.13197
Influence of COVID 19 on patients/parents fears, schooling after lock down and use of telemedicine in a German pediatric diabetes center	Kapellen T., Bartelt H., Klamt S., Mauer A., Kiess W.	Germany	Europe	Conference abstract	2021	Diabetes mellitus type I	Metabolic diseases	Observational studies	Pediatric Diabetes (2021) 22:SUPPL 29 (86)	PEDIATRIC DIABETES	4,866	PEDIATRICS	0	Follow up	10.1111/pedi.13198
Exploring the role of peer support group in patients of type 1 diabetes during the times of COVID-19	Ahmad A., Siddiqui M.A.	India	Asia	Conference abstract	2021	Diabetes mellitus type I	Metabolic diseases	Observational studies	Pediatric Diabetes (2021) 22:SUPPL 29 (90)	PEDIATRIC DIABETES	4,866	PEDIATRICS	0	Follow up	10.1111/pedi.13198
Anxiety, depression and glycemic control during COVID-19 pandemic: A cross sectional study in youths with type 1 diabetes	Martino M., Cusinato M., Gabrielli C., Tassara L., Righetto E., Sartori A., Debertolis G., Galderisi A., Moretti C.	Italy	Europe	Conference abstract	2021	Diabetes mellitus type I	Metabolic diseases	Observational studies	Pediatric Diabetes (2021) 22:SUPPL 29 (85)	PEDIATRIC DIABETES	4,866	PEDIATRICS	0	Trearment	10.1111/pedi.13198
A randomized control trial: To determine the efficacy of SMS as a tool for health education in families of children with type 1 diabetes in Pakistan	Rahman A., Khan M.S., Malik Q.U., Ikram F., Tabussam S., Nisa N.U.	Pakistan	Asia	Conference abstract	2021	Diabetes mellitus type I	Metabolic diseases	Interventional studies	Pediatric Diabetes 2021 22:SUPPL 30 (9-10)	PEDIATRIC DIABETES	4,866	PEDIATRICS	0	Trearment	10.1111/pedi.13268

Managing pediatric diabetes in the times of pandemic in India	Hasnani D., Chavda V., Meheriya V., Hariharan N., Hasnani S.	India	Asia	Conference abstract	2021	Diabetes mellitus type I	Metabolic diseases	Observational studies	Pediatric Diabetes (2021) 22:SUPPL 30 (104)	PEDIATRIC DIABETES	4,866	PEDIATRICS	0	Follow up	10.1111/pedi. 13269
Does telemedicine work well to support adolescents with type 1 diabetes?: A qualitative study with parents and diabetes care team members	Malik F., Senturia K., Moreno M., Mangione-Smith R., Pihoker C., Pollock A., Christakis D.	United States of America	North America	Conference abstract	2021	Diabetes mellitus type I	Metabolic diseases	Observational studies	Pediatric Diabetes (2021) 22:SUPPL 30 (75-77)	PEDIATRIC DIABETES	4,866	PEDIATRICS	0	Follow up	10.1111/pedi. 13269
Use of telemedicine in the care of pediatric patients with type 1 diabetes during COVID-19 pandemic in a Public University Hospital in Brazil	Cudizio L., Noronha R., Calliari L.E., Arruda B.F., Cunha N.D.S., Soares N.L.R., Cazerta N.A.C., Lyra A., Mourão A.S.F., Ferreira F.R., Kinoshita R.I.S., Sampaio A.G., Lopes A.E.M., Amorim R.P., Camargo A.C.C.	Brazil	South America	Conference abstract	2021	Diabetes mellitus type I	Metabolic diseases	Observational studies	Pediatric Diabetes (2021) 22:SUPPL 30 (81)	PEDIATRIC DIABETES	4,866	PEDIATRICS	0	Follow up	10.1111/pedi. 13269
Effect of telemedicine on the management of type 1 diabetes during COVID-19 pandemic	Saboo B., Saiyed M., Pancholi M.	India	Asia	Conference abstract	2021	Diabetes mellitus type I	Metabolic diseases	Interventional studies	Pediatric Diabetes (2021) 22:SUPPL 30 (53-54)	PEDIATRIC DIABETES	4,866	PEDIATRICS	0	Trearment	10.1111/pedi. 13269
Does a telemedicine approach improve glycaemic control and quality of life in children and adolescents with type 1 diabetes?	Eilidh C., Franklin V.	United Kingdom	Europe	Conference abstract	2021	Diabetes mellitus type I	Metabolic diseases	Review/meta-analysis	Pediatric Diabetes (2021) 22:SUPPL 30 (79)	PEDIATRIC DIABETES	4,866	PEDIATRICS	0	Trearment	10.1111/pedi. 13269
The effect of 'telemedicine' on glycaemic control and other clinical outcomes in children and adolescents with type 1 diabetes-a systematic review and metaanalysis of randomised controlled trials	Parakkal M.C.T., Sukumar N.	Ireland	Europe	Conference abstract	2021	Diabetes mellitus type I	Metabolic diseases	Review/meta-analysis	Archives of Disease in Childhood (2021) 106:SUPPL 2 (A89)	ARCHIVES OF DISEASE IN CHILDHOOD	3,801	PEDIATRICS	0	Follow up	10.1136/archdischild-2021-europaediatrics.210
COVID-19, lockdown 1.0, and the move to telemedicine: Impact on glycated haemoglobin in paediatric diabetes mellitus	Armon-Drewett E., Aswani N., Smith J., Tinklin T.	United Kingdom	Europe	Conference abstract	2021	Diabetes mellitus type I	Metabolic diseases	Observational studies	Archives of Disease in Childhood (2021) 106:SUPPL 1 (A299-A300)	ARCHIVES OF DISEASE IN CHILDHOOD	3,801	PEDIATRICS	0	Trearment	10.1136/archdischild-2021-rcpch.522
Caregiver Satisfaction and Effectiveness of Teleconsultation in Children and Adolescents With Migraine During the Ongoing COVID-19 Pandemic	Sharawat IK, Panda PK.	India	Asia	Article	2021	Migraine	Brain disorders	Interventional studies	J Child Neurol. 2021 Mar36(4):296-303	JOURNAL OF CHILD NEUROLOGY	1,987	PEDIATRICS	11	Diagnosis & Treatment/Follow-up	10.1177/0883073820968653

Availability of Services and Caregiver Burden: Supporting Individuals With Neurogenetic Conditions During the COVID-19 Pandemic	Kowanda M, Cartner L, Kentros C, Geltzeiler AR, Singer KE, Weaver WC, Lehman CD, Smith S, Smith RS, Walsh LK, Diehl K, Nagpal N, Brooks E, Mebane CM, Wilson AL, Marvin AR, White LC, Law JK, Jensen W, Daniels AM, Tjernagel J, Snyder LG, Taylor CM, Chung WK.	United States of America	North America	Article	2021	Autism spectrum disorders	Neurodevelopmental diseases or disorders	Interventional studies	J Child Neurol. 2021 Aug36(9):760-767	JOURNAL OF CHILD NEUROLOGY	1,987	PEDIATRICS	3	Trearment	10.1177/08830738211001209
An accurate alternative method for diagnoses of autismspectrum disorder using video telehealth parent led play in themilitary medical system	Butsch M., Jardon-Aites M., Cartisano T., Jordan B., Flake E., Roy D., Tolson D.	United States of America	North America	Conference abstract	2021	Autism spectrum disorders	Neurodevelopmental diseases or disorders	Observational studies	Pediatrics (2021) 147:3 (1029-1030)	PEDIATRICS	7,125	PEDIATRICS	0	Diagnosis	10.1542/peds.147.3_MeetingAbstract.1029
The developmental-behavioral extender-a force multiplierquality improvement initiative	Dunn C., Flake E., Pragani J., Cartisano T., Chan S.	United States of America	North America	Conference abstract	2021	Autism spectrum disorders	Neurodevelopmental diseases or disorders	Interventional studies	Pediatrics (2021) 147:3 (1057-1058)	PEDIATRICS	7,125	PEDIATRICS	0	Follow up	10.1542/peds.147.3_MeetingAbstract.1057
Implementation study: Challenges of rapid telemedicineimplementation into a small community pediatric allergy-asthma practice	Phillips J., Saini S.K.	United States of America	North America	Conference abstract	2021	Asthma	Asthma	Observational studies	Pediatrics (2021) 147:3 (969-970)	PEDIATRICS	7,125	PEDIATRICS	0	Follow up	10.1542/peds.147.3_MeetingAbstract.969
Novel home direct-to-consumer telehealth solutions forchildren with mental health disorders	Norman S., Atabaki S., Atmore K., Biddle C.L., DiFazio M., Felton D., Fox E., Marschall D., Newman J., Robb A.S., Rowland C., Selekman R.E., Slovin A., Stein M., Strang J., Sable C.	United States of America	North America	Conference abstract	2021	Attention-deficit/hyperactivity disorder	Neurodevelopmental diseases or disorders	Interventional studies	Pediatrics (2021) 147:3 (971-972)	PEDIATRICS	7,125	PEDIATRICS	0	Diagnosis	10.1542/peds.147.3_MeetingAbstract.971-a
Retrospective review of pediatric asthma follow-up viatelemedicine and in-person visits	Turner J.H., Perry T.T., Steele R.W., Famuyide L.I., Margiotta C.A., Spray B.J.	United States of America	North America	Conference abstract	2021	Asthma	Asthma	Review/meta-analysis	Pediatrics (2021) 147:3 (982)	PEDIATRICS	2,66	PEDIATRICS	0	Follow up	10.1542/peds.147.3_MeetingAbstract.982
Telemedicine for Asthma Follow-up in Children During COVID-19 Pandemic	Jain S, Thakur C, Kumar P, Goyal JP, Singh K.	India	Asia	Article	2021	Asthma	Asthma	Review/meta-analysis	Indian J Pediatr. 2021 Oct88(10):1050	INDIAN JOURNAL OF PEDIATRICS	1,967	PEDIATRICS	3	Follow up	10.1007/s12098-021-03868-5
Facilitators and Barriers to Implementation of School-Based Telehealth Asthma Care: Program Champion Perspectives	Johnson EE, MacGeorge C, King KL, Andrews AL, Teufel RJ 2nd, Kruis R, Hale KC, Ford DW, Sterba KR.	United States of America	North America	Article	2021	Asthma	Asthma	Observational studies	Acad Pediatr. 2021 Sep-Oct21(7):1262-1272	ACADEMIC PEDIATRICS	3,107	PEDIATRICS	2	Follow up	10.1016/j.acap.2021.04.025

Addressing Pediatric Mental Health Using Telehealth During Coronavirus Disease-2019 and Beyond: A Narrative Review	Cunningham N.R., Ely S.L., Barber Garcia B.N., Bowden J.	United States of America	North America	Article	2021	Autism spectrum disorders	Neurodevelopmental diseases or disorders	Review/meta-analysis	Acad Pediatr. 2021 Sep-Oct21(7):1108-1117	ACADEMIC PEDIATRICS	3,107	PEDIATRICS	4	Trearment	10.1016/j.acap.2021.06.002
Care of Pediatric Patients with Diabetes During the Coronavirus Disease 2019 (COVID-19) Pandemic	Buggs-Saxton C.	United States of America	North America	Article	2021	Diabetes mellitus type I	Metabolic diseases	Review/meta-analysis	Pediatr Clin North Am. 2021 Oct68(5):1093-1101	PEDIATRIC CLINICS OF NORTH AMERICA	3,278	PEDIATRICS	1	Diagnosis & Treatment/Follow-up	10.1016/j.pcl.2021.05.014
Exclusive telemedicine during COVID-19 Pandemic: Impact on metabolic control, diabetes care and satisfaction of families in a Chilean little urban-rural diabetes center	Pelicand J., Silva T., Alcaino H., Silva M.	Chile	South America	Conference abstract	2021	Diabetes mellitus type I	Metabolic diseases	Interventional studies	Pediatric Diabetes (2021) 22:SUPPL 29 (22). Date of Publication: 1 Apr 2021	PEDIATRIC DIABETES	4,886	PEDIATRICS	0	Trearment	10.1111/pedi.13197
Population-level management of type 1 diabetes via continuous glucose monitoring and algorithm-enabled patient prioritization: Precision health meets population health	Ferstad, JO; Vallon, JJ; Jun, D; Gu, A; Vitko, A; Morales, DP; Leverenz, J; Lee, MY; Leverenz, B; Vasilakis, C; Osmanlliu, E; Prahalad, P; Maahs, DM; Johari, R; Scheinker, D	United States of America	North America	Article	2021	Diabetes mellitus type I	Metabolic diseases	Interventional studies	Pediatric diabetes vol. 22,7 (2021): 982-991	PEDIATRIC DIABETES	4,866	PEDIATRICS	1	Follow up	10.1111/pedi.13256
Telemedicine program in a population of children and adolescent with type 1 diabetes	Shaker K., Tinti D., Trada M., Giorda S., De Sanctis L.	Italy	Europe	Conference abstract	2021	Diabetes mellitus type I	Metabolic diseases	Interventional studies	Pediatric Diabetes (2021) 22:SUPPL 30 (81)	PEDIATRIC DIABETES	4,866	PEDIATRICS	0	Trearment	10.1111/pedi.13269
Implementing a teleHealth autism diagnostic service in Barnet in response to Covid-19 restrictions	Nasir R., Czerniewska P., Pearlman S., Nagendran G., Jenkins C., Gurney R., Bills S.	United Kingdom	Europe	Conference abstract	2021	Autism spectrum disorders	Neurodevelopmental diseases or disorders	Review/meta-analysis	Archives of Disease in Childhood (2021) 106:SUPPL 1 (A278)	ARCHIVES OF DISEASE IN CHILDHOOD	3,801	PEDIATRICS	0	Diagnosis	10.1136/archdischild-2021-rcpch.483
Proposed Assessment of Cough and Dyspnea in Children via Telemedicine in Coronavirus Disease 2019 Era: A Web Application-HOPS	Perivolaropoulos, C; Vlacha, V; Feketea, GM	Greece	Europe	Article	2021	Asthma	Asthma	Interventional studies	Clinical pediatrics vol. 60,14 (2021): 564-568.	CLINICAL PEDIATRICS	1,168	PEDIATRICS	0	Diagnosis & Treatment/Follow-up	10.1177/000992282110549 27
The MEDEA childhood asthma study design for mitigation of desert dust health effects: implementation of novel methods for assessment of air pollution exposure and lessons learned	Kouis P, Papatheodorou SI, Kakkoura MG, Middleton N, Galanakis E, Michaelidi E, Achilleos S, Mihalopoulos N, Neophytou M, Stamatelatos G, Kaniklides C, Revvas E, Tymvios F, Savvides C,	Cyprus	Europe	Article	2021	Asthma	Asthma	Study protocol/Pilot study	BMC Pediatr. 2021 Jan 621(1):13	BMC PEDIATRICS	2,125	PEDIATRICS	3	Trearment	10.1186/s12887-020-02472-4

	Koutrakis P, Yiallouros PK.														
Chronic respiratory diseases other than asthma in children: the COVID-19 tsunami	Di Cicco M, Tozzi MG, Ragazzo V, Peroni D, Kantar A.	Italy	Europe	Article	2021	Asthma	Asthma	Review/meta-analysis	Ital J Pediatr. 2021 Nov 647(1):220	ITALIAN JOURNAL OF PEDIATRICS	2,638	PEDIATRICS	0	Follow up	10.1186/s13052-021-01155-9
Getting a head start on asthma: A preschool based telehealthinitiative	Richmond D.S., Yanagisawa K.	United States of America	North America	Conference abstract	2021	Asthma	Asthma	Interventional studies	Pediatrics (2021) 147:3 (964-965)	PEDIATRICS	2,66	PEDIATRICS	0	Trearment	10.1542/peds.147.3_MeetingAbstract.964
Increasing diagnostic services for autism spectrum disorder inthe native American community: A pilot collaborative telecaremodel	Bennett A., Ray M., Zucker E., Chuo J.	United States of America	North America	Conference abstract	2021	Autism spectrum disorders	Neurodevelopmental diseases or disorders	Interventional studies	Pediatrics (2021) 147:3 (970-971)	PEDIATRICS	7,125	PEDIATRICS	0	Diagnosis	10.1542/peds.147.3_MeetingAbstract.970
Telehealth for Children With Epilepsy Is Effective and Reduces Anxiety Independent of Healthcare Setting	Klotz K.A., Borlot F., Scantlebury M.H., Payne E.T., Appendino J.P., Schönberger J., Jacobs J.	Germany	Europe	Article	2021	Epilepsy	Brain disorders	Observational studies	Frontiers in Pediatrics (2021) 9	FRONTIERS IN PEDIATRICS	3,418	PEDIATRICS	0	Follow up	10.3389/fped.2021.642381
Isn't There an App for That? The Role of Smartphone and Tablet Applications for Asthma Education and Self-Management in Adolescents	O'Connor, A; Tai, A; Carson-Chahhoud, K	Australia	Oceania	Article	2021	Asthma	Asthma	Review/meta-analysis	Children (Basel). 2021 Sep 9;8(9):786	CHILDREN-BASEL	2,863	PEDIATRICS	1	Follow up	10.3390/children8090786
The Value of Telemedicine for the Follow-up of Patients with New Onset Type 1 Diabetes Mellitus During COVID-19 Pandemic in Turkey: A Report of Eight Cases	Evin F, Er E, Ata A, Jalilova A, Demir G, Atik Altınok Y, Özen S, Darcan Ş, Gökşen D.	Turkey	Europe	Article	2021	Diabetes mellitus type I	Metabolic diseases	Case report/ case series	J Clin Res Pediatr Endocrinol. 2021 Nov 2513(4):468-472	JOURNAL OF CLINICAL RESEARCH IN PEDIATRIC ENDOCRINOLOGY	1,933	PEDIATRICS	1	Follow up	10.4274/JCRPE.GALENOS.2020.2020.0160
From Precision Metapharmacology to Patient Empowerment: Delivery of Self-Care Practices for Epilepsy, Pain, Depression and Cancer Using Digital Health Technologies	Bulaj G, Clark J, Ebrahimi M, Bald E.	United States of America	North America	Article	2021	Epilepsy	Brain disorders	Review/meta-analysis	Front Pharmacol. 2021 Apr 23;12:612602	FRONTIERS IN PHARMACOLOGY	5,811	PHARMACOLOGY & PHARMACY	1	Trearment	10.3389/fphar.2021.612602

Tele-Mental Health for Reaching Out to Patients in a Time of Pandemic: Provider Survey and Meta-analysis of Patient Satisfaction	Mazziotti R, Rutigliano G.	Italy	Europe	Article	2021	Major depressive disorder	Mental disorders	Observational studies	JMIR Ment Health. 2021 Jul 29;8(7):e26187	JMIR MENTAL HEALTH	4,388	PSYCHIATRY	2	Trearment	10.2196/26187
Effect of Layperson-Delivered, Empathy-Focused Program of Telephone Calls on Loneliness, Depression, and Anxiety Among Adults During the COVID-19 Pandemic: A Randomized Clinical Trial	Kahlon MK, Aksan N, Aubrey R, Clark N, Cowley-Morillo M, Jacobs EA, Mundhenk R, Sebastian KR, Tomlinson S.	United States of America	North America	Article	2021	Anxiety disorders	Mental disorders	Interventional studies	JAMA Psychiatry. 2021 Jun 178(6):616-622	JAMA PSYCHIATRY	21,596	PSYCHIATRY	18	Diagnosis	10.1001/jamapsychiatry.2021.0113
Low intensity treatment for clinically anxious youth: a randomised controlled comparison against face-to-face intervention	Rapee RM, Lyneham HJ, Wuthrich V, Chatterton ML, Hudson JL, Kangas M, Mihalopoulos C.	Australia	Oceania	Article	2021	Anxiety disorders	Mental disorders	Interventional studies	Eur Child Adolesc Psychiatry. 2021 Jul30(7):1071-1079	EUROPEAN CHIL AND ADOLESCENT PSYCHIATRY	4,785	PSYCHIATRY	2	Trearment	10.1007/s00787-020-01596-3
Supporting Children With Neurodevelopmental Disorders During the COVID-19 Pandemic	Summers J, Baribeau D, Mockford M, Goldhopf L, Ambrozewicz P, Szatmari P, Vorstman J.	Canada	North America	Comment/editorial/letter	2021	Autism spectrum disorders	Neurodevelopmental diseases or disorders	Observational studies	J Am Acad Child Adolesc Psychiatry. 2021 Jan60(1):2-6	JOURNAL OF THE AMERICAN ACADEMY OF CHILD & ADOLESCENT PSYCHIATRY	8,829	PSYCHIATRY	16	Follow up	10.1016/j.jaac.2020.09.011
Therapist-Guided Internet-Delivered Cognitive Behavioral Therapy vs Internet-Delivered Supportive Therapy for Children and Adolescents With Social Anxiety Disorder: A Randomized Clinical Trial	Nordh M, Wahlund T, Jolstedt M, Sahlin H, Bjureberg J, Ahlen J, Lalouni M, Salomonsson S, Vigerland S, Lavner M, Öst LG, Lenhard F, Hesser H, Mataix-Cols D, Högström J, Serlachius E.	Sweden	Europe	Article	2021	Anxiety disorders	Mental disorders	Interventional studies	JAMA Psychiatry. 2021 Jul 178(7):705-713	JAMA PSYCHIATRY	21,596	PSYCHIATRY	2	Trearment	10.1001/jamapsychiatry.2021.0469
Long-term outcomes of internet-delivered cognitive behaviour therapy for paediatric anxiety disorders: towards a stepped care model of health care delivery	Jolstedt M, Vigerland S, Mataix-Cols D, Ljótsson B, Wahlund T, Nord M, Högström J, Öst LG, Serlachius E.	Sweden	Europe	Article	2021	Conduct disorder	Neurodevelopmental diseases or disorders	Interventional studies	Eur Child Adolesc Psychiatry. 2021 Nov30(11):1723-1732	EUROPEAN CHIL AND ADOLESCENT PSYCHIATRY	4,785	PSYCHIATRY	2	Trearment	10.1007/s00787-020-01645-x
Therapist-Led, Internet-Delivered Treatment for Early Child Social Anxiety: A Waitlist-Controlled Evaluation of the iCALM Telehealth Program	Comer JS, Furr JM, Del Busto C, Silva K, Hong N, Poznanski B, Sanchez A, Cornacchio D, Herrera A, Coxe S, Miguel E, Georgiadis C, Conroy K, Puliafico A.	United States of America	North America	Article	2021	Anxiety disorders	Mental disorders	Interventional studies	Behav Ther. 2021 Sep52(5):1171-1187	BEHAVIOR THERAPY	4,183	PSYCHIATRY	8	Trearment	10.1016/j.beth.2021.01.004
TECHNOLOGY TO THE RESCUE DURING COVID-19 PANDEMIC: IMPLEMENTATION OF VIRTUAL TECHNOLOGIES OPENS	Muhle R.A., Goldstein F.P.	United States of America	North America	Conference abstract	2021	Attention-deficit/hyperactivity disorder	Neurodevelopmental diseases or disorders	Observational studies	Journal of the American Academy of Child and Adolescent Psychiatry	JOURNAL OF THE AMERICAN ACADEMY OF CHILD AND	8,829	PSYCHIATRY	0	Trearment	10.1016/j.jaac.2021.07.117

NEW AVENUES FOR CLINICAL EDUCATION, SUPPORT, AND CARE OF CLINICIANS WHO PROVIDE CARE TO PEOPLE DIAGNOSED WITH AUTISM SPECTRUM DISORDER AND INTELLECTUAL DISABILITY									(2021) 60:10 Supplement (S25)	ADOLESCENT PSYCHIATRY				
THE IMPACT OF PSYCHIATRIC COMORBIDITIES IN TRANSITION OF CARE FOR ADOLESCENTS WITH EPILEPSY	Cai, Y; Timmons-Mitchell, J; Pestana-Knight, E; Tossone, K; Cuomo, C; Zemba, D; Lachhwani, D; Miniard, A; Corder, JH; Falcone, T	United States of America	North America	Conference abstract	2021	Epilepsy	Brain disorders	Observational studies	Journal of the American Academy of Child and Adolescent Psychiatry 2021 60:10 Supplement (S185-S186)	JOURNAL OF THE AMERICAN ACADEMY OF CHILD AND ADOLESCENT PSYCHIATRY	8,829	PSYCHIATRY	0	Follow up	10.1016/j.jaac.2021.09.159
Future perspectives of robot psychiatry: can communication robots assist psychiatric evaluation in the COVID-19 pandemic era?	Yoshikawa, Y; Kumazaki, H; Kato, TA	Japan	Asia	Article	2021	Major depressive disorder	Mental disorders	Review/meta-analysis	Curr Opin Psychiatry. 2021 May 1;34(3):277-286	CURRENT OPINION IN PSYCHIATRY	4,741	PSYCHIATRY	0	Treament	10.1097/YCO.0000000000000692
Telehealth mask wearing training for children with autism during the COVID-19 pandemic	Sivaraman M, Virues-Ortega J, Roeyers H.	multicenter	More than one continent	Article	2021	Autism spectrum disorders	Neurodevelopmental diseases or disorders	Case report/ case series	J Appl Behav Anal. 2021 Jan54(1):70-86	JOURNAL OF APPLIED BEHAVIOR ANALYSIS	3,695	PSYCHOLOGY, CLINICAL	18	Treament	10.1002/jaba.802
The effects of transition to technician-delivered telehealth ABA treatment during the COVID-19 crisis: A preliminary analysis	Pollard JS, LeBlanc LA, Griffin CA, Baker JM.	United States of America	North America	Article	2021	Autism spectrum disorders	Neurodevelopmental diseases or disorders	Observational studies	J Appl Behav Anal. 2021 Jan54(1):87-102	JOURNAL OF APPLIED BEHAVIOR ANALYSIS	3,695	PSYCHOLOGY, CLINICAL	14	Treament	10.1002/jaba.803
Telehealth parent coaching to improve daily living skills for children with ASD	Gerow S, Radhakrishnan S, S Akers J, McGinnis K, Swensson R.	United States of America	North America	Article	2021	Autism spectrum disorders	Neurodevelopmental diseases or disorders	Case report/ case series	J Appl Behav Anal. 2021 Apr54(2):566-581	JOURNAL OF APPLIED BEHAVIOR ANALYSIS	3,695	PSYCHOLOGY, CLINICAL	7	Treament	10.1002/jaba.813
Iterative Development of a Daily Living Skills Intervention for Adolescents with Autism Without an Intellectual Disability	Duncan A, Liddle M, Stark LJ.	United States of America	North America	Article	2021	Autism spectrum disorders	Neurodevelopmental diseases or disorders	Review/meta-analysis	Clin Child Fam Psychol Rev. 2021 Dec24(4):744-764	CLINICAL CHILD AND FAMILY PSYCHOLOGY REVIEW	5,574	PSYCHOLOGY, CLINICAL	2	Follow up	10.1007/s10567-021-00360-6
Transitioning to Telehealth Services in a Pediatric Diabetes Clinic During COVID-19: An Interdisciplinary Quality Improvement Initiative	Brodar K.E., Hong N., Liddle M., Hernandez L., Waks J., Sanchez J., Delamater A., Davis E.	United States of America	North America	Article	2021	Diabetes mellitus type I	Metabolic diseases	Observational studies	Journal of Clinical Psychology in Medical Settings (2021)	JOURNAL OF CLINICAL PSYCHOLOGY IN MEDICAL SETTINGS	2,615	PSYCHOLOGY, CLINICAL	0	Follow up	10.1007/s10880-021-09830-z

Feasibility and acceptability of a telehealth model for autism diagnostic evaluations in children, adolescents, and adults	Matthews NL, Skepnek E, Mammen MA, James JS, Malligo A, Lyon A, Mitchell M, Kiefer SL, Smith CJ.	United States of America	North America	Article	2021	Autism spectrum disorders	Neurodevelopmental diseases or disorders	Observational studies	Autism Res. 2021 Dec14(12):2564-2579	AUTISM RESEARCH	5,216	PSYCHOLOGY, DEVELOPMENTAL	0	Diagnosis	10.1002/aur.2591
Rethinking autism spectrum disorder assessment for children during COVID-19 and beyond	Zwaigenbaum L, Bishop S, Stone WL, Ibanez L, Halladay A, Goldman S, Kelly A, Klaiman C, Lai MC, Miller M, Saulnier C, Siper P, Sohl K, Warren Z, Wetherby A.	Canada	North America	Comment/editorial/letter	2021	Autism spectrum disorders	Neurodevelopmental diseases or disorders	Review/meta-analysis	Autism Res. 2021 Nov14(11):2251-2259	AUTISM RESEARCH	5,216	PSYCHOLOGY, DEVELOPMENTAL	0	Diagnosis	10.1002/aur.2615
A Pilot Study Comparing Tele-therapy and In-Person Therapy: Perspectives from Parent-Mediated Intervention for Children with Autism Spectrum Disorders	Hao Y, Franco JH, Sundarrajan M, Chen Y.	United States of America	North America	Article	2021	Autism spectrum disorders	Neurodevelopmental diseases or disorders	Interventional studies	J Autism Dev Disord. 2021 Jan51(1):129-143	JOURNAL OF AUTISM AND DEVELOPMENTAL DISORDERS	4,291	PSYCHOLOGY, DEVELOPMENTAL	13	Trearment	10.1007/s10803-020-04439-x
Parent Perceptions of Caregiver-Mediated Telemedicine Tools for Assessing Autism Risk in Toddlers	Corona LL, Weitlauf AS, Hine J, Berman A, Miceli A, Nicholson A, Stone C, Broderick N, Francis S, Juárez AP, Vehorn A, Wagner L, Warren Z.	United States of America	North America	Article	2021	Autism spectrum disorders	Neurodevelopmental diseases or disorders	Observational studies	J Autism Dev Disord. 2021 Feb51(2):476-486	JOURNAL OF AUTISM AND DEVELOPMENTAL DISORDERS	4,291	PSYCHOLOGY, DEVELOPMENTAL	25	Diagnosis	10.1007/s10803-020-04554-9
Use of the TELE-ASD-PEDS for Autism Evaluations in Response to COVID-19: Preliminary Outcomes and Clinician Acceptability	Wagner L, Corona LL, Weitlauf AS, Marsh KL, Berman AF, Broderick NA, Francis S, Hine J, Nicholson A, Stone C, Warren Z.	United States of America	North America	Article	2021	Autism spectrum disorders	Neurodevelopmental diseases or disorders	Study protocol/Pilot study	J Autism Dev Disord. 2021 Sep51(9):3063-3072	JOURNAL OF AUTISM AND DEVELOPMENTAL DISORDERS	4,291	PSYCHOLOGY, DEVELOPMENTAL	30	Trearment	10.1007/s10803-020-04767-y
Conducting CBT for Anxiety in Children with Autism Spectrum Disorder During COVID-19 Pandemic	Kalvin CB, Jordan RP, Rowley SN, Weis A, Wood KS, Wood JJ, Ibrahim K, Sukhodolsky DG.	United States of America	North America	Comment/editorial/letter	2021	Autism spectrum disorders	Neurodevelopmental diseases or disorders	Review/meta-analysis	J Autism Dev Disord. 2021 Nov51(11):4239-4247	JOURNAL OF AUTISM AND DEVELOPMENTAL DISORDERS	4,291	PSYCHOLOGY, DEVELOPMENTAL	4	Follow up	10.1007/s10803-020-04845-1
Development and Acceptability of a New Program for Caregivers of Children with Autism Spectrum Disorder: Online Parent Training in Early Behavioral Intervention	Dai YG, Thomas RP, Brennan L, Helt MS, Barton ML, Dumont-Mathieu T, Fein DA.	United States of America	North America	Article	2021	Autism spectrum disorders	Neurodevelopmental diseases or disorders	Interventional studies	J Autism Dev Disord. 2021 Nov51(11):4166-4185	JOURNAL OF AUTISM AND DEVELOPMENTAL DISORDERS	4,291	PSYCHOLOGY, DEVELOPMENTAL	0	Trearment	10.1007/s10803-020-04863-z
Short report on research trends during the COVID-19 pandemic and use of telehealth interventions and remote brain research in children with autism spectrum disorder	Su WC, Srinivasan S, Cleffi C, Bhat A.	United States of America	North America	Article	2021	Autism spectrum disorders	Neurodevelopmental diseases or disorders	Observational studies	Autism. 2021 Aug25(6):1816-1822	AUTISM	5,689	PSYCHOLOGY, DEVELOPMENTAL	5	Trearment	10.1177/13623613211004795

Transitioning to Telemedicine During COVID-19: Impact on Perceptions and Use of Telemedicine Procedures for the Diagnosis of Autism in Toddlers	Wagner L., Weitlauf A.S., Hine J., Corona L.L., Berman A.F., Nicholson A., Allen W., Black M., Warren Z.	United States of America	North America	Article	2021	Autism spectrum disorders	Neurodevelopmental diseases or disorders	Observational studies	Journal of Autism and Developmental Disorders (2021)	JOURNAL OF AUTISM AND DEVELOPMENTAL DISORDERS	4,291	PSYCHOLOGY, DEVELOPMENTAL	2	Diagnosis	10.1007/s10803-021-05112-7
Predictors of Satisfaction with Autism Treatment Services During COVID-19	Ferguson E.F., Jimenez-Muñoz M., Feerst H., Vernon T.W.	United States of America	North America	Article	2021	Autism spectrum disorders	Neurodevelopmental diseases or disorders	Observational studies	Journal of Autism and Developmental Disorders (2021)	JOURNAL OF AUTISM AND DEVELOPMENTAL DISORDERS	4,291	PSYCHOLOGY, DEVELOPMENTAL	2	Follow up	10.1007/s10803-021-05232-0
Bridging the needs of adolescent diabetes care during coronavirus disease 2019: A nurse-led telehealth initiative	Lim S.T., Chin X., Yap F.	Singapore	Asia	Conference abstract	2021	Diabetes mellitus type I	Metabolic diseases	Observational studies	J Adolesc Health. 2020 Oct;67(4):615-617	JOURNAL OF ADOLESCENT HEALTH	4,828	PSYCHOLOGY, DEVELOPMENTAL	8	Follow up	10.1016/j.jadohealth.2020.07.012
Using Mobile Health to Improve Asthma Self-Management in Early Adolescence: A Pilot Randomized Controlled Trial	Fedele DA, Thomas JG, McConville A, McQuaid EL, Voorhees S, Janicke DM, Abu-Hasan M, Chi X, Gurka MJ.	United States of America	North America	Article	2021	Asthma	Asthma	Interventional studies	J Adolesc Health. 2021 Dec69(6):1032-1040	JOURNAL OF ADOLESCENT HEALTH	4,828	PSYCHOLOGY, DEVELOPMENTAL	2	Trearment	10.1016/j.jadohealth.2021.06.011
Pilot Executive Functioning Intervention in Epilepsy: Behavioral and Quality of Life Outcomes	Modi A.C., Mara C.A., Schmidt M., Smith A.W., Turnier L., Wade S.L.	United States of America	North America	Article	2021	Epilepsy	Brain disorders	Interventional studies	Journal of pediatric psychology (2021) 46:4 (363-374)	JOURNAL OF PEDIATRIC PSYCHOLOGY	3,191	PSYCHOLOGY, DEVELOPMENTAL	1	Trearment	10.1093/jpepsy/jsaa119
Telehealth cognitive behavioral therapy for insomnia in children with autism spectrum disorder: A pilot examining feasibility, satisfaction, and preliminary findings	McCrae CS, Chan WS, Curtis AF, Nair N, Deroche CB, Munoz M, Takamatsu S, McLean D, Davenport M, Muckerman JE, Takahashi N, McCann D, McGovney K, Sahota P, Mazurek MO.	United States of America	North America	Article	2021	Autism spectrum disorders	Neurodevelopmental diseases or disorders	Interventional studies	Autism. 2021 Apr25(3):667-680	AUTISM	5,689	PSYCHOLOGY, DEVELOPMENTAL	11	Trearment	10.1177/1362361320949078
Factors related to parental therapeutic self-efficacy in a parent-mediated intervention for children with autism spectrum disorder: A mixed methods study	Russell KM, Ingersoll B.	United States of America	North America	Article	2021	Autism spectrum disorders	Neurodevelopmental diseases or disorders	Interventional studies	Autism. 2021 May25(4):971-981	AUTISM	5,689	PSYCHOLOGY, DEVELOPMENTAL	7	Trearment	10.1177/1362361320974233
Leveraging telehealth to evaluate infants with prodromal autism spectrum disorder characteristics using the telehealth evaluation of development for infants	Talbott M.R., Dufek S., Young G., Rogers S.J.	United States of America	North America	Article	2021	Autism spectrum disorders	Neurodevelopmental diseases or disorders	Observational studies	Autism (2021). Date of Publication: 2021	AUTISM	5,689	PSYCHOLOGY, DEVELOPMENTAL	0	Diagnosis	10.1177/13623613211045596

Quality of unsupervised home spirometry in children with asthma	Agerskov N., Coughlin S., Parrott H., Saglani S., Sonnappa S., Fleming L.	Sweden	Europe	Conference abstract	2021	Asthma	Asthma	Interventional studies	American Journal of Respiratory and Critical Care Medicine (2021) 203:9	AMERICAN JOURNAL OF RESPIRATORY AND CRITICAL CARE MEDICINE	4,09	RESPIRATORY SYSTEM	0	Trearment	10.1164/ajrccm-conference.2021.203.1_MeetingAbstracts.A1088
Telemedicine and adherence monitoring in children with asthma	Blake KV.	United States of America	North America	Article	2021	Asthma	Asthma	Review/meta-analysis	Curr Opin Pulm Med. 2021 Jan27(1):37-44	CURRENT OPINION IN PULMONARY MEDICINE	3,155	RESPIRATORY SYSTEM	4	Follow up	10.1097/MCP.0000000000000739
Detection of asthma exacerbation in adolescent and adult subjects with chronic asthma using a cough-centred, smartphone based algorithm	Claxton S.	Australia	Oceania	Conference abstract	2021	Asthma	Asthma	Observational studies	Respirology (2021) 26:SUPPL 2 (85)	RESPIROLOGY	6,424	RESPIRATORY SYSTEM	0	Follow up	10.1111/resp.14021
School nurse perception of asthma care in school-based telehealth	MacGeorge, C. A.; King, K.; Andrews, A. L.; Sterba, K.; Johnson, E.; Brinton, D. L.; Teufel, R. J.; Kruis, R.; Ford, D.	United States of America	North America	Article	2022	Asthma	Asthma	Observational studies	J Asthma. 2022 Jun;59(6):1248-1255.	JOURNAL OF ASTHMA	1,899	ALLERGY	3	Trearment	10.1080/02770903.2021.1904978
Telemedicine use for pediatric asthma care: a mixed methods study	Haynes, SC; Kamerman-Kretzmer, R; Khan, SS; Crossen, S; Lieng, MK; Marcin, JP; Kenyon, NJ; Kim, CH	United States of America	North America	Article	2022	Asthma	Asthma	Interventional studies	(2022) Journal of Asthma	JOURNAL OF ASTHMA	2,515	ALLERGY	0	Trearment	10.1080/02770903.2021.2019265
A trial of online ABRACADABRA literacy instruction with supplementary parent-led shared book reading for children with autism	Bailey, B.; Sellwood, D.; Rillotta, F.; Raghavendra, P.; Arciuli, J.	Australia	Oceania	Article	2022	Autism spectrum disorders	Neurodevelopmental diseases or disorders	Interventional studies	Res Dev Disabil. 2022 May;124:104198	RESEARCH IN DEVELOPMENTAL DISABILITIES	3,23	EDUCATION, SPECIAL	0	Prevention	10.1016/j.ridd.2022.104198
IDF21-0301 Bridging the Needs of Adolescent Diabetes Care during Coronavirus Disease 2019: A Nurse-Led Telehealth Initiative	Lim, S. T.	Singapore	Asia	Article	2022	Diabetes mellitus type I	Metabolic diseases	Observational studies	J Adolesc Health. 2020 Oct;67(4):615-617.	DIABETES RESEARCH AND CLINICAL PRACTICE	5,602	ENDOCRINOLOGY & METABOLISM	0	Follow up	10.1016/j.diabres.2022.109335
The endorse feasibility pilot trial: assessing the implementation of serious games strategy and artificial intelligence-based telemedicine in glycemic control improvement	Vasilakis, I. A.; Kosteria, I.; Mitsis, K.; Zarkogianni, K.; Kalafatis, L.; Athanasiou, M.; Perakis, K.; Papavasiliou, G.; Taliou, A.; Nicolaides, N.; Chioti, V.; Tokou, I.; Vergeti, D.; Antonopoulou, D.; Papachristou, E.; Meklis, V.; Nikita, K.; Kanaka-Gantenbein, C.	Greece	Europe	conference abstract	2022	Diabetes mellitus type I	Metabolic diseases	Interventional studies	Diabetes Technology and Therapeutics ; 24(SUPPL 1):A218-A219, 2022	DIABETES TECHNOLOGY AND THERAPEUTICS	6,118	ENDOCRINOLOGY & METABOLISM	0	Follow up	10.1177/2164957X221096590

Remote patient monitoring in youth with type 1 diabetes (t1d) predicted to experience a rise in a1c%: comparison to a clinic-derived, propensity score-matched controls	Williams, D.; Ferro, D.; Dewit, E.; Lockee, B.; Barnes, M.; Carrothers, S.; Vandervelden, C.; Patton, S.; McDonough, R.; D'Avolio, L.; Clements, M.	United States of America	North America	Conference abstract	2022	Diabetes mellitus type I	Metabolic diseases	Study protocol/Pilot study	NA	DIABETES TECHNOLOGY AND THERAPEUTICS	6,118	ENDOCRINOLOGY & METABOLISM	0	Follow up	10.1177/2164957X221096590
INITIATING CGM OVER TELEHEALTH IS WELL ACCEPTED BY PARENTS OF NEWLY DIAGNOSED YOUTH WITH T1D	Tanenbaum, M.; Zaharieva, D.; Addala, A.; Hooper, J.; Leverenz, B.; Cortes, A.; Arrizon-Ruiz, N.; Pang, E.; Bishop, F.; Maahs, D.	United States of America	North America	Conference abstract	2022	Diabetes mellitus type I	Metabolic diseases	Interventional studies	Tanenbaum, M., Zaharieva, D., Addala, A., Hooper, J., Leverenz, B., Cortes, A., ... & Maahs, D. (2022). INITIATING CGM OVER TELEHEALTH IS WELL ACCEPTED BY PARENTS OF NEWLY DIAGNOSED YOUTH WITH T1D. Diabetes Technology and Therapeutics, A157-A157.	DIABETES TECHNOLOGY AND THERAPEUTICS	6,118	ENDOCRINOLOGY & METABOLISM	0	Follow up	10.1177/2164957X221096590
Empowering Patients with Type 1 Diabetes Through a Multidisciplinary Team-assisted, Technology-Enabled Education Program	Sanal, G.; Shijin, S.; Krishna, V.; Kesavadev, J.; Basanth, A.; Krishnan, G.; Shankar, A.	India	Asia	Article	2022	Diabetes mellitus type I	Metabolic diseases	Interventional studies	Curr Diabetes Rev. 2022 May 20	CURRENT DIABETES REPORTS	4,813	ENDOCRINOLOGY & METABOLISM	0	Trearment	10.2174/1573399818666220520115420
Psychosocial Needs for Newly Diagnosed Youth with Type 1 Diabetes and Their Families	Patton, S. R.; Maahs, D.; Prahalad, P.; Clements, M. A.	United States of America	North America	Article	2022	Diabetes mellitus type I	Metabolic diseases	review/meta-analysis	Curr Diab Rep. 2022 Aug;22(8):385-392.	CURRENT DIABETES REPORTS	4,813	ENDOCRINOLOGY & METABOLISM	0	Trearment	10.1007/s11892-022-01479-8
A Nonrandomized Pilot of a Group, Video-Based Telehealth Intervention to Reduce Diabetes Distress in Parents of Youth With Type 1 Diabetes Mellitus	Patton, S. R.; Monzon, A. D.; Marker, A. M.; Clements, M. A.	United States of America	North America	Article	2022	Diabetes mellitus type I	Metabolic diseases	Observational studies	Can J Diabetes. 2022 Apr;46(3):262-268.	CANADIAN JOURNAL OF DIABETES	4,19	ENDOCRINOLOGY & METABOLISM	1	Follow up	10.1016/j.jcjd.2021.10.007
Diabetes Technology Use in Remote Pediatric Patients with Type 1 Diabetes Using Clinic-to-Clinic Telemedicine	Cobry, E. C.; Reznick-Lipina, T.; Pyle, L.; Slover, R.; Thomas, J. F.; Alonso, G. T.; Wadwa, R. P.	United States of America	North America	Article	2022	Diabetes mellitus type I	Metabolic diseases	Observational studies	Diabetes Technol Ther. 2022 Jan;24(1):67-74.	DIABETES TECHNOLOGY & THERAPEUTICS	6,118	ENDOCRINOLOGY & METABOLISM	1	Follow up	10.1089/dia.2021.0229

Telemedicine follow-up of adolescents with type 1 diabetes mellitus with one touch reveal (r) mobile app	Laptev, D.; Eremina, I.; Petryaykina, E.; Bezlepkina, O.; Peterkova, V.	Russia	Europe	Conference abstract	2022	Diabetes mellitus type I	Metabolic diseases	Interventional studies	Global Advances in Health and Medicine. January 2022.	DIABETES TECHNOLOGY & THERAPEUTICS	6,118	ENDOCRINOLOGY & METABOLISM	0	Follow up	10,1177/2164957X221096590
Improvement in Mean CGM Glucose in Young People with Type 1 Diabetes During 1 Year of the COVID-19 Pandemic	Kaushal, T.; Tinsley, L.; Volkening, L. K.; Ambler-Osborn, L.; Laffel, L.	United States of America	North America	Article	2022	Diabetes mellitus type I	Metabolic diseases	Interventional studies	Diabetes Technol Ther. 2022 Feb;24(2):136-139.	DIABETES TECHNOLOGY & THERAPEUTICS	6,118	ENDOCRINOLOGY & METABOLISM	1	Follow up	10.1089/dia.2021.0258
Hba1c determinations according to telemedicine access pre and post lockdown in latin american children with type 1 diabetes	Hirschler, V.; Molinari, C.; Pelicand, J.; Figueroa Sobrero, A.; Ibarcena, P. Pinto; Del Aguila Villar, C.; Scaiola, E.; Bocco, P.; Gonzalez, D.; Mac, A.; Ramirez Trillo, C.; Lapertosa, S.; Gonzalez, C.	Argentina	South America	Article	2022	Diabetes mellitus type I	Metabolic diseases	Interventional studies	Diabetes Technology and Therapeutics 2022 24:SUPPL 1 (A30-A31)	DIABETES TECHNOLOGY & THERAPEUTICS	6,118	ENDOCRINOLOGY & METABOLISM	0	Follow up	10.1177/2164957X221096590
Active & passive sharing of diabetes device data to clinics is associated with reduced a1c and decreased dka rates	McDonough, R.; Ferro, D.; Lockee, B.; Clements, M.	United States of America	North America	Conference abstract	2022	Diabetes mellitus type I	Metabolic diseases	Interventional studies	Diabetes Technology and Therapeutics 2022 24:SUPPL 1 (A44-A45)	DIABETES TECHNOLOGY & THERAPEUTICS	6,118	ENDOCRINOLOGY & METABOLISM	0	Follow up	10.1177/2164957X221096590
Diabetes device data in virtual clinic visits: a new health disparty?	McDonough, R.; Ferro, D.; Lockee, B.; Clements, M.	United States of America	North America	Conference abstract	2022	Diabetes mellitus type I	Metabolic diseases	Interventional studies	NA	DIABETES TECHNOLOGY & THERAPEUTICS	6,118	ENDOCRINOLOGY & METABOLISM	0	Follow up	10.1177/2164957X221096590
Telemedicin a tool for the treatment of new onset type 1 diabetes in pediatrics: two year follow-up in argentine patagonia	Grabois, F.; Cruz, C.; Herrera, A.; Puliafito, T.; Carnaccini, L.; Casullo, M.	United States of America	North America	Article	2022	Diabetes mellitus type I	Metabolic diseases	Interventional studies	Diabetes Technology and Therapeutics 2022 24:SUPPL 1 (A150-)	DIABETES TECHNOLOGY & THERAPEUTICS	6,118	ENDOCRINOLOGY & METABOLISM	0	Trearment	10.1177/2164957X221096590
eP480: Project ECHO for pediatric genetics in Mississippi: Expanding access to the clinical genetics workup for autism and intellectual disability	Boothe, E.; Waldrop, J. M.; Kirmse, B.	United States of America	North America	Article	2022	Autism spectrum disorders	Neurodevelopmental diseases or disorders	Study protocol/Pilot study	Genetics in Medicine Volume 24, Issue 3, Supplement, March 2022, Page S306	GENETICS IN MEDICINE	8,822	GENETICS & HEREDITY	0	Diagnosis & Treatment/Follow-up	10.1016/j.gim.2022.01.512
eP510: High patient satisfaction with specialty pediatric services using telemedicine during the COVID-19 pandemic	Schwoerer, J. S.; Zoran, S.; Whitehead, A.; Hrabik, L.; Harris, A.; Stanley, M.; Turcott, C.	United States of America	North America	Article	2022	Autism spectrum disorders	Neurodevelopmental diseases or disorders	Observational studies	Genet Med. 2022 Mar;24(3):S325.	GENETICS IN MEDICINE	8,822	GENETICS & HEREDITY	0	Follow up	10.1016/j.gim.2022.01.542

Rapid Adoption of Telemedicine Along with Emergent Use of Continuous Glucose Monitors in the Ambulatory Care of Young Persons with New-Onset Type 1 Diabetes in the Time of COVID-19: A Case Series	Kaushal, T.; Ambler-Osborn, L.; Turcotte, C.; Quinn, H.; Laffel, L.	United States of America	North America	Article	2022	Diabetes mellitus type I	Metabolic diseases	Study protocol/Pilot study	Telemed J E Health. 2022 Jan;28(1):107-114.	TELEMEDICINE JOURNAL AND E-HEALTH	NA	HEALTH CARE SCIENCES & SERVICES	1	Follow up	10.1089/tmj.2020.0554
Acceptance of Telehealth Therapy to Replace In-Person Therapy for Autism Treatment During COVID-19 Pandemic: An Assessment of Patient Variables	Aranki, J.; Wright, P.; Pompa-Craven, P.; Lotfizadeh, A. D.	United States of America	North America	Article	2022	Autism spectrum disorders	Neurodevelopmental diseases or disorders	Observational studies	Telemed J E Health. 2022 Feb 3	TELEMEDICINE JOURNAL AND E-HEALTH : THE OFFICIAL JOURNAL OF THE AMERICAN TELEMEDICINE ASSOCIATION	NA	HEALTH CARE SCIENCES & SERVICES	0	Trearment	10.1089/tmj.2021.0397
Sensor-Based Technology: Bringing Value to People with Diabetes and the Healthcare System in an Evolving World	Glennie, Judith L.; Berard, Lori; Levrat-Guillen, Fleur	multicenter	More than one continent	Article	2022	Diabetes mellitus type I	Metabolic diseases	Observational studies	Clinicoecon Outcomes Res. 2022 Feb 10;14:75-90.	CLINICOECONOMICS AND OUTCOMES RESEARCH	0,6	HEALTH CARE SCIENCES & SERVICES	1	Follow up	10.2147/CEOR. S346736
The impact of Covid-19 pandemic on services for children and adolescents with ADHD: results from a survey of paediatricians in the United Kingdom	Ogundele, Michael; Ayyash, Hani F.; Ani, Cornelius	United Kingdom	Europe	Article	2022	Attention-deficit/hyperactivity disorder	Neurodevelopmental diseases or disorders	Observational studies	AIMS Public Health, 2022, 9(3): 542-551.	AIMS PUBLIC HEALTH	0,34	HEALTH CARE SCIENCES & SERVICES	0	Trearment	doi: 10.3934/publichealth.2022037
Telehealth Education via WeChat Improves the Quality of Life of Parents of Children with Type-1 Diabetes Mellitus	Huang, M. X.; Wang, M. C.; Wu, B. Y.	China	Asia	Article	2022	Diabetes mellitus type I	Metabolic diseases	Interventional studies	Appl Clin Inform. 2022 Jan;13(1):263-269.	APPLIED CLINICAL INFORMATICS	2,342	MEDICAL INFORMATICS	0	Follow up	10.1055/s-0042-1743239
The accuracy of teledentistry in caries detection in children - A diagnostic study	AlShaya, Mohammad; Farsi, Deema; Farsi, Nada; Farsi, Najat	Saudi Arabia	Asia	Article	2022	Caries of deciduous teeth	Dental caries	Observational studies	Digit Health. 2022 Jun 22;8:20552076221109075	DIGITAL HEALTH	3,495	MEDICAL INFORMATICS	0	Diagnosis	10.1177/20552076221109075
Impact of virtual care on health-related quality of life in children with diabetes mellitus: a systematic review protocol	Rajan, Raeesha; Kshatriya, Maya; Banfield, Laura; Athale, Uma; Thabane, Lehana; Samaan, M. Constantine	Canada	North America	Article	2022	Diabetes mellitus type I	Metabolic diseases	Study protocol/Pilot study	BMJ Open. 2022 Feb 16;12(2):e053642.	BMJ OPEN	2,692	MEDICINE, GENERAL & INTERNAL	0	Follow up	0.1136/bmjopen-2021-053642.
Expectations and Concerns about the Use of Telemedicine for Autism Spectrum Disorder: A Cross-Sectional Survey of Parents and Healthcare Professionals	Gabellone, Alessandra; Marzulli, Lucia; Matera, Emilia; Petruzzelli, Maria Giuseppina; Margari, Anna; Giannico, Orazio Valerio; Margari, Lucia	Italy	Europe	Article	2022	Autism spectrum disorders	Neurodevelopmental diseases or disorders	Observational studies	J Clin Med. 2022 Jun 8;11(12):3294	JOURNAL OF CLINICAL MEDICINE	4,242	MEDICINE, GENERAL & INTERNAL	0	Follow up	doi: 10.3390/jcm11123294

Using mHealth to promote parents' brushing of preschool children's teeth: a protocol for a randomized factorial trial using the Multi-phase Optimization Strategy (MOST)	Ihab, M.; El Din, W. E.; Ammar, N.; Yassin, R.; El Tantawi, M.	Egypt	Africa	Article	2022	Caries of deciduous teeth	Dental caries	Study protocol/Pilot study	Trials. 2022 Jan 6;23(1):17.	TRIALS	2,279	MEDICINE, RESEARCH & EXPERIMENTAL	1	Prevention	10.1186/s13063-021-05931-0
Analysis of "Accuracy of a 14-Day Factory Calibrated Continuous Glucose Monitoring System With Advanced Algorithm in Pediatric and Adult Population With Diabetes"	Kompala, T.; Neinstein, A. B.	United States of America	North America	Comment/editorial/letter	2022	Diabetes mellitus type I	Metabolic diseases	Review/meta-analysis	J Diabetes Sci Technol. 2022 Jan;16(1):78-80.	JOURNAL OF DIABETES SCIENCE AND TECHNOLOGY	NA	NA	1	Trearment	10.1177/19322968209670 04
Remote Intensive Parent-Implemented Intervention for Young Children on the Autism Spectrum During Covid-19: The Experience of Parents and Therapists	de Wilde, H. W.; Kojovic, N.; Robertson, C.; Karr, C.; Akman, L.; Caccia, F.; Costes, A.; Etienne, M.; Franchini, M.; Gentaz, E.; Schaer, M.	Switzerland	Europe	Article	2022	Autism spectrum disorders	Neurodevelopmental diseases or disorders	Interventional studies	NA	MEDRXIV	NA	NA	0	Follow up	NA
Extended Reality (XR) and telehealth interventions for children or adolescents with autism spectrum disorder: Systematic review of qualitative and quantitative studies	Chen, Y.; Zhou, Z.; Cao, M.; Liu, M.; Lin, Z.; Yang, W.; Yang, X.; Dhaidhai, D.; Xiong, P.	China	Asia	Article	2022	Autism spectrum disorders	Neurodevelopmental diseases or disorders	Review/meta-analysis	Neurosci Biobehav Rev. 2022 Jul;138:104683	NEUROSCIENCE AND BIOBEHAVIORAL REVIEWS	8,989	NEUROSCIENCES	1	Follow up	10.1016/j.neubiorev.2022.104683
Integrating a New Online Platform in Primary Care for Early Detection, Referral, and Intervention in Autism Spectrum Disorder: The First Italian Pivotal Clinical Study	Colombo, Paola; Buo, Noemi; Busti Ceccarelli, Silvia; Molteni, Massimo	Italy	Europe	Article	2022	Autism spectrum disorders	Neurodevelopmental diseases or disorders	Observational studies	Brain Sci. 2022 Feb 12;12(2):256	BRAIN SCIENCES	3,394	NEUROSCIENCES	1	Diagnosis & Treatment/Follow-up	10.3390/brainsci12020256
Asthma academy: A student nurse-led telehealth education program for low-income family caregivers of children with asthma	Foronda, C.; Prather, S.; Snowden, K.; Gonzalez, J. M.; Gattamorta, K. A.; Lee, J.; Gonzalez, J. E.; Cardenas, M.	United States of America	North America	Article	2022	Asthma	Asthma	Observational studies	Nurs Open. 2022 Mar;9(2):1486-1496	NURSING OPEN	1,762	NURSING	0	Follow up	10.1002/nop2.1123
Pediatric apps: what are they for? A scoping review	Nievas Soriano, B. J.; Uribe-Toril, J.; Ruiz-Real, J. L.; Parrón-Carreño, T.	Spain	Europe	Article	2022	Asthma	Asthma	Review/meta-analysis	Eur J Pediatr. 2022 Apr;181(4):1321-1327.	EUROPEAN JOURNAL OF PEDIATRICS	3,183	PEDIATRICS	0	Diagnosis & Treatment/Follow-up	10.1007/s00431-021-04351-1
The Promise of School-Based Asthma Interventions	Hollenbach, Jessica P.; Simoneau, Tregony; Halterman, Jill Suzanne	United States of America	North America	Article	2022	Asthma	Asthma	Review/meta-analysis	Acad Pediatr. 2022 Apr;22(3):385-386.	ACADEMIC PEDIATRICS	3,107	PEDIATRICS	0	Trearment	10.1016/j.acap.2021.11.006

Guardian-Reported Impact of the COVID-19 Pandemic on the Lifestyle of Children with Diabetes Mellitus	Vyas, V.; Singh, K.; Pareek, P.; Garg, M. K.; Didel, S.; Priyanka, P.; Goel, A. D.; Misra, S.	India	Asia	Article	2022	Diabetes mellitus type I	Metabolic diseases	Observational studies	J Trop Pediatr. 2022 Feb 3;68(2):fmac013.	JOURNAL OF TROPICAL PEDIATRICS	1,165	PEDIATRICS	0	Follow up	10.1093/tropej/fmac013
Visits of concern in child neurology telemedicine	Prelack, M.; Fridinger, S.; Gonzalez, A. K.; Kaufman, M. C.; Xian, J.; Galer, P. D.; Craig, S.; Abend, N. S.; Helbig, I.; Rametta, S. C.; Sharif, U.; Szperka, C.; Chadehumbe, M.; Fitzgerald, M. P.; Chuo, J.; Melamed, S. E.; Malcolm, M. P.; Kessler, S. K.; Banwell, B. L.; Stephenson, D.	United States of America	North America	Article	2022	Epilepsy	Brain disorders	Observational studies	Dev Med Child Neurol. 2022 May 5.	DEVELOPMENTAL MEDICINE AND CHILD NEUROLOGY	5,449	PEDIATRICS	0	Follow up	10.1111/dmcn.15256
When is synchronous telehealth acceptable for pediatric dermatology?	Kohn, L. L.; Pickett, K.; Day, J. A.; Torres-Zegarra, C.; Plost, G.; Gurnee, E.; Prok, L.; Olson, C. A.; Manson, S. M.; Bruckner, A. L.	United States of America	North America	Article	2022	Acne vulgaris	Skin diseases	Interventional studies	Pediatr Dermatol. 2022 Mar;39(2):236-242.	PEDIATRIC DERMATOLOGY	1,588	PEDIATRICS	1	Follow up	10.1111/pde.14919
COVID-19 and delivery of difficult asthma services	Nichols, A. L.; Sonnappa-Naik, M.; Gardner, L.; Richardson, C.; Orr, N.; Jamalzadeh, A.; Moore-Crouch, R.; Makhecha, S.; Wells, C.; Hall, P.; Bush, A.; Fleming, L.; Saglani, S.; Sonnappa, S.	United Kingdom	Europe	Article	2022	Asthma	Asthma	Interventional studies	Arch Dis Child. 2022 Mar;107(3):e15.	ARCHIVES OF DISEASE IN CHILDHOOD	3,801	PEDIATRICS	0	Trearment	10.1136/archdischild-2021-322335
mHealth apps delivering early intervention to support parents of children with autism: A scoping review protocol	Bharat, R.; Uzaina, U.; Yadav, T.; Niranjan, S.; Kurade, P.	India	Asia	Article	2022	Autism spectrum disorders	Neurodevelopmental diseases or disorders	Review/meta-analysis	NA	BMJ PAEDIATRICS OPEN	NA	PEDIATRICS	0	Follow up	10.1136/bmjpo-2021-001358
ECHO Autism: Early Intervention Connecting Community Professionals to Increase Access to Best Practice Autism Intervention	Buranova, N.; Dampf, M.; Stevenson, B.; Sohl, K.	United States of America	North America	Article	2022	Autism spectrum disorders	Neurodevelopmental diseases or disorders	Interventional studies	Clin Pediatr (Phila). 2022 Sep;61(8):518-522	CLINICAL PEDIATRICS	1,168	PEDIATRICS	0	Follow up	10.1177/00099228221090710
Patient Satisfaction in Neurodevelopmental Pediatrics: In-Person vs Telemedicine	Kennelly, Ann M.; McIntyre, Brandon; Wood, Alexis C.; Monteiro, Sonia; Voigt, Robert G.	United States of America	North America	Article	2022	Autism spectrum disorders	Neurodevelopmental diseases or disorders	Observational studies	J Child Neurol. 2022 Mar;37(3):181-185.	JOURNAL OF CHILD NEUROLOGY	1,987	PEDIATRICS	0	Trearment	10.1177/08830738221075451

Application of Telemedicine for Preliminary Screening of Autism Spectrum Disorder	Qiu, Ting; Zhang, Heng; Zhou, Conghua; Tang, Qilong; Wang, Lizhen; Ke, Xiaoyan	China	Asia	Article	2022	Autism spectrum disorders	Neurodevelopmental diseases or disorders	interventional studies	Front Pediatr. 2022 Jan 18;9:745597.	FRONTIERS IN PEDIATRICS	3,418	PEDIATRICS	0	diagnosis	10.3389/fped.2021.745597
Lung Function Tests, Quality of Life and Telemedicine: Three Windows on the Multifaceted World of Asthma in Adolescents	Nucera, Eleonora; Rizzi, Angela; Agrosi, Chiara; Lohmeyer, Franziska Michaela; Inchingolo, Riccardo	Italy	Europe	Article	2022	Asthma	Asthma	Review/meta-analysis	Children (Basel). 2022 Mar 30;9(4):476.	CHILDREN-BASEL	2,863	PEDIATRICS	0	Diagnosis & Treatment/Follow-up	10.3390/children9040476.
A scoping review of mHealth monitoring of pediatric bronchial asthma before and during COVID-19 pandemic	Dauletbaev, N.; Oftring, Z. S.; Akik, W.; Michaelis-Braun, L.; Korel, J.; Lands, L. C.; Waldmann, S.; Müller, B. S.; Dreher, M.; Rohde, G.; Vogelmeier, C. F.; Kuhn, S.	United States of America	North America	Article	2022	Asthma	Asthma	Review/meta-analysis	Paediatr Respir Rev. 2022 Jan 17;S1526-0542(22)00002-1	PAEDIATRIC RESPIRATORY REVIEWS	2,726	PEDIATRICS	0	Follow up	10.1016/j.prrv.2022.01.002
Primary Care Diagnosis and Treatment of Attention-Deficit/Hyperactivity Disorder in School-Age Children: Trends and Disparities During the COVID-19 Pandemic	Bannett, Y.; Dahlen, A.; Huffman, L. C.; Feldman, H. M.	United States of America	North America	Article	2022	Attention-deficit/hyperactivity disorder	Neurodevelopmental diseases or disorders	Observational studies	J Dev Behav Pediatr. 2022 May 2	JOURNAL OF DEVELOPMENTAL AND BEHAVIORAL PEDIATRICS : JDBP	2,225	PEDIATRICS	0	Diagnosis & Treatment/Follow-up	10.1097/DBP.0000000000001087
Using Telehealth to Conduct Family-Centered, Movement Intervention Research in Children With Autism Spectrum Disorder During the COVID-19 Pandemic	Cleffi, C.; Su, W. C.; Srinivasan, S.; Bhat, A.	United States of America	North America	Article	2022	Autism spectrum disorders	Neurodevelopmental diseases or disorders	Study protocol/Pilot study	Pediatr Phys Ther. 2022 Apr 1;34(2):246-251.	PEDIATRIC PHYSICAL THERAPY : THE OFFICIAL PUBLICATION OF THE SECTION ON PEDIATRICS OF THE AMERICAN PHYSICAL THERAPY ASSOCIATION	3,049	PEDIATRICS	2	Follow up	10.1097/PEP.0000000000000872
Asthma Management in the Era of the COVID-19 Pandemic	Klouda, T.; Pillarisetti, A.; Xie, A.; Kabra, S.; Saradhi, N.; Katwa, U.	multicenter	More than one continent	Article	2022	Asthma	Asthma	Review/meta-analysis	Indian J Pediatr. 2022 Feb;89(2):163-168.	INDIAN JOURNAL OF PEDIATRICS	1,967	PEDIATRICS	0	Follow up	10.1007/s12098-021-03979-z
Bring Blood Glucose Down! An intervention to reduce fear of hypoglycemia in caregivers of adolescents with type 1 diabetes: Study design and participant characteristics	O'Donnell, H. K.; Vigers, T.; Johnson, S. B.; Pyle, L.; Gonder-Fredrick, L.; Hendrieckx, C.; Driscoll, K. A.	United States of America	North America	Article	2022	Diabetes mellitus type I	Metabolic diseases	Interventional studies	Contemp Clin Trials. 2022 Jul;118:106792	CONTEMPORARY CLINICAL TRIALS	2,226	PHARMACOLOGY & PHARMACY	0	Follow up	10.1016/j.cct.2022.106792

A Solution Focused Approach of Delivering Virtual Paediatric Diabetes Consultations During the COVID-19 Pandemic	Woodger K.,Bray D.,Welsh C.,Ng S.M.	United Kindom	Europe	Article	2022	Diabetes mellitus type I	Metabolic diseases	Observational studies	Clin Child Psychol Psychiatry. 2022;27(1):177-184	CLINICAL CHILD PSYCHOLOGY AND PSYCHIATRY	2,544	PSYCHIATRY	1	Follow up	10.1177/135910452110583 36
Home direct-to-consumer telehealth solutions for children with mental health disorders and the impact of Covid-19	Norman, S.; Atabaki, S.; Atmore, K.; Biddle, C.; DiFazio, M.; Felten, D.; Fox, E.; Marschall, D.; Newman, J.; Robb, A.; Rowland, C.; Selekman, R.; Slovin, A.; Stein, M.; Strang, J.; Sable, C.	United States of America	North America	Article	2022	Major depressiv e disorder	Mental disorders	review/meta-analysis	Clin Child Psychol Psychiatry. 2022 Jan;27(1):244-258	CLINICAL CHILD PSYCHOLOGY AND PSYCHIATRY	2,544	PSYCHIATRY	1	Diagnosis & Treatment/Follow-up	10.1177/135910452110641 34
Telehealth Versus Face-to-face Psychotherapy for Less Common Mental Health Conditions: Systematic Review and Meta-analysis of Randomized Controlled Trials	Greenwood, Hannah; Krzyzaniak, Natalia; Peiris, Ruwani; Clark, Justin; Scott, Anna Mae; Cardona, Magnolia; Griffith, Rebecca; Glasziou, Paul	Australia	Oceania	Article	2022	Major depressiv e disorder	Mental disorders	Review/meta-analysis	JMIR Ment Health. 2022 Mar 11;9(3):e31780.	JMIR MENTAL HEALTH	4,388	PSYCHIATRY	2	Trearment	10.2196/3178 0
Autism Diagnostic Assessments With Children, Adolescents, and Adults Prior to and During the COVID-19 Pandemic: A Cross-Sectional Survey of Professionals	Spain, D.; Stewart, G. R.; Mason, D.; Robinson, J.; Capp, S. J.; Gillan, N.; Ensum, I.; Happé, F.	United Kingdom	Europe	Article	2022	Autism spectrum disorders	neurodevelopmental diseases or disorders	observational studies	Front Psychiatry. 2022 Apr 28;13:789449.	FRONTIERS IN PSYCHIATRY	4,157	PSYCHIATRY	1	Diagnosis & Treatment/Follow-up	10.3389/fpsyt.2022.789449
Mental health of children with neurodevelopmental disorders during COVID-19: A brief report of family experiences from a low and middle income country	Kaku SM.	India	Asia	Article	2022	Attention-deficit/hyperactivit y disorder	Neurodevelopmenta l diseases or disorders	Observational studies	Clin Child Psychol Psychiatry. 2022 Jan;27(1):269-277	CLINICAL CHILD PSYCHOLOGY AND PSYCHIATRY	2,544	PSYCHIATRY	0	Trearment	10.1177/135910452110260 58
Treatment Interruptions and Telemedicine Utilization in Serious Mental Illness: Retrospective Longitudinal Claims Analysis	Ainslie, M.; Brunette, M. F.; Capozzoli, M.	United States of America	Europe	Article	2022	Major depressiv e disorder	Mental disorders	Observational studies	JMIR Ment Health. 2022 Mar 21;9(3):e33092	JMIR MENTAL HEALTH	4,388	PSYCHIATRY	0	Follow up	10.2196/3309 2
Early Detection of Neurodevelopmental Disorders of Toddlers and Postnatal Depression by Mobile Health App: Observational Cross-sectional Study	Denis, F.; Maurier, L.; Carillo, K.; Ologeanu-Taddei, R.; Septans, A. L.; Gepner, A.; Le Goff, F.; Desbois, M.; Demurger, B.; Silber, D.; Zeitoun, J. D.; Assuied, G. P.; Bonnot, O.	France	Europe	Article	2022	Major depressiv e disorder	Mental disorders	Observational studies	JMIR Mhealth Uhealth. 2022 May 16;10(5):e3818	JMIR MENTAL HEALTH	4,388	PSYCHIATRY	0	Follow up	10.2196/3818 1

The effective delivery of digital CBT: a service evaluation exploring the outcomes of young people who completed video conferencing therapy in 2020	Porter, Catherine M.; Galloghly, Emily; Burbach, Frank R.	United Kingdom	Europe	Article	2022	Major depressive disorder	Mental disorders	Interventional studies	The Cognitive Behaviour Therapist, 15, E27.	COGNITIVE BEHAVIOUR THERAPIST	0,58	PSYCHOLOGY, CLINICAL	0	Trearment	10.1017/S1754470X22000216
A Retrospective Analysis of Therapists' Coaching Behavior When Directing Parents to Conduct Behavioral Assessments and Treatments Via Telehealth	Larsen, A.; Schieltz, K. M.; Barrett, A.; O'Brien, M. J.	United States of America	North America	Article	2022	Autism spectrum disorders	Neurodevelopmental diseases or disorders	Observational studies	. Behav Modif. 2022 Jun 22:1454455221106127.	BEHAVIOR MODIFICATION	3,368	PSYCHOLOGY, CLINICAL	0	Trearment	10.1177/01454455221106127
Characterizing Available Tools for Synchronous Virtual Assessment of Toddlers with Suspected Autism Spectrum Disorder: A Brief Report	Berger, N. I.; Wainer, A. L.; Kuhn, J.; Bearss, K.; Attar, S.; Carter, A. S.; Ibanez, L. V.; Ingersoll, B. R.; Neiderman, H.; Scott, S.; Stone, W. L.	United States of America	North America	Article	2022	Autism spectrum disorders	Neurodevelopmental diseases or disorders	Observational studies	J Autism Dev Disord. 2022 Jan;52(1):423-434	JOURNAL OF AUTISM AND DEVELOPMENTAL DISORDERS	4,291	PSYCHOLOGY, DEVELOPMENTAL	16	Diagnosis & Treatment/Follow-up	10.1007/s10803-021-04911-2
COVID-19 Pandemic and Impact on Patients with Autism Spectrum Disorder	Baweja, R; Brown, SL; Edwards, EM; Murray, MJ	United States of America	North America	Comment/editorial/letter	2022	Autism spectrum disorders	Neurodevelopmental diseases or disorders	Review/meta-analysis	J Autism Dev Disord. 2022 Jan;52(1):473-482	JOURNAL OF AUTISM AND DEVELOPMENTAL DISORDERS	4,291	PSYCHOLOGY, DEVELOPMENTAL	20	Trearment	10.1007/s10803-021-04950-9
Preliminary Validation and Feasibility of the Autism Detection in Early Childhood-Virtual (ADEC-V) for Autism Telehealth Evaluations in a Hospital Setting	Kryszak, E. M.; Albright, C. M.; Stephenson, K. G.; Nevill, R. E.; Hedley, D.; Burns, C. O.; Young, R. L.; Butter, E. M.; Vargo, K.; Mulick, J. A.	multicenter	More than one continent	Article	2022	Autism spectrum disorders	Neurodevelopmental diseases or disorders	Interventional studies	J Autism Dev Disord. 2022 Feb 9.	JOURNAL OF AUTISM AND DEVELOPMENTAL DISORDERS	4,291	PSYCHOLOGY, DEVELOPMENTAL	0	Diagnosis	10.1007/s10803-022-05433-1
Clinician Perspectives on Telehealth Assessment of Autism Spectrum Disorder During the COVID-19 Pandemic	Kryszak, E. M.; Albright, C. M.; Fell, L. A.; Butter, E. M.; Kuhlthau, K. A.	United States of America	North America	Article	2022	Autism spectrum disorders	Neurodevelopmental diseases or disorders	Observational studies	J Autism Dev Disord. 2022 Feb 1:1–16.	JOURNAL OF AUTISM AND DEVELOPMENTAL DISORDERS	4,291	PSYCHOLOGY, DEVELOPMENTAL	0	Diagnosis & Treatment/Follow-up	10.1007/s10803-022-05435-z
Telehealth Interventions to Promote Health and Behavior-Related Outcomes in Adolescents with Autism Spectrum Disorder	Lamash, L.; Little, L.; Hen-Herbst, L.	multicenter	More than one continent	Article	2022	Autism spectrum disorders	Neurodevelopmental diseases or disorders	Review/meta-analysis	J Autism Dev Disord. 2022 Jan 21:1–19.	JOURNAL OF AUTISM AND DEVELOPMENTAL DISORDERS	4,291	PSYCHOLOGY, DEVELOPMENTAL	1	Trearment	10.1007/s10803-022-05440-2
A Parent-Mediated Telehealth Program for Children with Autism Spectrum Disorder: Promoting Parents' Ability to Stimulate the Children's Learning, Reduce Parenting Stress, and Boost Their Sense of Parenting Empowerment	Gentile, M.; Messineo, L.; La Guardia, D.; Arrigo, M.; Città, G.; Ayala, A.; Cusimano, G.; Martines, P.; Mendolia, G.; Allegra, M.	Italy	Europe	Article	2022	Autism spectrum disorders	Neurodevelopmental diseases or disorders	Study protocol/Pilot study	J Autism Dev Disord. 2022 Mar 2	JOURNAL OF AUTISM AND DEVELOPMENTAL DISORDERS	4,291	PSYCHOLOGY, DEVELOPMENTAL	0	Follow up	10.1007/s10803-022-05482-6

Brief Report: Feasibility of Delivering the Secret Agent Society Group Social Skills Program via Telehealth During COVID-19: A Pilot Exploration	Mootz, C. A.; Lemelman, A.; Giordano, J.; Winter, J.; Beaumont, R.	United States of America	North America	Article	2022	Autism spectrum disorders	Neurodevelopmental diseases or disorders	Study protocol/Pilot study	J Autism Dev Disord. 2022 May 20:1–6.	JOURNAL OF AUTISM AND DEVELOPMENTAL DISORDERS	4,291	PSYCHOLOGY, DEVELOPMENTAL	0	Trearment	10.1007/s10803-022-05591-2
Utility of Diagnostic Classification for Children 0–5 to Assess Features of Autism: Comparing In-person and COVID-19 Telehealth Evaluations	Holtman, S. J.; Winans, K. S.; Hoch, J. D.	United States of America	North America	Article	2022	Autism spectrum disorders	Neurodevelopmental diseases or disorders	Observational studies	J Autism Dev Disord. 2022 Jun 16:1–12.	JOURNAL OF AUTISM AND DEVELOPMENTAL DISORDERS	4,291	PSYCHOLOGY, DEVELOPMENTAL	0	Diagnosis	10.1007/s10803-022-05606-y
Telehealth Evaluation of Pediatric Neurodevelopmental Disabilities During the COVID-19 Pandemic: Clinician and Caregiver Perspectives	McNally Keehn, R.; Enneking, B.; James, C.; Tang, Q.; Rouse, M.; Hines, E.; Raches, C.; Etling, A.	United States of America	North America	Article	2022	Autism spectrum disorders	Neurodevelopmental diseases or disorders	Observational studies	J Dev Behav Pediatr. 2022 Jun-Jul 01;43(5):262-272.	JOURNAL OF DEVELOPMENTAL AND BEHAVIORAL PEDIATRICS	2,225	PSYCHOLOGY, DEVELOPMENTAL	0	Diagnosis	10.1097/DBP.0000000000001043
Pragmatic adaptations of telehealth-delivered caregiver coaching for children with autism in the context of COVID-19: Perspectives from the United States and South Africa	Franz, L.; Howard, J.; Viljoen, M.; Sikich, L.; Chandrasekhar, T.; Kollins, S. H.; Lee, L.; Ndlovu, M.; Sabatos-DeVito, M.; Seris, N.; Shabalala, N.; Spanos, M.; de Vries, P. J.; Dawson, G.	multicenter	More than one continent	Comment/editorial/letter	2022	Autism spectrum disorders	Neurodevelopmental diseases or disorders	Guidelines/consensus paper	Autism. 2022 Jan;26(1):270-275	AUTISM	5,689	PSYCHOLOGY, DEVELOPMENTAL	3	Follow up	10.1177/13623613211022585
Barriers to Care for Patients Diagnosed with Autism Spectrum Disorder via Telemedicine: A Quality Improvement Project	Landau-Taylor, Jessica; McGivney, Christine; Christiansen, Audrey	United States of America	North America	Conference abstract	2022	Autism spectrum disorders	Neurodevelopmental diseases or disorders	Observational studies	J Dev Behav Pediatr. 43 (2) , pp.E135-E135	JOURNAL OF DEVELOPMENTAL AND BEHAVIORAL PEDIATRICS	2,225	PSYCHOLOGY, DEVELOPMENTAL	0	Diagnosis	NA
The lockdown experience during the Covid-19 outbreak in parents of children with disabilities: Psychological response and tele-rehabilitation	Grumi, Serena; Provenzi, Livio; Aramini, Valentina; Dargenio, Erika; Gardani, Alice; Naboni, Cecilia; Vacchini, Valeria; Manfredini, Vanessa; Pettenati, Giada; Borgatti, Renato; EnForce, Grp	Italy	Europe	Article	2022	Autism spectrum disorders	Neurodevelopmental diseases or disorders	Observational studies	Psicologia Clinica dello Sviluppo 26 (1) , pp.101-124	PSICOLOGIA CLINICA DELLO SVILUPPO	0,11	PSYCHOLOGY, DEVELOPMENTAL	0	Trearment	NA
Increasing resources for autism evaluation and support for under-resourced schools through a state-wide school telehealth initiative	Shahidullah, Jeffrey D.; Brinster, Meredith; Patel, Puja; Cannady, Mariel; Krishnan, Ankita; Talebi, Hani; Mani, Nithya	United States of America	North America	Article	2022	Autism spectrum disorders	neurodevelopmental diseases or disorders	Interventional studies	Psychology in the Schools, 59, 1295– 1307.	PSYCHOLOGY IN THE SCHOOLS	1,774	PSYCHOLOGY, EDUCATIONAL	0	Diagnosis & Treatment/Follow-up	10.1002/pits.22642
Descriptive review of internet-based cognitive behavior therapy on anxiety-related problems in children under the circumstances of COVID-19	Shirotsuki, Kentaro; Sugaya, Nagisa; Nakao, Mutsuhiro	Japan	Asia	Article	2022	Anxiety disorders	Mental disorders	Review/meta-analysis	Biopsychosoc Med. 2022 Jan 10;16(1):3.	BIOPSYCHOSOCIAL MEDICINE	2,373	PSYCHOLOGY, MULTIDISCIPLINARY	0	Trearment	10.1186/s13030-021-00233-y

Seizure- or Epilepsy-Related Emergency Department Visits Before and During the COVID-19 Pandemic - United States, 2019-2021	Sapkota, S.; Caruso, E.; Kobau, R.; Radhakrishnan, L.; Jobst, B.; DeVies, J.; Tian, N.; Hogan, R. E.; Zack, M. M.; Pastula, D. M.	United States of America	North America	Article	2022	Epilepsy	Brain disorders	Observational studies	MMWR Morb Mortal Wkly Rep. 2022 May 27;71(21):703-708	MORBIDITY AND MORTALITY WEEKLY REPORT	17,586	PUBLIC, ENVIRONMENTAL & OCCUPATIONAL HEALTH	0	Follow up	10.15585/mmwr.mm7121a2
Behavioral Assessment and Treatment via Telehealth for Children with Autism: From Local to Global Clinical Applications	Schieltz, K. M.; O'Brien, M. J.; Tsami, L.; Call, N. A.; Lerman, D. C.	United States of America	North America	Article	2022	Autism spectrum disorders	Neurodevelopmental diseases or disorders	Observational studies	Int J Environ Res Public Health. 2022 Feb 15;19(4):2190	INTERNATIONAL JOURNAL OF ENVIRONMENTAL RESEARCH AND PUBLIC HEALTH	3,39	PUBLIC, ENVIRONMENTAL & OCCUPATIONAL HEALTH	1	trearment	10.3390/ijerph19042190
Comparisons between Spirohome and laboratory equipment in children	Kennedy, B.; Bailey, B.; John, M.; Weldon, B.; Robinson, P.; Selvadurai, H.	Australia	Oceania	Conference abstract	2022	Asthma	Asthma	Interventional studies	Respirology 2022 27:SUPPL 1 (15-)	RESPIROLOGY	6,424	RESPIRATORY SYSTEM	0	Follow up	10.1111/resp.14215

Table S3. Category of the Journal in JCR according to publication years

Variable	Publication Years 2020-2022 (*n*=364)	Publication Years 2017-2019 (*n*=137)	p-Value
**Category of the Journal in JCR**			
Allergy	16 (4.40%)	5 (3.65%)	< 0.001
Behavioral Sciences	9 (2.47%)	0 (0.00%)	
Chemistry, Analytical	0 (0.00%)	1 (0.73%)	
Clinical Neurology	13 (3.57%)	2 (1.46%)	
Dentistry, Oral Surgery & Medicine	7 (1.92%)	3 (2.19%)	
Dermatology	7 (1.92%)	4 (2.92%)	
Education, Special	10 (2.75%)	1 (0.73%)	
Endocrinology & Metabolism	70 (19.23%)	15 (10.95%)	
Environmental Studies	1 (0.27%)	0 (0.00%)	
Genetics & Heredity	2 (0.55)	0 (0.00%)	
Health Care Sciences & Services	25 (6.87%)	15 (10.95%)	
Health Policy & Services	5 (1.37%)	3 (2.19%)	
Medical Informatics	14 (3.85%)	11 (8.03%)	
Medicine, General & Internal	10 (2.75%)	8 (5.83%)	
Medicine, Research & Experimental	4 (1.10%)	3 (2.19%)	
Multidisciplinary Sciences	1 (0.27%)	3 (2.19%)	
Neurosciences	10 (2.75%)	1 (0.73%)	
Nursing	4 (1.10%)	3 (2.19%)	
Ophthalmology	0 (0.00%)	1 (0.73%)	
Pediatrics	70 (19.23%)	16 (11.68%)	
Pharmacology & Pharmacy	2 (0.55%)	2 (1.46%)	
Psychiatry	27 (7.42%)	11 (8.03%)	
Psychology, Clinical	10 (2.75%)	5 (3.65%)	
Psychology, Developmental	33 (9.07%)	5 (3.65%)	
Psychology, Educational	1 (0,27%)	0 (0.00%)	
Psychology, Multidisciplinary	1 (0,27%)	0 (0.00%)	
Public, Environmental & Occupational Health	6 (1.65%)	6 (4.38%)	
Rehabilitation	0 (0.00%)	2 (1.46%)	
Respiratory System	6 (1.65%)	10 (7.30%)	
Urology & Nephrology	0 (0.00%)	1 (0.73%)	

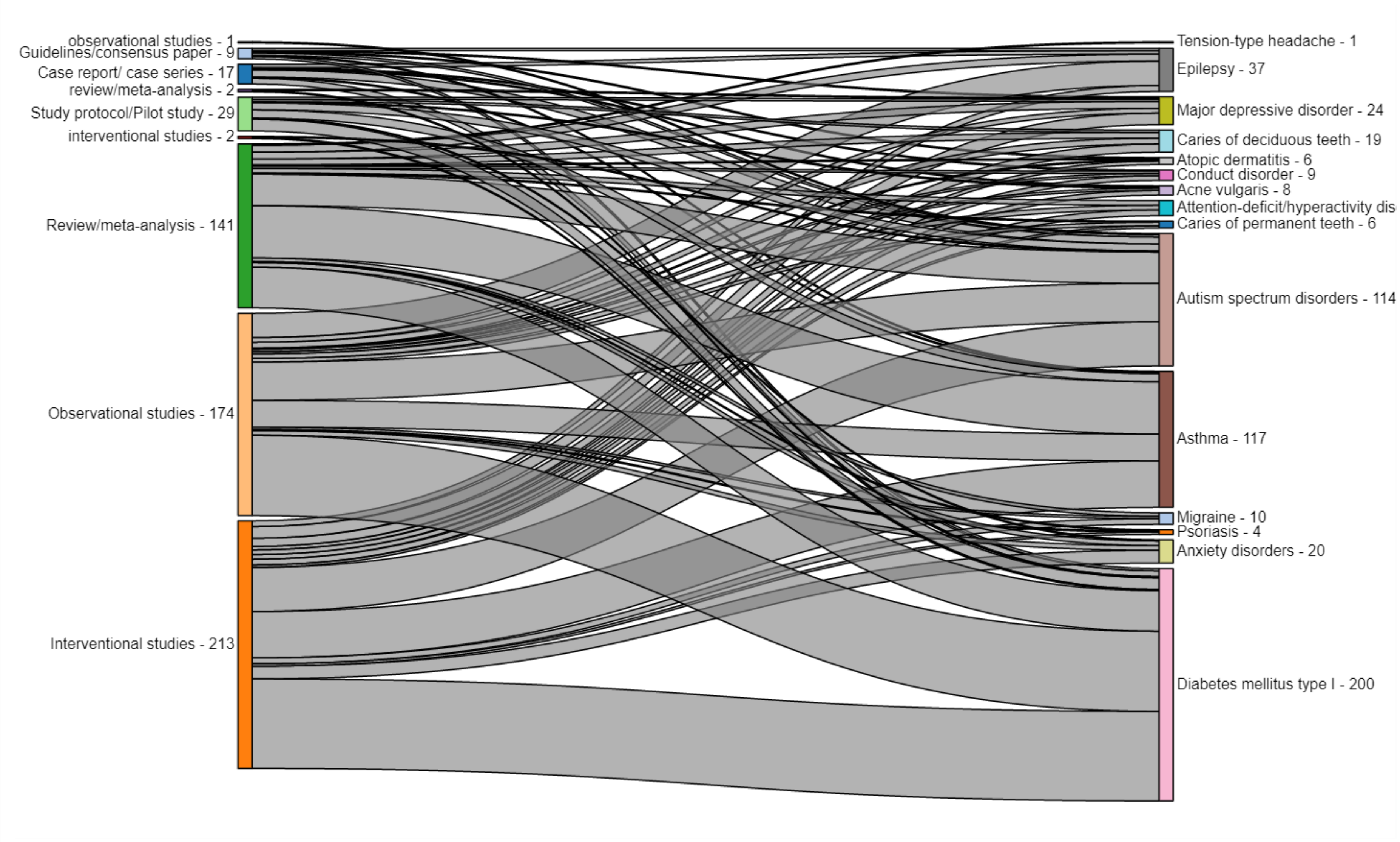

Figure S1. Sankey diagram visualization of type of studies and topics
